# Advances in High‐Entropy Catalysts for Lithium–Sulfur Batteries: Design Principles, Recent Progress, and Prospects

**DOI:** 10.1002/advs.202511072

**Published:** 2025-08-12

**Authors:** Ruohan Hou, Yixin Wei, Jiaxiang Zhang, Jiaying Chen, Shaojie Chen, Shuaiting Sun, Guosheng Shao, Peng Zhang

**Affiliations:** ^1^ State Center for International Cooperation on Designer Low‐carbon & Environmental Materials (CDLCEM) School of Materials Science and Engineering Zhengzhou University 100 Kexue Avenue Zhengzhou 450001 China; ^2^ Zhengzhou Materials Genome Institute (ZMGI) Xingyang Zhengzhou 450100 China

**Keywords:** catalyst, high‐entropy, Li–S batteries, polysulfide conversion, shuttle effect

## Abstract

Lithium–sulfur (Li–S) batteries are regarded as one of the most promising next‐generation energy storage technologies due to their exceptionally high theoretical energy density, cost‐effectiveness, and environmental sustainability. Nevertheless, their practical deployment is significantly constrained by several challenges, including the intrinsic low conductivity of sulfur, the shuttle effect of lithium polysulfides (LiPS), sluggish redox kinetics, and instability of the Li anode. To overcome these limitations, the integration of catalytic materials has emerged as an effective strategy to accelerate sulfur redox reactions, promote LiPS conversion, and enhance cycling stability. Recently, high‐entropy catalysts (HEC), comprising five or more metallic elements in near‐equimolar ratios, have garnered increasing attention owing to their entropy‐stabilized structures, abundant active sites, and tunable electronic properties. This review presents a comprehensive overview of the design principles, synthesis methods, and electrochemical applications of various HEC families, including high‐entropy alloys, oxides, sulfides, nitrides, phosphides, MXenes, and Prussian blue analogues, in Li–S battery systems. The synergistic effects arising from multicomponent interactions, structural advantages, and underlying catalytic mechanisms are systematically discussed. Finally, key challenges such as scalable synthesis, in‐depth mechanistic elucidation, and rational compositional design are addressed, along with future directions aimed at advancing high‐performance HEC‐based Li–S battery systems.

## Graphical abstract

This review highlights recent progress in high‐entropy catalysts for Lithium–sulfur (Li–S) batteries, covering their structural features, catalytic functions, synthesis strategies, and representative material systems, including high‐entropy alloys, oxides, nitrides, sulfides, phosphides, and MXene. Challenges and future directions are also discussed to guide the rational design of high‐performance Li–S battery high‐entropy catalysts.

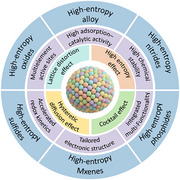

## 1 Introduction

The ever‐increasing demand for high‐energy, cost‐effective, and environmentally sustainable energy storage systems has driven the exploration of advanced battery chemistries beyond conventional lithium‐ion batteries.^[^ ^1^ ^]^ Among the various candidates, Lithium–sulfur (Li–S) batteries have garnered significant attention due to their exceptional theoretical energy density (≈2600 Wh kg^−1^), high specific capacity (1675 mAh g^−1^), and the natural abundance and low cost of active sulfur.^[^ ^2^ ^]^ These features make Li–S systems highly attractive for next‐generation applications ranging from electric vehicles to large‐scale grid storage. However, their practical implementation remains hampered by persistent challenges, including the poor electrical conductivity of sulfur and its discharge products, severe volumetric expansion during cycling, the shuttle effect induced by soluble lithium polysulfides (LiPS), sluggish redox kinetics, and the instability of the Li metal anode.^[^ ^3^ ^]^ Among these challenges, the continuous shuttling of soluble LiPS between the cathode and anode is considered the primary bottleneck behind a series of detrimental effects, including reduced practical discharge capacity, active material loss, poor cycling stability, low coulombic efficiency, and self‐discharge.^[^ ^4^ ^]^

To overcome these multifaceted challenges, considerable research efforts have been dedicated to the development of functional materials capable of regulating sulfur chemistry and stabilizing battery components during cycling.^[^ ^5^ ^]^ Among the various strategies, catalytic modulation of sulfur species has proven particularly effective in enhancing redox kinetics, anchoring soluble intermediates, and promoting the reversible formation and decomposition of Li_2_S.^[^ ^6^ ^]^ Recently, the incorporation of transition metal‐based catalysts into Li–S batteries has been widely recognized as an effective strategy to accelerate electrochemical reaction kinetics and mitigate the shuttle effect of LiPS.^[^ ^7^ ^]^ In this regard, various transition metal compounds, such as oxides, nitrides, sulfides, and MXene, have been extensively developed, and significantly improved the practical capacity and cycling stability of Li–S batteries.^[^ ^8^ ^]^ Currently, multifunctional compounds based on multi‐component metals or carbon‐based composite materials represent the dominant design approach for high‐performance catalysts.^[^ ^9^ ^]^ The entropy‐enhancement effect in multi‐component systems has been shown to be an effective strategy for improving energy storage and conversion efficiency. Numerous recent theoretical and experimental studies have demonstrated that, compared to their low‐entropy counterparts, high‐entropy catalysts (HEC) exhibit higher LiPS adsorption efficiency and superior cycle stability.^[^ ^10^ ^]^ This is primarily due to the synergy between the multi‐component metals in HEC and the disordered atomic arrangement on the active surface, which provides abundant active sites for chemical anchoring and catalytic LiPS conversion.^[^ ^11^ ^]^

**Figure 1:**
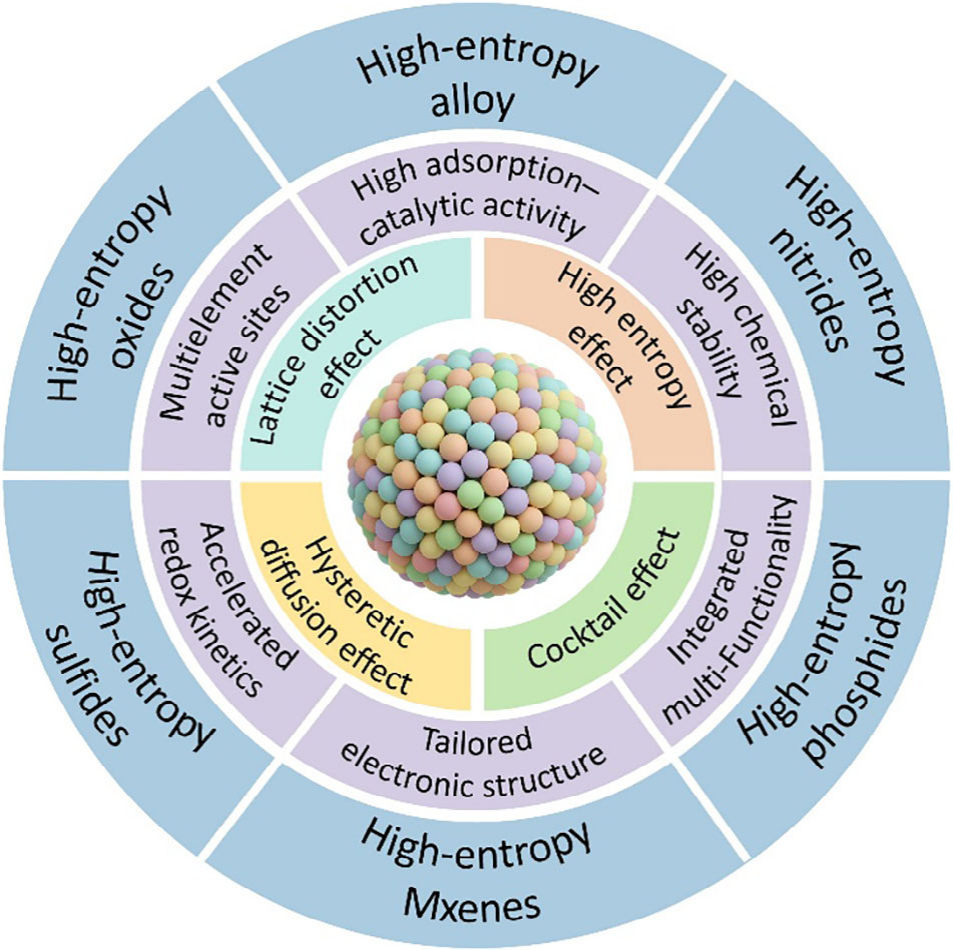
The application of high‐entropy catalyst material systems in Li–S batteries.

Based on the corresponding component composition definition, high‐entropy materials (HEM) are generally composed of five or more metallic elements in near‐equimolar or compositionally optimized ratios, forming a single‐phase structure stabilized by high configurational entropy.^[^ ^12^ ^]^ This entropy‐driven stabilization allows for the formation of solid solutions even among elements with significant disparities in atomic size, valence states, or electronegativity. The resultant materials are characterized by disordered atomic arrangements, diverse coordination environments, and tunable electronic structures.^[^ ^13^ ^]^ These attributes collectively endow HEC with several advantages for electrocatalytic applications: a rich array of active sites, enhanced binding interactions with LiPS, flexible redox behavior, and superior structural stability.^[^ ^11^ ^]^ As a result, HEC have emerged as promising multifunctional electrocatalysts in Li–S batteries, capable of mediating LiPS conversion, suppressing shuttle effects, and maintaining long‐term electrochemical stability(**Figure** **1**). Initially conceived in the field of metallurgy, the high‐entropy concept has since been extended to a variety of compound material systems relevant to energy catalysis, including high‐entropy oxides (HEO), nitrides (HEN), phosphides (HEP) and others.^[^ ^14^ ^]^ Despite this growing interest, the application of HEC in Li–S batteries is still in its early stages. Several critical challenges remain, including the complexity of structure–function relationships, the lack of predictive composition‐performance models, and difficulties associated with scalable synthesis. Furthermore, the fundamental mechanisms through which configurational entropy and chemical disorder contribute to catalytic enhancement remain insufficiently understood. Therefore, continued investigation is needed to elucidate the dynamic interactions between HEM systems and sulfur species under realistic electrochemical conditions.

This review aims to provide a timely and comprehensive overview of the current state of high‐entropy catalyst research in the context of Li–S batteries. Beginning with a concise introduction to the thermodynamic principles and fundamental characteristics of HEC, the review explores their structural and functional advantages. In addition, a range of synthesis strategies and the working mechanism of Li–S battery systems are introduced. The core focus of this review centers on recent advancements in the application of various HEC families for Li–S battery systems. For each category, representative studies are discussed with respect to compositional design, electrochemical performance, and mechanistic insights. Finally, in light of the current research limitations, future directions are outlined, including high‐throughput compositional screening, in situ mechanistic characterization, and the development of environmentally friendly and scalable synthesis strategies, among others. Ultimately, the goal is to promote high‐entropy catalytic strategies that can enable the development of high‐performance, durable, and commercially viable Li–S energy storage systems.

## 2 Design Principles and Intrinsic Advantages of High‐Entropy Catalysts

High‐entropy catalysts (HEC), derived from the broader concept of high‐entropy materials (HEM), represent a class of multicomponent systems where five or more principal elements are incorporated in near‐equiatomic or specifically tuned ratios.^[^ ^15^ ^]^ Their emergence has revolutionized the field of materials science and catalysis by introducing an unconventional path to tuning material properties via compositional complexity and configurational entropy stabilization. This section outlines the foundational principles guiding HEC design, summarizes their intrinsic structural and functional advantages, and provides a systematic overview of current synthesis methodologies.

### 2.1 Definition and Conceptual Framework of High‐Entropy Catalysts

HEC are a class of catalytically active materials that consist of five or more principal elements mixed in near‐equimolar or equiatomic proportions, resulting in a single‐phase solid solution stabilized primarily by high configurational entropy. This concept originated from the broader field of HEM, initially proposed by Yeh et al. and Cantor et al. in 2004, which aimed to explore the stabilization of multiprincipal element alloys (MPEAs) through entropy‐driven thermodynamic mechanisms.^[^ ^16^ ^]^ The defining characteristic of HEC lies in their multi‐elemental composition, typically incorporating diverse metallic or semi‐metallic elements without a dominant host lattice. Unlike traditional catalysts, which are often based on binary or ternary systems with clearly defined active sites, HEC present a compositionally disordered lattice in which each atomic site is surrounded by a random ensemble of neighboring elements.^[^ ^15^ ^]^ This chemical disorder creates a vast array of local atomic environments and electronic configurations, offering a combinatorial platform for tuning catalytic performance at the atomic scale.

Thermodynamically, the formation of high‐entropy phases is governed by the Gibbs free energy expression:

(1) $$\Delta G_{\mathrm{mix}}=\Delta H_{\mathrm{mix}}-T\Delta S_{\mathrm{config}}$$

where *ΔG_mix_* is the Gibbs free energy change upon mixing; *ΔH_mix_* is the enthalpy of mixing, reflecting the energy change due to interactions between dissimilar atoms; 𝑇 is the absolute temperature (in kelvin); *ΔS_config_* is the configurational entropy, which accounts for the disorder introduced by multiple principal elements.^[^ ^17^ ^]^

The configurational entropy, in particular, is given by the Boltzmann formula:^[^ ^18^ ^]^

(2) $$\Delta S_{\mathrm{config}}=-R\Sigma_{i=1}^{n}\times_{i}\mathrm{In}x_{i}$$

where R is the universal gas constant (8.314 J mol^−1^ K^−1^), *n* is the total number of constituent elements, *x_i_* is the atomic fraction (mole fraction) of the *i*th element. Moreover, the configurational entropy term is maximized when all component elements are present in equal molar fractions (*x_i_* = 1/n). This high entropy can offset moderately positive enthalpies of mixing, thereby stabilizing single‐phase structures (e.g., face‐centered cubic (FCC), body‐centered cubic (BCC), or rock‐salt lattices) that would otherwise decompose into multiple phases.^[^ ^18^ ^]^

Although there is no universally fixed stoichiometric threshold, a working definition commonly adopted in recent report identifies HEC as those containing five or more elements each in a concentration between 5 and 35 at%, forming a single‐phase solid solution with catalytically accessible surfaces.^[^ ^19^ ^]^ This definition excludes phase‐segregated alloys and traditional solid solutions with a dominant host. In addition to the compositional criterion, high‐entropy materials are thermodynamically characterized by their ideal configurational entropy (Δ*S_config_*). According to widely accepted literature standards, materials with Δ*S_config_* ≥ 1.5R are classified as high‐entropy systems. Those with Δ*S_config_* values between 1.0R and 1.5R are generally considered medium‐entropy, while materials with values below 1.0R are categorized as low‐entropy. The scope of HEC has expanded rapidly in recent years to include high‐entropy oxides (HEO), sulfides (HES), nitrides (HEN), carbides (HECbs), and even high‐entropy single‐atom catalysts (HE‐SAC), each offering distinct advantages in terms of stability, tunability, and multifunctionality.^[^ ^20^ ^]^ These developments are reshaping catalyst design paradigms, challenging traditional scaling relations, and enabling multifunctional catalytic systems with tunable activity, selectivity, and stability.

### 2.2 Structural and Functional Advantages of High‐Entropy Catalysts

HEC, characterized by the incorporation of five or more principal metallic elements in near‐equimolar ratios, represent a paradigm shift in the design of heterogeneous catalysts. The absence of a dominant host element in HEC results in a complex, disordered configuration of atoms, endowing the material with a suite of catalytic features that are fundamentally different from those of conventional binary or ternary catalysts.

#### 2.2.1 Multielement Active Sites

The most notable advantage of HEC lies in their multielement surface composition, which generates a vast number of distinct atomic configurations and active sites. Each surface atom resides in a unique coordination environment, enabling a broad spectrum of adsorption geometries and reaction pathways. This high degree of surface heterogeneity not only broadens the catalytic landscape but also promotes synergistic interactions between neighboring atoms, enhancing the overall reactivity and selectivity of the catalyst.^[^ ^21^ ^]^ For instance, Hao et al. rationally designed a FeCoNiXRu (X = Cu, Cr, Mn) high‐entropy alloys (HEA) system featuring a diverse array of active sites with differentiated adsorption capabilities for multiple reaction intermediates involved in the hydrogen evolution reaction (HER) and water dissociation processes under alkaline conditions. Density functional theory (DFT) calculations and spectroscopic analyses reveal that the electronegativity disparities among the constituent elements induce significant local charge redistribution, leading to the formation of highly active Co and Ru sites. These sites exhibit optimal binding energies for both *OH and *H intermediates, thereby facilitating the key water dissociation step and accelerating reaction kinetics.^[^ ^22^ ^]^

#### 2.2.2 Tunable Electronic Structure

Furthermore, the inherent chemical disorder in HEC leads to a significant redistribution of the electronic structure. The mixing of multiple metallic elements results in a broadened d‐band and the emergence of localized states that can modulate the binding energies of key reaction intermediates.^[^ ^23^ ^]^ This redistribution allows the fine‐tuning of adsorption energies and can effectively break conventional scaling relations, offering a pathway to optimizing activity and selectivity beyond the limitations of single‐metal systems.^[^ ^24^ ^]^ Moreover, the presence of diverse electronic environments contributes to enhanced conductivity in certain HEC, facilitating rapid charge transfer during catalytic operation.^[^ ^25^ ^]^ Tian et al. rationally design a series of alloy electrocatalysts with broadly distributed d‐band centers based on an HEA strategy, aiming to systematically investigate the applicability of the Sabatier principle in lithium–oxygen batteries (LOB). By precisely modulating the elemental composition, a series of catalysts with the d‐band center positions decreasing in sequence were constructed: HEA (FeCoNiMn) > HEAPtIr (FeCoNiMnPtIr) > HEAIr (FeCoNiMnPIr) > HEAPt (FeCoNiMnPIr). Among the designed catalysts, HEAPtIr exhibits the most balanced adsorption capability for oxygen intermediates, thereby achieving the highest catalytic performance. Combined DFT calculations and electrochemical evaluations reveal that an excessively high d‐band center leads to overly strong binding with oxygen species, which impedes desorption kinetics, deactivates surface sites, and ultimately suppresses reaction progression. In contrast, an overly low d‐band center fails to sufficiently retain key intermediates, resulting in premature product loss, unstable reaction pathways, and severe surface passivation.^[^ ^26^ ^]^ Moreover, Lv et al. achieved a controllable redistribution of electron density around Pt‐Rh active sites by introducing both low‐electronegativity elements (Ga, Ni) and high‐electronegativity elements (W) into the PtRhGaNiW HEA framework. This electronic modulation expanded the d‐orbital interaction window and optimized the adsorption‐desorption balance of key reaction intermediates. As a result, the synthesized PtRh‐HEA nanowires exhibited markedly enhanced methanol oxidation reaction (MOR) performance, with a peak current density of 5.61 mA cm^−2^ higher than that of the commercial Pt/C catalyst (0.57 mA cm^−2^) and the PtRh binary alloy (1.66 mA cm^−2^), respectively. Additionally, the catalyst demonstrated excellent long‐term operational stability and superior resistance to surface passivation.^[^ ^27^ ^]^

#### 2.2.3 Lattice Distortion and Surface Strain Engineering

In addition to electronic tunability, HEC exhibit severe lattice distortions due to the mismatch in atomic radii and bonding preferences among the constituent elements. These distortions result in local strain fields and variations in bond lengths that can modulate the surface energy landscape.^[^ ^28^ ^]^ Strain engineering at the atomic scale has been shown to significantly alter catalytic performance by influencing the activation energies of rate‐determining steps and stabilizing key reaction intermediates. Such atomic‐scale distortions also promote the formation of undercoordinated sites, steps, and kinks that often serve as high‐activity centers in heterogeneous catalysis.^[^ ^19^, ^29^ ^]^ For example, Bao et al. proposed a strain engineering strategy to modulate the lattice strain and electronic configuration of a FeCoNiCuZn high‐entropy alloy (LiTM) by incorporating Li, thereby enhancing its electrocatalytic performance. Systematic experimental characterization and DFT simulations demonstrated that lithium incorporation induced tensile strain in the LiTM lattice, effectively optimizing the position of the d‐band center and improving electrical conductivity. These modifications resulted in more favorable adsorption energies for key reaction intermediates, thus accelerating the overall catalytic kinetics. The optimized LiTM‐25 catalyst exhibited excellent OER and HER activities in alkaline brine, delivering ultra‐low overpotentials of 265 and 42 mV at a current density of 10 mA cm^−2^, respectively.^[^ ^30^ ^]^

#### 2.2.4 Catalytic Selectivity

Another salient feature of HEC is their intrinsic electronic heterogeneity, arising from the varied atomic composition and coordination at the surface. Unlike traditional catalysts with relatively uniform adsorption properties, HEC offer a continuous distribution of binding energies for reaction intermediates.^[^ ^31^ ^]^ This diversity allows for a more adaptable catalytic surface where different reaction steps may be optimized by distinct atomic ensembles.^[^ ^31^, ^32^ ^]^ Consequently, HEC can support bifunctional or even multifunctional catalytic behaviors, enhancing overall efficiency in complex, multi‐step processes. For instance, the FeNiCoCrRu HEA catalyst fabricated via laser induction by Xie et al. demonstrated outstanding bifunctional electrocatalytic activity in both alkaline freshwater and seawater environments. Notably, the catalyst delivered a low overpotential of 148 mV at a high current density of 600 mA cm^−2^ for the HER, and 353 mV at 300 mA cm^−2^ for the OER in alkaline seawater. In situ Raman spectroscopy was employed to elucidate the reaction mechanisms. For HER, Ni sites primarily facilitated the adsorption and dissociation of H_2_O, while Ru sites promoted the recombination of H intermediates into H_2_. For OER, Ni sites enhanced the deprotonation of OH species, leading to the formation of O intermediates and accelerating O_2_ evolution.^[^ ^33^ ^]^ Additionally, Song et al. employed a vacuum‐assisted dealloying strategy to synthesize ZnNiCoIrMn HEA catalysts, which exhibited excellent bifunctional electrochemical performance. The catalyst demonstrated a low overpotential of 50 mV at 50 mA cm^−2^ for the HER and 237 mV at 10 mA cm^−2^ for the OER. Notably, the incorporation of Mn into the HEA matrix effectively modulated the local electronic structure of the Ir active sites. DFT calculations revealed that Mn doping led to a significant downward shift of the Ir d‐band center relative to the Fermi level. This electronic modulation reduced the adsorption energy of key reaction intermediates, thereby accelerating catalytic kinetics and enhancing overall bifunctional activity. The adaptability of HEC in terms of binding strength and selectivity makes them promising platforms for cascade catalysis and multifunctional systems.^[^ ^34^ ^]^

#### 2.2.5 High Chemical Stability

One of the most compelling advantages of high‐entropy catalysts lies in their exceptional thermal, chemical, and structural stability under harsh catalytic conditions. This high stability is fundamentally derived from the high configurational entropy introduced by the near‐equimolar incorporation of multiple principal elements.^[^ ^35^ ^]^ A high configurational entropy can significantly lower the Gibbs free energy, thus stabilizing single‐phase solid solutions even at complex chemical environment.^[^ ^36^ ^]^ Additionally, the sluggish diffusion effect in high‐entropy materials plays a crucial role in retarding atom migration, thereby enhancing resistance against sintering and thermal degradation. The random lattice distortion and complex atomic interactions further create kinetic barriers for phase transformation or surface atom mobility, enabling long‐term stability during high‐temperature and redox cycles. In electrochemical environments, high‐entropy electrocatalysts exhibit remarkable cycling durability.^[^ ^37^ ^]^ As an example, Li et al. synthesized a 10‐element HEO catalyst via a rapid high‐temperature heating method, effectively suppressing elemental segregation and phase decomposition through the entropy stabilization effect. This strategy significantly enhanced the chemical stability of the catalyst under practical operating conditions. The optimized 10‐element HEO/C catalyst exhibited remarkable electrocatalytic activity, and more importantly, demonstrated outstanding long‐term durability for the ORR, maintaining 92% and 86% of its initial activity after 12 and 100 h of continuous operation, respectively.^[^ ^38^ ^]^ The lack of dominant dissolution‐prone elements and the mutual stabilization among elements reduce corrosion and leaching under electrochemical polarization. Therefore, the synergistic interplay of thermodynamic entropy, sluggish diffusion, and multi‐elemental mutual stabilization renders high‐entropy catalysts inherently more stable than their low‐entropy counterparts, making them ideal for long‐term catalysis and electrochemical energy conversion processes.

### 2.3 Core Effects Underpinning High‐Entropy Catalytic Behavior

HEC derive their distinctive catalytic properties not solely from their multicomponent composition, but from a set of intrinsic physicochemical effects arising from compositional complexity and atomic‐scale disorder. These include the high‐entropy effect, cocktail effect, sluggish diffusion effect, and lattice distortion effect. Collectively, these four effects dictate the stability, activity, selectivity, and durability of HEC and are particularly crucial for their performance in Li–S batteries.

#### 2.3.1 High‐Entropy Effect

The high‐entropy effect is the thermodynamic foundation of high‐entropy materials. It refers to the stabilization of a single‐phase solid solution via maximized configurational entropy, particularly when five or more elements are incorporated in near‐equimolar ratios. According to the Gibbs free energy relation (Δ*G_mix_ =* Δ*H_mix_‐T*Δ*S_config_*), a sufficiently large entropy term (Δ*S_config_*) can counterbalance positive mixing enthalpy (Δ*H_mix_*), thereby preventing phase separation even among thermodynamically immiscible elements. In Li–S batteries, this effect ensures structural uniformity during prolonged cycling and suppresses elemental segregation or catalyst deactivation under harsh complex redox reaction environment. The resulting homogeneous distribution of active sites and a stable host matrix enhances electrochemical reversibility and long‐term durability.

#### 2.3.2 Cocktail Effect

The cocktail effect, unique to multi‐principal element systems, refers to the emergence of synergistic or non‐linear enhancement in material properties that are not predictable by simple averaging of the constituent elements. In HEC, the co‐existence of diverse elements with varying electronegativities, oxidation states, and bonding tendencies generates a highly heterogeneous electronic and chemical environment. This diversity facilitates multiple reaction pathways, broadens the adsorption energy window, and improves selectivity toward sulfur redox intermediates (e.g., Li_2_S_6_, Li_2_S_2_/Li_2_S). For Li–S batteries, the cocktail effect enhances the chemisorption of LiPS, improved electron/ion transport, and dual‐function catalytic behavior, promoting both reduction (S → Li_2_S) and oxidation (Li_2_S → LiPS) steps with minimal overpotential.

#### 2.3.3 Hysteresis Diffusion Effect

The sluggish diffusion effect refers to the reduced atomic mobility within the high‐entropy matrix, arising from pronounced atomic‐scale disorder and lattice complexity. The incorporation of elements with varying atomic radii and bonding characteristics generates a highly rugged energy landscape that hinders atomic migration. This intrinsic kinetic sluggishness plays a pivotal role in enhancing structural stability by suppressing elemental segregation, surface reconstruction, and phase transformations under electrochemical or thermal stress. In high‐entropy catalysts, this effect aids in preserving the active phase, retaining nanoscale features, and preventing catalyst deactivation during prolonged operation. When applied to Li–S batteries, it helps maintain a robust catalytic interface, supports sustained polysulfide conversion, and improves overall cycle life.

#### 2.3.4 Lattice Distortion Effect

Lattice distortion is an inherent characteristic of HEC, resulting from the inclusion of atoms with varying sizes, valence states, and coordination geometries. These distortions introduce localized strain, alter bond lengths and angles, and generate high‐energy surface features, such as steps, kinks, and under‐coordinated atoms, that serve as catalytic “hot spots.” Additionally, strain fields can modulate the d‐band center of surface atoms, thereby fine‐tuning their adsorption/desorption interactions with redox intermediates. In Li–S batteries, lattice distortion accelerates the conversion of LiPS ↔ Li_2_S/Li_2_S_2_ by stabilizing transient species and lowering activation barriers. It also helps buffer mechanical stress induced by the volumetric expansion of sulfur species during cycling.

**Figure 2:**
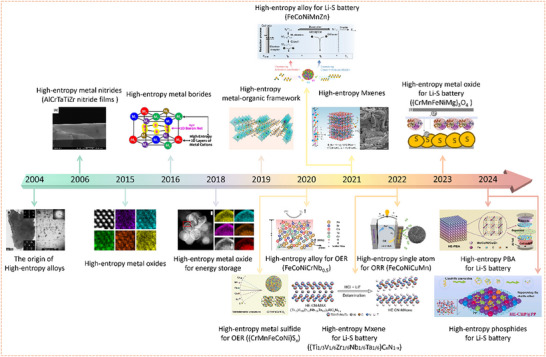
Development timeline of high‐entropy catalysts and their critical applications in energy‐related catalytic systems. Representative work inserted includes: Chang et al. reported the origin of the synthesis of HEA. Reproduced with permission.^[^ ^16b^ ^]^ Copyright 2004, Wiley‐VCH. Report on high‐entropy nitrides prepared by Chang et al. Reproduced with permission.^[^ ^39^ ^]^ Copyright 2006, Elsevier B.V. Christina M. Rost et al. reported on the concept of HEO. Reproduced with permission.^[^ ^40^ ^]^ Copyright 2015, Springer Nature. Joshua Gild et al. reported on the synthesis of HEB. Reproduced with permission.^[^ ^41^ ^]^ Copyright 2016, Springer Nature. Abhishek Sarkar et al. reported on the application of HEO in the field of energy storage. Reproduced with permission.^[^ ^42^ ^]^ Copyright 2018, Springer Nature. Zhao et al. synthesized HE‐MOF and employed them as catalysts for the OER. Reproduced with permission.^[^ ^43^ ^]^ Copyright 2019, Royal Society of Chemistry. Ding et al. reported the application of HEA as efficient catalysts for the OER. Reproduced with permission.^[^ ^44^ ^]^ Copyright 2020, Wiley‐VCH. Cui et al. synthesized HES and demonstrated their effectiveness as catalysts in the OER process. Reproduced with permission.^[^ ^45^ ^]^ Copyright 2020, Wiley‐VCH. Li et al. prepared an HEA catalyst to improve the kinetics of sulfur redox reactions. Reproduced with permission.^[^ ^46^ ^]^ Copyright 2021, Elsevier B.V. Nemani et al. reported the synthesis of HE‐MXene. Reproduced with permission.^[^ ^47^ ^]^ Copyright 2021, American Chemical Society. Rao et al. synthesized a HE‐SA catalyst, which was applied in the ORR. Reproduced with permission.^[^ ^48^ ^]^ Copyright 2022, Springer Nature. Li et al. prepared HE‐MXenes to improve the electrochemical performance of Li–S batteries. Reproduced with permission.^[^ ^49^ ^]^ Copyright 2021, Wiley‐VCH. Lin et al. synthesized a HEO and employed it as a separator modification layer to enhance the performance of Li–S batteries. Reproduced with permission.^[^ ^50^ ^]^ Copyright 2023, American Chemical Society. Shen et al. synthesized HE‐PBA as a multifunctional regulator to improve the performance of Li–S batteries. Reproduced with permission.^[^ ^51^ ^]^ Copyright 2024, Royal Society of Chemistry. Zhu et al. synthesized a HEP catalyst as a separator modification layer in the Li–S battery system. Reproduced with permission.^[^ ^52^ ^]^ Copyright 2024, Elsevier Inc.

Owing to the above features, HEC have demonstrated broad applicability across various catalytic reactions, including thermocatalytic CO oxidation, electrocatalytic HER/OER, nitrogen reduction (NRR), methanol steam reforming (MSR), and more recently, sulfur redox reactions in Li–S batteries. Their structural stability and compositional tunability make them particularly well‐suited for harsh or corrosive reaction environments. As illustrated in **Figure** **2**, the timeline outlines the developmental trajectory of HEM, highlighting representative studies on HEC from the initial emergence of HEA to their subsequent applications in energy storage and conversion. In the following sections, this review will focus on the catalytic behavior of high‐entropy catalysts in Li–S battery systems, with particular emphasis on their role in enhancing the kinetics of sulfur redox reactions.

## 3 Working Principle, Advantages, and Challenges of Lithium–Sulfur Batteries

### 3.1 Working Principle and Advantages of Lithium–Sulfur Batteries

Li–S batteries have garnered significant attention as next‐generation energy storage systems due to their remarkable theoretical performance metrics and the earth abundance of active materials. One of the most notable advantages is their exceptionally high theoretical energy density of approximately 2600 Wh kg^−1^, which is nearly five times greater than that of conventional lithium‐ion batteries (LIBs) based on intercalation‐type cathode materials such as LiCoO_2_ or LiFePO_4_.^[^ ^53^ ^]^ This intrinsic advantage stems from the high specific capacity of sulfur (1675 mAh·g^−1^) and its low atomic weight. Another key benefit is the natural abundance, low cost, and environmental benignity of sulfur.^[^ ^54^ ^]^ Elemental sulfur is an industrial byproduct, particularly from petroleum refining processes, and is available in large quantities at minimal cost. This contrasts with LIB cathodes that depend on transition metals such as cobalt and nickel, which are relatively scarce, geopolitically sensitive, and environmentally taxing to mine.^[^ ^55^ ^]^ Thus, Li–S batteries are especially attractive for sustainable and large‐scale energy storage applications, including grid‐level storage and electric transportation.

**Figure 3:**
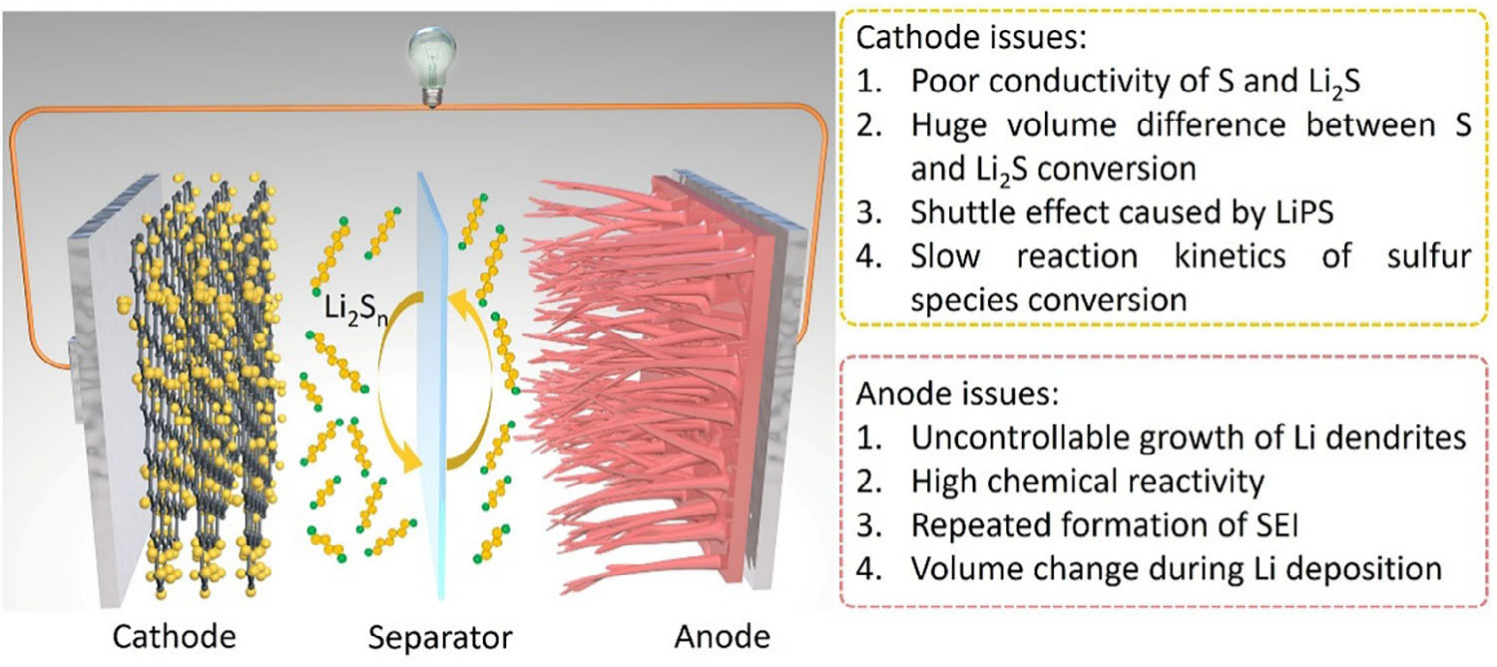
Schematic illustration of the working mechanism of a Li–S battery and the major challenges associated with the cathode and anode.

Additionally, sulfur is a low‐cost, naturally abundant, and environmentally benign element, often derived as a byproduct from petroleum refining. This abundance alleviates concerns over critical material supply chains—an increasingly pressing issue in Li‐ion battery systems that rely heavily on cobalt or nickel‐based cathodes.^[^ ^56^ ^]^ Furthermore, the Li metal anode provides a high specific capacity (3860 mAh g^−1^), further contributing to the overall gravimetric energy density of the full cell. A typical Li–S battery is composed of a metallic Li anode, a sulfur‐based composite cathode, a porous separator, and a liquid electrolyte generally consisting of lithium salt (e.g., LiTFSI) dissolved in ether‐based solvents. Unlike conventional intercalation‐type Li‐ion batteries, Li–S cells operate via a conversion‐type mechanism that entails the full reduction of elemental sulfur (S_8_) to lithium sulfide (Li_2_S), involving complex multistep redox reactions and soluble intermediate species (**Figure** **3**).

During the discharge process, elemental sulfur undergoes successive reduction through a series of lithium polysulfides (LiPS) intermediates (Li_2_S_n_, 2 ≤ *n* ≤ 8) before forming solid Li_2_S as the final discharge product. This process involves the transfer of 16 electrons per molecule of S_8_:

(3) $$\mathrm{Discharge}:S8+16Li^{+}+16e^{-}\to 8Li_{2}S$$

These reactions occur in two distinct voltage plateaus (≈2.4 and ≈2.1 V vs. Li/Li^+^), corresponding to the formation of long‐chain (Li_2_S_4_–Li_2_S_8_) and short‐chain (Li_2_S–Li_2_S_2_) polysulfides, respectively.^[^ ^57^ ^]^ The charging reaction is the reverse reaction of the discharge reaction.

### 3.2 Main Challenges of Lithium–Sulfur Batteries

The outstanding theoretical energy density, low raw material cost, and sustainable chemistry of Li–S batteries make them highly promising candidates for both portable electronics and large‐scale energy storage. Nevertheless, their commercialization remains largely hindered by a series of intrinsic challenges associated with the complex redox chemistry of sulfur species, unstable electrode–electrolyte interfaces, and safety concerns arising from Li metal anodes. A comprehensive understanding of these issues is essential for the rational design of advanced materials and cell configurations.

#### 3.2.1 Poor Electronic Conductivity of Sulfur and Discharge Products

Sulfur and its lithiation end‐products (Li_2_S and Li_2_S_2_) are intrinsically insulative, with electronic conductivities on the order of 10^−30^ and 10^−13^ S cm^−1^, respectively.^[^ ^58^ ^]^ This severely limits electron transport within the cathode during charge and discharge processes, necessitating the incorporation of high surface area conductive carbon additives or frameworks to facilitate electron percolation. However, excessive conductive additives lower the sulfur content, reducing the practical specific energy of the device. Furthermore, uniform dispersion of insulating sulfur and Li_2_S phases within the conductive matrix is difficult to maintain during repeated cycling, leading to inhomogeneous electrochemical activity and degraded performance.

#### 3.2.2 Volumetric Expansion and Cathode Structural Collapse

The conversion of elemental S to Li_2_S involves a significant volumetric expansion of approximately 80%.^[^ ^59^ ^]^ This dramatic change in volume introduces substantial mechanical stress within the cathode matrix, often leading to particle fracture, electrode delamination, and the loss of electrical contact between active materials and the current collector. The repeated expansion, contraction cycles during charge, discharge exacerbate these mechanical degradations, especially in rigid host structures, thus necessitating the development of flexible, robust scaffolds that can buffer such volume changes without compromising structural integrity.

#### 3.2.3 Polysulfide Shuttle Effect and Active Material Loss

During battery discharge, sulfur undergoes multistep electrochemical reduction forming a sequence of lithium polysulfides (Li_2_S_n_, 4 ≤ *n* ≤ 8), many of which are highly soluble in ether‐based electrolytes.^[^ ^4b,c^ ^]^ These LiPS intermediates readily diffuse from the cathode to the lithium anode, where they undergo parasitic redox reactions and form electrically inactive products. This phenomenon, commonly known as the “shuttle effect,” not only causes severe active material loss and low coulombic efficiency but also triggers uncontrolled surface reactions on the lithium metal, destabilizing the solid electrolyte interface (SEI) and compromising the long‐term stability of the cell. The persistent migration of these species also results in self‐discharge and accelerated capacity decay.

#### 3.2.4 Sluggish Redox Kinetics and High Overpotentials

The multi‐electron sulfur redox reactions, particularly the conversion between long‐chain LiPS and insoluble short‐chain Li_2_S/Li_2_S_2_, are kinetically sluggish. The formation and decomposition of Li_2_S, in particular, are associated with high nucleation energy barriers, resulting in significant overpotentials during charge–discharge processes.^[^ ^60^ ^]^ This not only lowers energy efficiency but also leads to asymmetric voltage profiles and degraded rate capability. Catalysts that can lower the activation energy and facilitate the adsorption, conversion, and desorption of intermediates are increasingly recognized as crucial components of high‐performance Li–S battery systems.

#### 3.2.5 Interfacial Instability and Side Reactions

The complex and dynamic chemical environment within Li–S batteries leads to unstable electrode–electrolyte interfaces on both the cathode and anode sides. On the cathode, the deposition of Li_2_S can block active sites and hinder ion transport, while the continuous dissolution of LiPS corrodes the cathode matrix.^[^ ^61^ ^]^ On the anode, the presence of soluble LiPS can penetrate the SEI layer, initiating parasitic reactions and forming electrically inactive products. These processes collectively result in the accumulation of insulating species, loss of active lithium and sulfur, and elevated internal resistance, all contributing to poor long‐term cycling performance.

#### 3.2.6 Lithium Metal Anode Instability and Dendrite Growth

While the Li metal anode offers unparalleled theoretical capacity (3860 mAh g^−1^), it suffers from severe practical limitations, including dendritic lithium growth during repeated deposition/stripping processes.^[^ ^62^ ^]^ These dendrites can penetrate the separator and lead to internal short circuits, posing significant safety risks. Furthermore, the high reactivity of Li with electrolytes and dissolved polysulfides leads to unstable and continuously evolving SEI layers, exacerbating Li loss and increasing cell impedance.^[^ ^63^ ^]^ Mitigating these issues requires protective coatings, electrolyte additives, or the design of artificial SEI layers to regulate Li ions flux and suppress dendrite formation.

The practical deployment of Li–S batteries is severely constrained by a series of interrelated challenges, including the poor electronic conductivity and volumetric instability of sulfur, the dissolution and migration of LiPS, and the dendritic growth and interface instability of Li metal anodes. These problems are intrinsically rooted in complex redox chemistry, multistep phase conversions, and uncontrolled interfacial reactions. Conventional material strategies, such as carbon hosts or binary catalysts, have shown partial improvements but often fail to simultaneously address the multifaceted issues under realistic loading and cycling conditions. Against this backdrop, HEC have emerged as a compelling class of multifunctional materials capable of providing integrated solutions. Owing to their compositional diversity and highly tunable electronic structures, HEC can offer abundant active sites for catalyzing LiPS redox reactions, facilitate uniform Li_2_S nucleation, and suppress shuttle behavior via strong chemisorption. Moreover, the thermodynamic stabilization conferred by high configurational entropy can endow these catalysts with exceptional phase stability under electrochemical cycling. The synergistic interactions among constituent elements allow for the tailoring of binding energies, conductivity, and structural resilience, providing a versatile platform to simultaneously address cathode kinetics, sulfur confinement, and interfacial compatibility.

## 4 Synthetic Strategies for High‐Entropy Catalysts

The synthesis of HEC represents a critical step in realizing their compositional complexity, homogeneous phase formation, and catalytic functionality. To achieve a stable single‐phase solid solution or nanostructured material from multiple principal elements, it is essential to control both the thermodynamics (phase stability) and kinetics (diffusion, nucleation, and grain growth) during synthesis. Various synthetic routes have been explored, including high‐temperature methods, wet‐chemical routes, mechanical alloying, and template‐assisted strategies, each offering unique advantages depending on the target application and structural form.

### 4.1 High‐Temperature Solid‐State Synthesis

**Figure 4:**
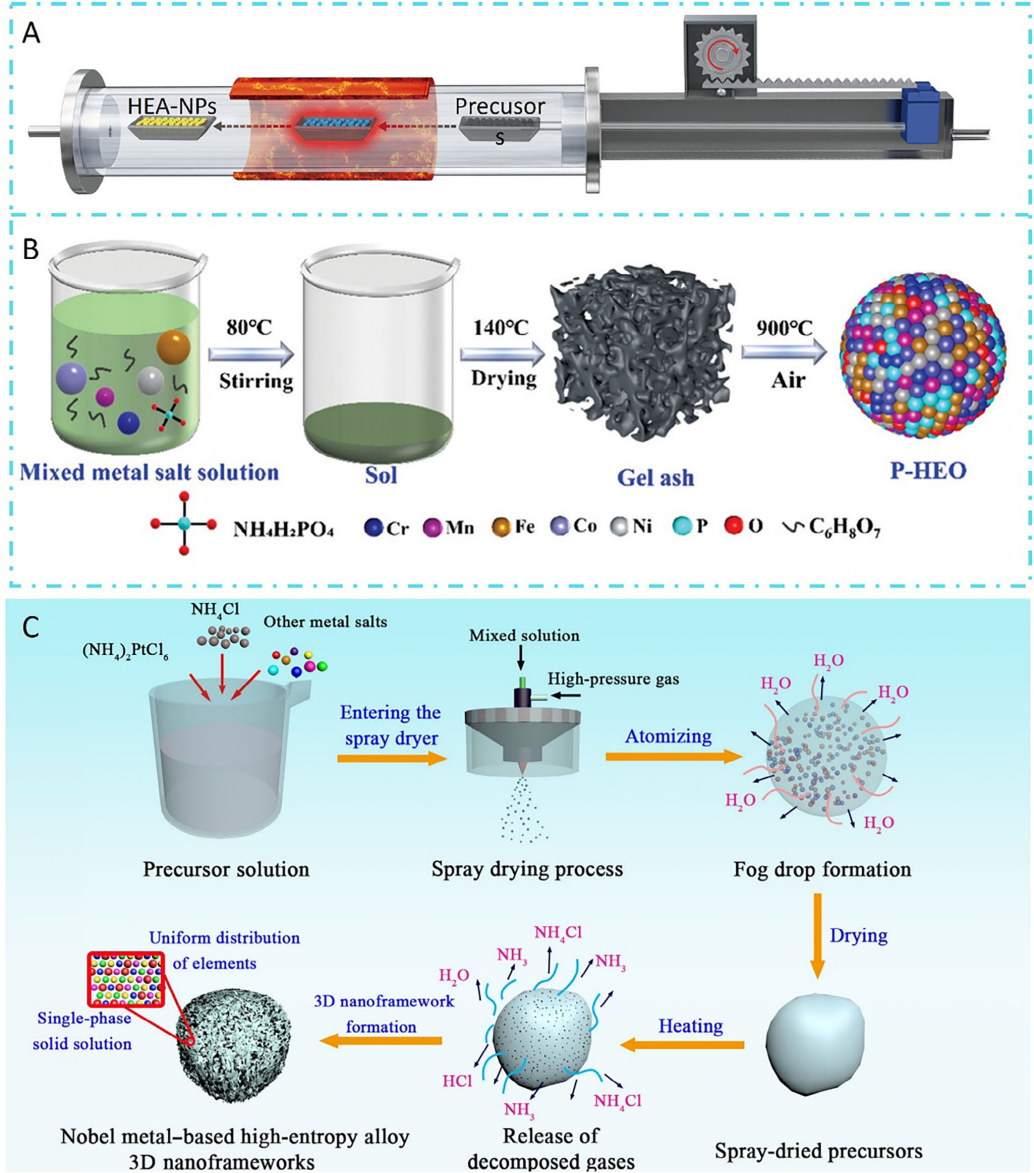
A) Schematic diagram of the preparation method of high‐temperature solid‐phase sintering. Reproduced with permission.^[^ ^37^ ^]^ Copyright 2020, Springer Nature. B) Schematic diagram of the preparation route by sol–gel method. Reproduced with permission.^[^ ^77^ ^]^ Copyright 2024, Wiley‐VCH. C) Schematic diagram of the spray pyrolysis preparation process. Reproduced with permission.^[^ ^78^ ^]^ Copyright 2025, American Association for the Advancement of Science.

The high‐temperature solid‐state synthesis method is a widely adopted and well‐established strategy for preparing high‐entropy catalysts, particularly oxides and alloys. In this approach, stoichiometric mixtures of oxide, carbonate, or metal precursors are thoroughly ground to ensure homogeneity and subsequently calcined at elevated temperatures‐typically ranging from 900 to 1300 °C (**Figure** **4**A).^[^ ^37^ ^]^ The intense thermal energy drives atomic interdiffusion and solid‐state reactions among the precursors, facilitating the formation of single‐phase high‐entropy compounds. One of the primary advantages of this method lies in its simplicity and scalability, as it requires minimal solvent use, avoids complex reaction setups, and is readily applicable to industrial‐scale synthesis. Additionally, the high‐temperature environment ensures thermodynamic equilibrium and promotes crystallinity, often leading to structurally stable and phase‐pure materials. However, the requirement for prolonged high‐temperature treatment may lead to grain coarsening and particle agglomeration, which can reduce surface area—an undesirable feature for heterogeneous catalysis. Furthermore, the kinetics of solid‐state diffusion are relatively slow, making it challenging to obtain homogeneous products without extensive grinding and long reaction times. The lack of precise morphology control and limited porosity are further drawbacks that hinder catalytic performance, particularly in reactions requiring high surface exposure or fast mass transfer (**Table** **1**).

**Table 1:** Summary of the synthetic method and application of high‐entropy catalyst.

High‐entropy catalyst	Method	Structure	Temperature	Application	Refs.
(Mg_0.2_Co_0.2_Ni_0.2_Cu_0.2_Zn_0.2_)Fe_2_O_4_	Solid‐state	Spinel	>1000 °C	OER	[64]
(FeCoNiCrMn)_3_O_4_	Solid‐state	Spinel	1000 °C	Li‐ion	[65]
La(CoCuFeAlCe)_0.2_O_3_	Sol–gel	Perovskite	800 °C	MSR	[66]
La(FeCoNiCrMn)O_3‐x_	Sol–gel	Perovskite	650 °C	Li‐ion	[67]
(FeCuCrMnNi)_3_O_4_	Spray pyrolysis	Spinel	700 °C	Na‐ion	[68]
(ZnNiMnFeTi)_3_O_4_/(CuNiMnFeTi)_3_O_4_	Spray pyrolysis	Spinel	135 °C	Li‐ion	[69]
La_5_CoCr_0.5_NiMnFeO_x_	Co‐precipitation	Perovskite	650 °C	MSR	[70]
(FeNiCoCrMnV)O_x_	Carbon‐thermal shock	Spinel	1300 °C	OER	[71]
FeNiCoCuPt	Carbon‐thermal shock	FCC	800 °C	OER/HER	[72]
CrMnFeCoNi	Plasma‐assisted deposition	FCC	1000 °C	ORR	[73]
FeCoNiMoCr/FeCoNiMoCu	Mechanical alloying	BCC/FCC	Room temperature	OER	[74]
AuAgPtPdCu	Mechanical alloying	FCC	Room temperature	Co_2_RR	[31]
PtFeCoNiMn	MOF‐derived	FCC	1000 °C	ORR/OER	[75]
CoNiCuMnAl	MOF‐derived	FCC	700 °C	OER	[76]

### 4.2 Sol–Gel Synthesis

The sol–gel method is a versatile and widely used wet‐chemical approach for synthesizing high‐entropy catalysts, especially those based on oxides. In this process, metal salts or alkoxides‐usually nitrates or chlorides are dissolved in a suitable solvent system (e.g., water, ethanol) to form a homogeneous solution or sol. Subsequent hydrolysis and polycondensation reactions, often promoted by pH adjustments or chelating agents (e.g., citric acid or ethylenediaminetetraacetic acid), lead to the formation of a three‐dimensional gel network. Upon drying and thermal calcination, this gel transforms into a crystalline HEO (Figure 4B).^[^ ^77^ ^]^ One of the major strengths of the sol–gel method lies in its excellent control over chemical homogeneity at the molecular level, which is crucial for the formation of single‐phase multicomponent systems. The relatively low synthesis temperature compared to solid‐state methods helps to minimize grain growth and particle sintering, yielding nanostructured materials with high surface areas‐an essential feature for catalytic applications. Moreover, the flexible precursor chemistry enables fine‐tuning of morphology, porosity, and even doping levels, making sol–gel synthesis particularly attractive for designing nanostructured high‐entropy catalysts.

Nevertheless, there are inherent drawbacks associated with this technique. The need for multiple organic reagents and solvents can complicate the process, and the risk of incomplete hydrolysis or uneven condensation may lead to compositional inhomogeneities or phase separation. Furthermore, the drying and calcination stages must be carefully optimized, as uncontrolled heating may induce pore collapse or phase instability. Despite these limitations, the sol–gel route remains a powerful method for synthesizing HEO with controlled microstructures and superior catalytic performance.

### 4.3 Spray Pyrolysis

Spray pyrolysis is a continuous and scalable synthesis strategy that has gained considerable attention for the preparation of high‐entropy catalysts, especially in nanoparticle or microsphere forms. In this method, a precursor solution, typically containing soluble metal salts in stoichiometric ratios, is atomized into fine droplets using ultrasonic or pneumatic nebulizers. These droplets are then carried by a carrier gas (e.g., air, nitrogen) into a high‐temperature furnace, where rapid solvent evaporation, solute precipitation, and thermal decomposition occur simultaneously to yield solid particles (Figure 4C).^[^ ^78^ ^]^ A distinct advantage of spray pyrolysis lies in its simplicity and ability to produce homogeneous, spherical, and often hollow particles in a single step. The rapid quenching and high surface area of the resulting powders are beneficial for catalytic applications. Moreover, the method allows for precise control over composition, particle morphology, and porosity through adjustment of precursor concentration, atomization rate, and reaction temperature. It is especially suitable for preparing metastable multicomponent oxides, as the fast processing kinetics can suppress phase segregation and promote the formation of single‐phase high‐entropy materials. Despite these strengths, spray pyrolysis also faces several limitations. The process generally requires relatively high synthesis temperatures (typically above 600 °C), which can lead to particle sintering and reduced surface area if not carefully optimized. Additionally, the formation of uniform solid solution phases is strongly dependent on the volatility and thermal decomposition behavior of each metal precursor, which may not always be compatible. Furthermore, the equipment setup can be more complex than sol–gel or co‐precipitation methods, and the scale‐up, while feasible, requires precise control over aerosol dynamics and thermal profiles.

### 4.4 Co‐Precipitation Method

**Figure 5:**
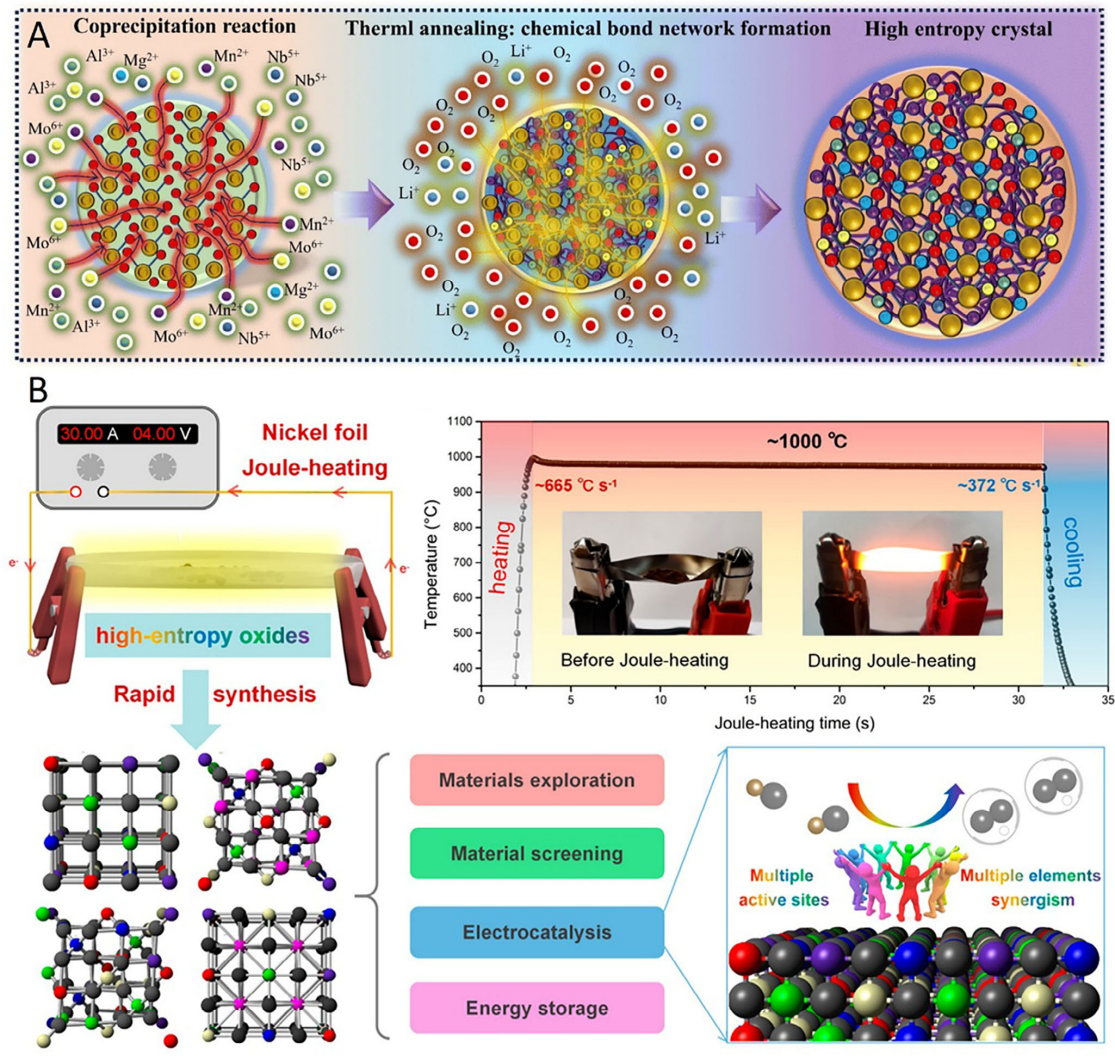
A) The schematic diagram of Co‐precipitation method. Reproduced with permission.^[^ ^79^ ^]^ Copyright 2025, Royal Society of Chemistry. B) The schematic diagram of carbon‐thermal shock synthesis. Reproduced with permission.^[^ ^80^ ^]^ Copyright 2022, American Chemical Society.

The co‐precipitation method is one of the most widely adopted wet‐chemical synthesis strategies for high‐entropy catalysts due to its simplicity, low cost, and compatibility with various multimetallic systems. In this approach, metal salts (usually nitrates or chlorides) are dissolved in an aqueous solution in near‐equimolar ratios and subsequently precipitated together by the addition of a suitable base (e.g., NaOH, NH_4_OH) or precipitating agent (e.g., oxalate, carbonate) (**Figure** **5**A).^[^ ^79^ ^]^ The simultaneous formation of metal hydroxides or precursors ensures a homogeneous distribution of the constituent elements at the molecular level. The obtained precipitate is then washed, dried, and subjected to calcination or reduction to form the final high‐entropy material in oxide, sulfide, or metallic form.

One key advantage of the co‐precipitation method is its excellent compositional tunability and stoichiometric control. It allows for intimate mixing of different metal cations, which helps suppress phase segregation during subsequent thermal treatments and facilitates the formation of single‐phase high‐entropy solid solutions. This method also enables the introduction of specific anions (e.g., phosphate, carbonate) during precipitation to tailor the structure and surface chemistry of the resulting catalyst. However, the solubility products and precipitation kinetics of different metal ions can vary significantly, which may lead to compositional inhomogeneity or the formation of multiple phases. In particular, elements with high or low solubility (e.g., Al^3+^, Cu^2+^) may precipitate at different pH ranges, complicating the synthesis of uniform high‐entropy compositions. Additionally, the method often requires careful pH control, slow addition rates, and thorough mixing to achieve reproducibility, especially when scaled up.

### 4.5 Carbon‐Thermal Shock Synthesis

Carbon‐thermal shock (CTS) synthesis is an emerging high‐temperature, ultrafast method for producing high‐entropy nanomaterials, particularly HEAs and metallic catalysts. Since 2018, when Hu et al. successfully synthesized HEA using this method, the fabrication strategy for multicomponent alloys and composite materials has undergone a significant transformation.^[^ ^81^ ^]^ In this process, a precursor mixture containing metal salts or oxides is rapidly heated, typically to 1500–2000 K within milliseconds, by direct contact with a conductive substrate (such as carbon paper or graphite foil) under an inert or reducing atmosphere. The extremely fast heating and cooling rates (on the order of 10^4^–10^5^ K s^−1^) induce a non‐equilibrium reaction environment, enabling the formation of single‐phase, multielemental nanostructures that might otherwise be inaccessible via conventional thermal treatments (Figure 5B).^[^ ^80^ ^]^ The primary advantage of CTS lies in its ability to bypass thermodynamic constraints and suppress phase segregation. The high‐temperature flash and rapid quenching effectively “freeze” metastable configurations, allowing for the synthesis of uniformly mixed high‐entropy nanoparticles with controlled morphology and crystallinity. Moreover, this method is solvent‐free, scalable, and compatible with a wide range of substrates and compositions. The ultrafast reaction kinetics also minimize grain growth and sintering, resulting in small‐sized particles with high surface area desirable features for heterogeneous catalysis. However, CTS also has several challenges and limitations. First, precise control over reaction parameters (e.g., energy input, reaction duration) is critical, as even small deviations can lead to incomplete reduction, residual oxides, or non‐uniform alloying. Second, because the reaction is extremely rapid, it may not always allow for full atomic‐scale mixing, especially in systems with significantly different melting points or diffusivities. Additionally, equipment requirements (e.g., pulsed current sources, protective gas environments) may hinder widespread adoption, particularly in conventional catalysis labs.

### 4.6 Plasma‐Assisted Deposition

Plasma‐assisted methods offer a distinct advantage by creating an energetic, nonequilibrium environment that facilitates rapid nucleation and the formation of metastable solid solutions with uniformly dispersed multi‐element components. Unlike conventional high‐temperature synthesis techniques, plasma‐assisted deposition (PAD) typically operates at lower substrate temperatures (100–500 °C), which helps suppress grain growth and preserves nanoscale structural features essential for catalytic performance. Moreover, PAD techniques afford precise control over film thickness, composition, and crystallinity, enabling the fabrication of high‐entropy surfaces with tailored stoichiometry.

**Figure 6:**
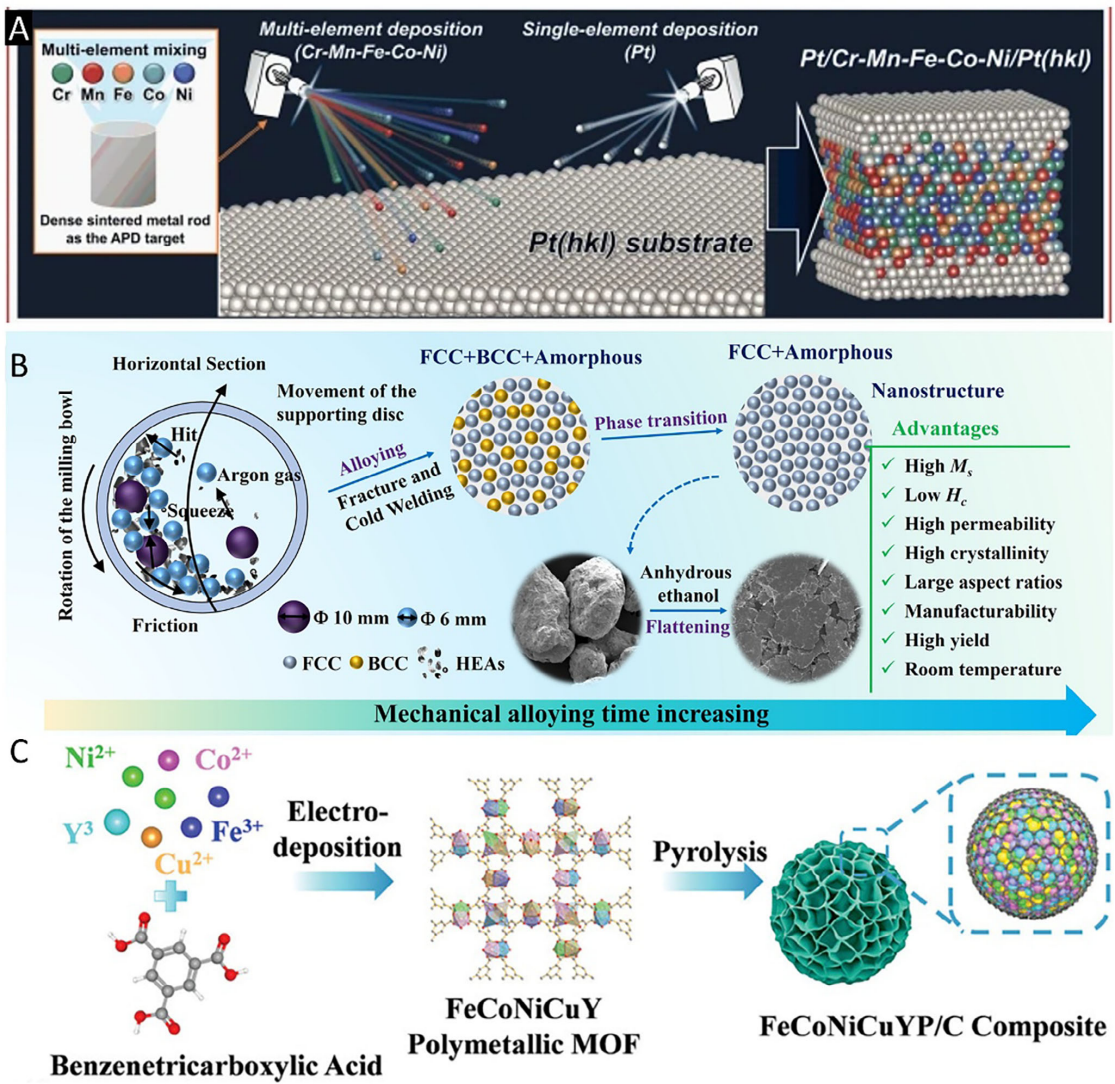
A) Schematic diagram of the plasma‐assisted deposition process. Reproduced with permission.^[^ ^73^ ^]^ Copyright 2023, Springer Nature. B) Schematic diagram of mechanical alloying strategy. Reproduced with permission.^[^ ^82^ ^]^ Copyright 2022, Springer Singapore. C) Schematic diagram of MOF‐derived synthesis. Reproduced with permission.^[^ ^83^ ^]^ Copyright 2024, Wiley‐VCH.

The high kinetic energy of plasma species further enhances the adhesion of the deposited film to the substrate, contributing to improved mechanical and chemical stability under electrochemical or thermal stress. Additionally, PAD is a clean, solvent‐free process that is compatible with a wide range of conductive and insulating substrates. Its scalability for large‐area or patterned fabrication makes it especially attractive for applications in electrodes, sensors, and membrane devices. For instance, Chida et al. employed a plasma‐assisted deposition technique to synthesize an equiatomic CrMnFeCoNi HEA layer on the surface of a single‐crystal Pt substrate, followed by the epitaxial growth of a Pt atomic layer as an ORR catalytic platform. This hierarchical architecture exhibited remarkably high catalytic activity (**Figure** **6**A).^[^ ^73^ ^]^

Nevertheless, achieving uniform and truly random atomic mixing of multiple elements via PAD requires meticulous optimization of sputtering targets or vapor‐phase precursors, particularly when the constituent elements exhibit large disparities in sputtering yields or vapor pressures. Furthermore, the necessity for a vacuum system and high‐energy plasma source increases equipment complexity and cost, which may hinder its broader adoption. Finally, for bulk catalysts or powder‐based applications, PAD alone is insufficient and often needs to be coupled with post‐synthesis treatments to introduce high surface area and porosity.

### 4.7 Mechanical Alloying

Mechanical alloying (MA), a solid‐state powder processing technique, has emerged as a versatile and effective method for the synthesis of high‐entropy catalysts, particularly in systems comprising thermodynamically immiscible elements. This technique involves the repeated fracturing, cold welding, and rewelding of elemental or pre‐alloyed powders within a high‐energy ball mill (Figure 6B).^[^ ^82^ ^]^ Through these processes, atomic‐scale mixing is achieved without requiring the melting of constituents, thereby enabling the formation of metastable solid solutions and nanostructured alloys far from thermodynamic equilibrium.

A significant advantage of mechanical alloying lies in its ability to overcome the inherent limitations of conventional high‐temperature alloying techniques, such as element volatilization, grain coarsening, and undesired phase segregation. The method offers precise control over composition and stoichiometry, is readily scalable for bulk production, and facilitates the formation of nanocrystalline or amorphous phases with tailored structural and electronic properties. Moreover, the solvent‐free and ligand‐free nature of MA renders it environmentally benign and economically viable, making it particularly suitable for large‐scale manufacturing of catalytic materials. From a catalytic standpoint, high‐entropy materials synthesized via mechanical alloying often exhibit enhanced performance, attributed to the high density of crystal defects, dislocations, and grain boundaries introduced during the milling process. These structural irregularities act as abundant active sites, promoting the adsorption and activation of reactant molecules, facilitating charge transfer, and accelerating reaction kinetics. As such, mechanically alloyed high‐entropy catalysts hold great promise for a wide range of electrochemical and thermochemical catalytic applications.

### 4.8 MOF‐Derived Synthesis

Metal–organic frameworks (MOFs), characterized by their highly ordered crystalline architectures, large surface areas, and modular composition, offer an exceptional platform for the rational design and synthesis of high‐entropy catalysts. In MOF‐derived strategies, the MOF acts simultaneously as a structural scaffold and a multimetallic precursor. Upon thermal decomposition, typically under inert or reductive atmospheres, the organic ligands are eliminated while the metal nodes aggregate into catalytically active phases, such as alloyed nanoparticles, mixed metal oxides, or porous carbon‐supported composites. A key advantage of this approach lies in its capacity for atomic‐level metal dispersion and spatially pre‐engineered arrangement of multiple metal ions. The organic linkers in MOFs help stabilize otherwise immiscible metal combinations, facilitating the incorporation of five or more distinct elements within a uniform framework. Subsequent pyrolysis under optimized conditions promotes homogeneous elemental mixing, minimizes phase segregation, and often preserves the inherent porosity of the parent MOF. This transformation results in high‐surface‐area materials with nanoscale grain sizes, abundant and well‐distributed active sites, and accessible porosity, all critical features for catalytic performance. For example, Li et al. employed a plasma‐assisted deposition technique to synthesize an equiatomic CrMnFeCoNi HEA layer on the surface of a single‐crystal Pt substrate, followed by the epitaxial growth of a Pt atomic layer as an ORR catalytic platform. This hierarchical architecture exhibited remarkably high catalytic activity (Figure 6C).^[^ ^83^ ^]^

Another notable strength of MOF‐derived synthesis is its compositional tunability. By judiciously selecting metal‐containing nodes and organic linkers, the local coordination environment and electronic structure of the resulting multimetallic system can be finely tuned. This synthetic flexibility has been successfully harnessed to enhance performance in a range of electrochemical reactions, including the HER, ORR, and carbon dioxide reduction (CO_2_RR). Despite these advantages, several limitations must be addressed. Thermal decomposition can sometimes lead to carbonization or the formation of undesired secondary phases if conditions such as atmosphere composition, heating rate, and precursor stoichiometry are not rigorously controlled. Additionally, the thermal stability of some MOFs is insufficient to accommodate the high‐temperature treatments required for entropy stabilization, thereby narrowing the scope of applicable metal combinations. Achieving homogeneous elemental mixing remains particularly challenging when dealing with metals that possess divergent redox potentials or thermal behaviors.

The synthesis of high‐entropy catalysts encompasses a wide range of methodologies, each offering distinct advantages in terms of elemental distribution, phase formation, and surface property modulation. Conventional solid‐state sintering ensures the formation of thermodynamically stable phases but often requires prolonged high‐temperature treatment and may suffer from compositional inhomogeneity. Solution‐based approaches, such as sol–gel and co‐precipitation, allow for precise stoichiometric control and homogeneous mixing at the molecular level, although challenges remain in achieving phase purity and crystallinity. Advanced techniques like carbothermal shock and plasma‐assisted deposition provide non‐equilibrium conditions conducive to metastable phase formation and surface activation, yet their scalability and reproducibility are still under investigation. Despite the progress made, a comprehensive understanding of the structure–processing–property relationships remains critical. Future advances will likely arise from the integration of in situ/operando characterization, machine learning‐guided synthesis optimization, and multiscale modeling. These combined efforts will be instrumental in tailoring high‐entropy catalysts with controlled architectures and functionalities.

## 5 Application of HEC in Li–S Batteries

HEC have attracted significant attention in the field of electrochemical energy storage and conversion owing to their unconventional physicochemical properties. The inherent compositional complexity of HEC enables a rich distribution of catalytic active sites and allows for the fine‐tuning of surface electronic structures via synergistic multi‐element interactions. Such tuning often leads to optimized adsorption energies for reaction intermediates, effectively enhancing catalytic activity and selectivity. Moreover, the high configurational entropy inherent to these systems promotes the formation of stable solid‐solution phases and suppresses undesirable phase segregation, even under high‐temperature or long‐duration electrochemical cycling. The presence of multiple transition metals in HEC creates diverse coordination environments that not only strengthen the adsorption of LiPS via multi‐site chemisorption, but also facilitate multistep electron‐transfer processes by lowering the energy barriers of intermediate reactions. Furthermore, the sluggish ion diffusion kinetics intrinsic to HEC can effectively impede the outward migration of soluble polysulfide species, thereby suppressing the shuttle effect and improving long‐term cycling performance.

Beyond adsorption and catalytic enhancement, the thermodynamically stable yet kinetically tunable nature of HEC allows them to maintain structural integrity during a complex chemical reaction environment, mitigating capacity fading. Their remarkable mechanical and electrochemical durability is particularly beneficial when used in nanostructured sulfur hosts or functional interlayers, where dimensional and structural collapse is a common failure mode. Thus, by integrating high‐entropy chemistry with rational structural design, these multifunctional materials offer an alternative strategy for mediating complex interfacial reactions and enhancing the practical viability of Li–S batteries. Moreover, **Table** **2** shows the recent advancements in various types of high‐entropy catalysts employed as primary catalytic materials in Li–S battery systems.

**Table 2:** Electrochemical performance of Li–S batteries with HEC.

Material	Structure	Sulfur loading [mg cm^−2^]	E/S [µL mg^−1^]	Initial capacity [mAh g^−1^]	Long cycle performance/Capacity retention rate	Refs.
VCrNbMoZrN_x_	Cubic	1.89	‐	556	404 mAh g^−1^ at 1 C after 500 cycles/72.6%	[84]
TaVMnCrN_x_	Cubic	3	10	956	539 mAh g^−1^ at 1 C after 1000 cycles/56%	[85]
PtCuFeCoNi	Fcc	‐	25	976.4	556.6 mAh g^−1^ at 6 C after 500 cycles/81.5%	[86]
CoNiCuMnMo	Fcc	4.44	‐	713	320.7 mAh g^−1^ at 3 C after 1000 cycles/45%	[87]
FeCoNiCu	Fcc	4.23	‐	823.5	692 mAh g^−1^ at 1 C after 300 cycles/82.6%	[10a]
FeCoNiCuMn	Fcc	4.4	15	740.1	455.6 mAh g^−1^ at 1 C after 960 cycles/51.5%	[88]
CoNiFePdV	Fcc	4.5	5	1321.2	700 mAh g^−1^ at 1 C after 500 cycles/70.1%	[89]
Co_0.29_Ni_0.15_Fe_0.33_Cu_0.16_Ca_3.9_(PO_4_)_3_(OH)	Hexagonal	6.5	‐	927.1	736.6 mAh g^−1^ at 2 C after 500 cycles/77%	[90]
NiCoMnFeCrP	Hexagonal	6.28	‐	1314	510.6 mAh g^−1^ at 3 C after 300 cycles/63%	[52]
NiCoCuTiVSx	Fcc	4.2	5	1120.8	725.4 mAh g^−1^ at 1 C after 230 cycles/63%	[91]
FeCoNiCrMnS_2_	Orthorhombic	5	‐	1098.3	906.8 mAh g^−1^ at 1 C after 1000 cycles/82.5%	[92]
(Mg_0.2_Mn_0.2_Ni_0.2_Co_0.2_Zn_0.2_)Fe_2_O_4_	Spinel	4.6	15	879.6	558.4 mAh g^−1^ at 1 C after 500 cycles/63.5%	[93]
TiVNbMoC_3_	MXene	5.4	8.3	1102.5	877.6 mAh g^−1^ at 1 C after 400 cycles/77.2%	[94]
TiVCrMoC_3_T_x_	MXene	6.4	7.5	1190	553.8 mAh g^−1^ at 2 C after 1000 cycles/78%	[95]
TiS_2_/TiN/TiO_2_/Ti_3_C_2_T_x_	MXene	5.1	10	1091.2	714.6 mAh g^−1^ at 1 C after 200 cycles/71.2%	[96]

### 5.1 High‐Entropy Alloys for Li–S Batteries

High‐entropy alloys (HEA), composed of five or more principal metallic elements in near‐equiatomic ratios, exhibit a single‐phase solid solution stabilized by high configurational entropy. In the context of Li–S batteries, HEA offer an option to overcome several critical limitations such as the sluggish redox kinetics of sulfur species and the LiPS shuttle effect. At the mechanistic level, the compositional complexity of HEA creates a heterogeneous electronic structure and a wide distribution of surface adsorption energies, which enables strong and selective binding with soluble LiPS. This multi‐site adsorption behavior plays a crucial role in immobilizing LiPS intermediates and mitigating their dissolution into the electrolyte. Furthermore, the flexibility in elemental composition allows for the rational design of HEA with tailored catalytic functionalities, such as incorporating transition metals with strong polysulfide affinity or noble metals to improve redox reversibility. Taken together, HEAs serve not merely as structural hosts but also as dynamic electrocatalytic centers that enhance reaction kinetics, stabilize intermediates, and promote long‐term durability. In this section, we present a comprehensive review of the state‐of‐the‐art progress in HEA‐based electrocatalysts for Li–S batteries, elucidating their structure–function relationships and catalytic mechanisms.

**Figure 7:**
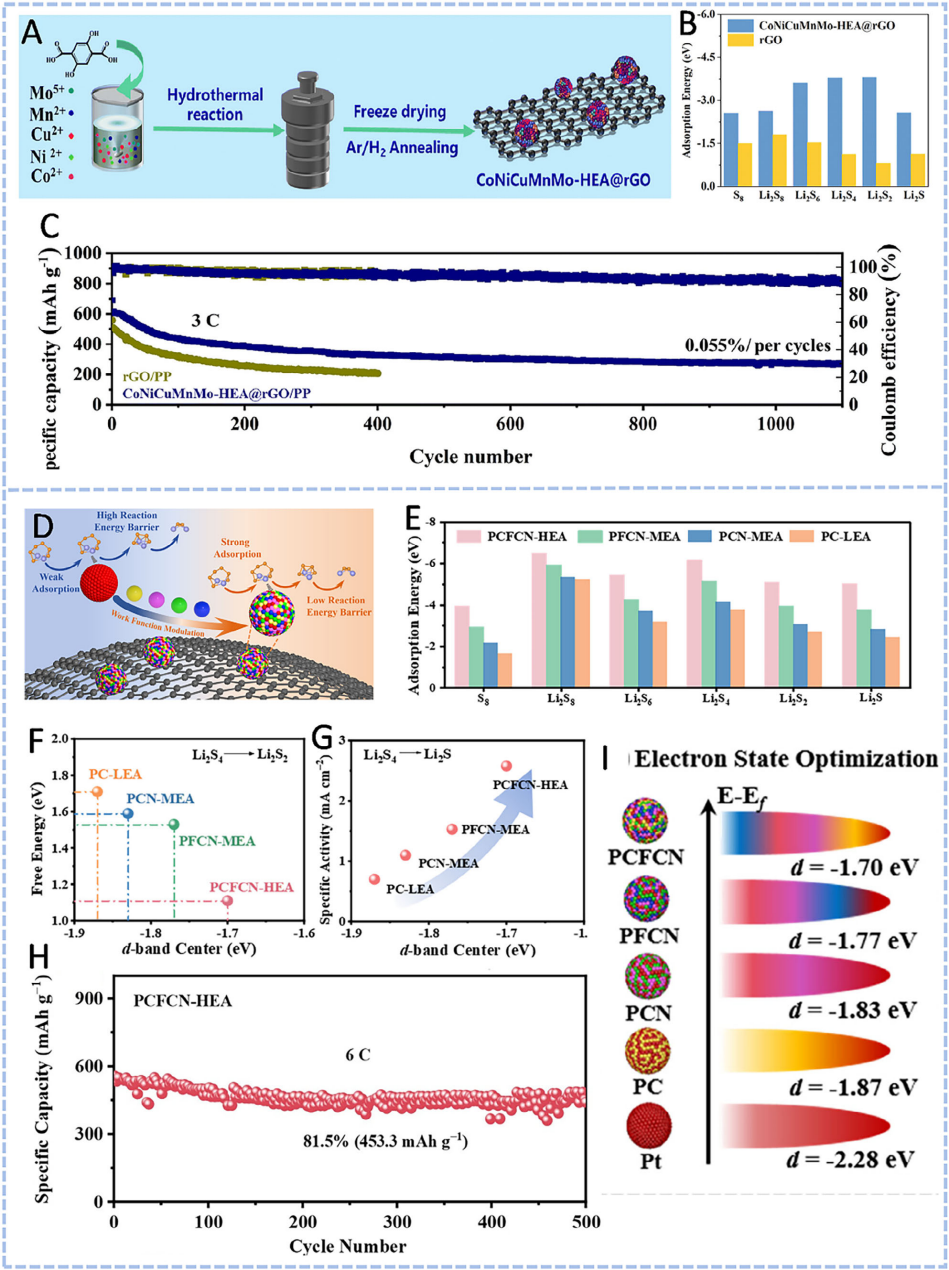
A) Schematic diagram of the synthesis process for a CoNiCuMnMo HEA anchored on reduced graphene oxide sheets (CoNiCuMnMo‐HEA@rGO). B) The binding energies of different S species when adsorbed on HEA@rGO and RGO. C) The corresponding Coulombic efficiency and the cycling performance. Reproduced with permission.^[^ ^87^ ^]^ Copyright 2024, Royal Society of Chemistry. D) Diagrammatic representation of the SRR pathways on PCFCN‐HEA. E) Computed adsorption energies of sulfur species on PC‐LEAs, PCN‐MEAs, PFCN‐MEAs, and PCFCN‐HEAs. F) Theoretical Gibbs free energy for the conversion of Li_2_S_4_ to Li_2_S_2_ as a function of the d‐band center. G) Specific catalytic activity for the conversion of Li_2_S_4_ to Li_2_S in relation to the d‐band center. H) Cycling stability of the PCFCN‐HEA cathode at a rate of 6 C. I) positions of the d‐band center for Pt, PC‐LEAs, PCN‐MEAs, PFCN‐MEAs, and PCFCN‐HEAs. Reproduced with permission.^[^ ^97^ ^]^ Copyright 2025, American Chemical Society.

HEA have garnered significant attention in recent years due to their exceptional performance in catalyzing LiPS conversion on the cathode side of Li–S batteries. For instance, Wang et al. developed an FCC CoNiCuMnMo HEA via a combination of hydrothermal synthesis and high‐temperature pyrolysis. This HEA was anchored ontoreduced graphene oxide (rGO), forming a CoNiCuMnMo‐HEA@rGO composite used as a separator interlayer (**Figure** **7**A). The alloy provides a high density of exposed active sites and demonstrates strong chemical interaction with LiPS (Figure 7B), significantly enhancing redox kinetics. The synergistic effect among the constituent elements leads to charge redistribution and optimized electronic structure, thereby improving active material utilization. Density functional theory (DFT) calculations revealed that this HEA can effectively immobilize LiPS and suppress the shuttle effect by reducing the Fermi level difference between sulfur species and the metallic phase. The resulting CoNiCuMnMo‐HEA@rGO/PP‐based cell maintained a coulombic efficiency above 91.94% for over 1100 cycles at 3C (Figure 7C). ^[^ ^87^ ^]^ In another study, Han et al. introduced a PtCuFeCoNi HEA (PCFCN‐HEA), in which the differences in work function among the five metal elements modulate the d‐band center, thereby enhancing the catalytic behavior. PCFCN‐HEA nanoparticles were loaded onto hollow carbon spheres (HCSs) and integrated with hypha‐like carbon nanobelts (HCNBs), forming a PCFCN‐HEA/HCS/HCNB composite as a sulfur host (Figure 7D–H). The composite exhibits significantly enhanced LiPS adsorption and improved SRR kinetics. DFT analysis indicated that work function disparities drive surface electronic redistribution and induce a d‐band center upshift, which strengthens the orbital hybridization between the metal 3d and sulfur 2p states of LiPS (Figure 7I). This enhances LiPS anchoring and promotes catalytic conversion. The PCFCN‐HEA‐based cathode exhibited superior cycling performance, with a low capacity decay rate of 0.039% per cycle over 1500 cycles at 2 C, and retained high capacity at ultrafast rates up to 8 C.^[^ ^97^ ^]^

**Figure 8:**
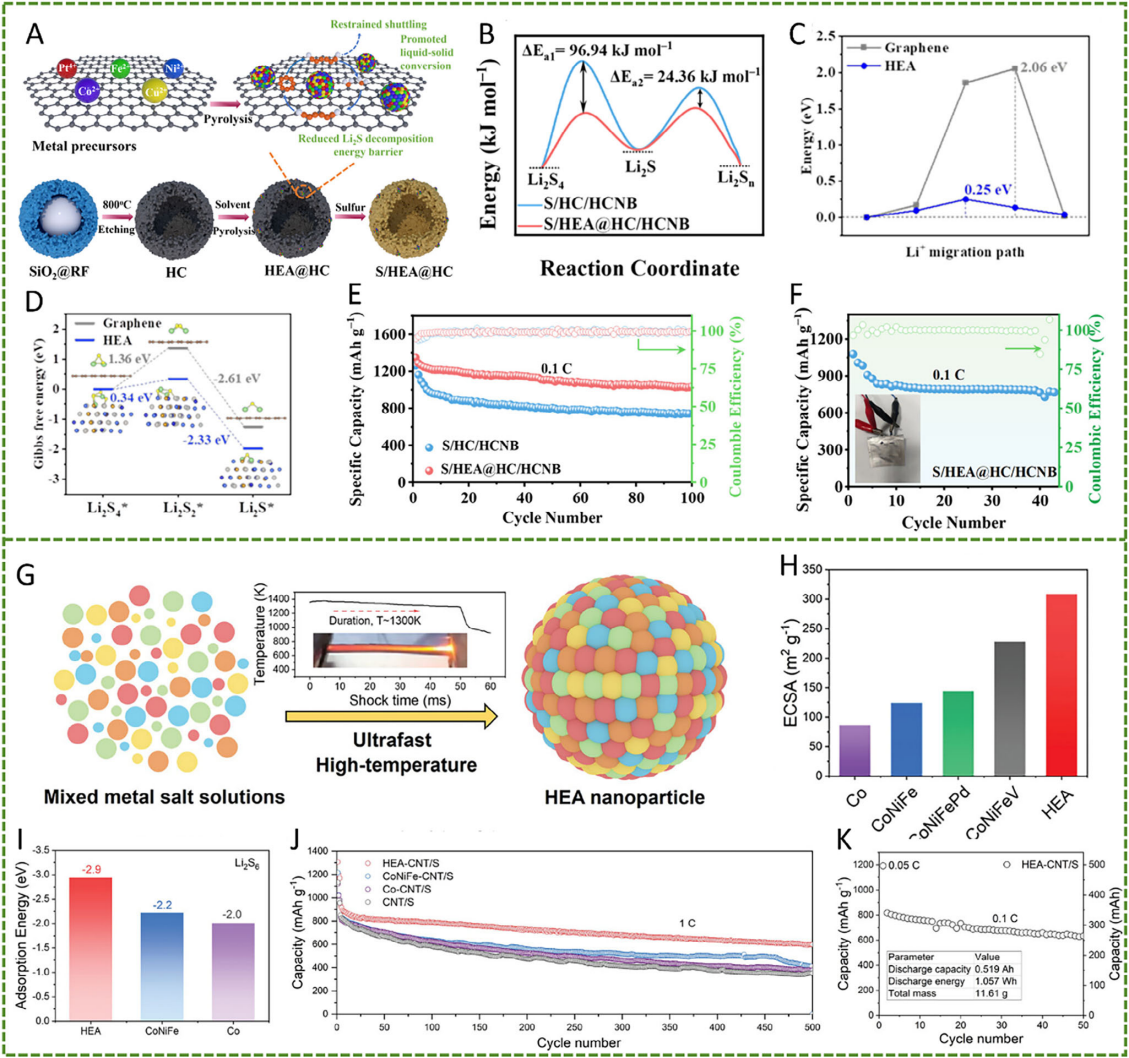
A) Diagram outlining the fabrication process of the S/HEA@HC composite material. B) Comparison of activation energies for the reduction (Li_2_S_4_→Li_2_S) and oxidation (Li_2_S→Li_2_S_n_) reactions. C) Energy barriers for Li_2_S dissociation on HEA versus graphene surfaces. D) Gibbs free energy pathway for the stepwise conversion of Li_2_S_4_→Li_2_S_2_→Li_2_S on a graphene/HEA substrate. E) Electrochemical cycling stability tested at a rate of 0.1 C. F) Long‐term cycling performance of the assembled pouch cell under practical conditions. Reproduced with permission.^[^ ^98^ ^]^ Copyright 2024, American Chemical Society. G) A diagram illustrating the structure and composition of high‐entropy alloys. H) Comparison of ECSA values across various catalytic materials. I) Calculated adsorption energies of lithium polysulfide (Li_2_S_6_) on high‐entropy alloy, CoNiFe, and pure cobalt surfaces. J) Long‐term cycling performance of different sulfur cathodes tested at a 1 C discharge rate. K) Cycling performance evaluation at a low rate (0.1 C) with essential parameters for Li–S pouch cell efficiency. Reproduced with permission.^[^ ^10b^ ^]^ Copyright 2025, Wily‐VCH.

On the other hand, a nanostructured Pt_0.25_Cu_0.25_Fe_0.15_Co_0.15_Ni_0.2_ HEA was synthesized via a simple pyrolysis strategy and anchored onto hollow carbon (HC) structures, forming an HEA/HC composite. This material was further integrated into hypha‐like carbon nanobelts (HCNBs), resulting in a hierarchical S/HEA@HC/HCNB cathode architecture (**Figure** **8**A). Combined computational and experimental results reveal that this HEA exhibits bidirectional catalytic activity toward the stepwise redox conversion of LiPS, thereby accelerating the overall reaction kinetics and mitigating shuttle effects (Figure 8B–D). The corresponding Li–S batteries deliver excellent electrochemical performance, including an initial discharge capacity of 1077.9 mAh g^−1^ at 0.1 C and a capacity retention of 71.3% after 43 cycles in a pouch cell configuration (Figure 8E,F).^[^ ^98^ ^]^ In another study, Xu et al. designed a multi‐element HEA composed of Co, Ni, Fe, Pd, and V to further enhance the catalytic performance of Li–S battery cathodes (Figure 8G). The incorporation of vanadium (V) was found to increase the specific surface area of the HEA nanocatalyst, thereby improving its LiPS adsorption capacity (Figure 8H). Additionally, the synergistic effect among the five constituent metals facilitated multi‐electron and multi‐step sulfur redox reactions, effectively boosting reaction kinetics and stability (Figure 8I). The resulting cathode exhibited a high initial specific capacity of 1364 mAh g^−1^ at 0.1 C and an ultralow capacity decay rate of just 0.054% per cycle over 1000 cycles at 2 C. Notably, the corresponding pouch cell demonstrated a high reversible specific capacity of 1192 mAh g^−1^, highlighting its potential for practical applications (Figure 8J,K).^[^ ^10b^ ^]^

**Figure 9:**
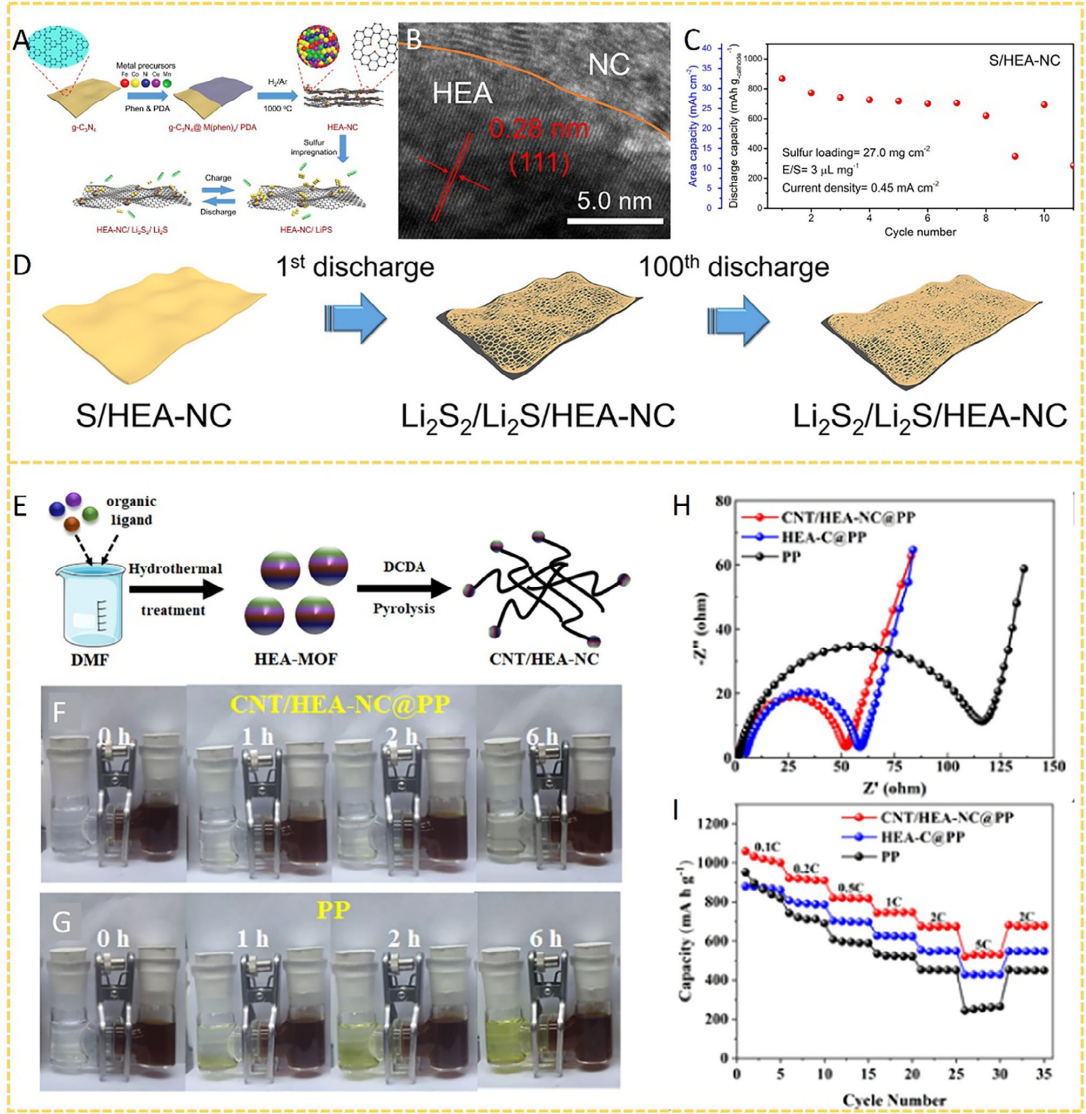
A) Diagram showing the synthesis process of HEA‐NC and its catalytic role in speeding up the conversion between LiPS and Li_2_S_2_/Li_2_S on the HEA–NC host. B) High‐resolution transmission electron microscopy (HRTEM) images of HEA‐NC. C) Electrochemical cycling stability of the S/HEA‐NC cathode under high sulfur loading and low electrolyte conditions. D) Schematic diagrams illustrating the deposition mechanisms at various discharge stages of the S/HEA‐NC electrode. Reproduced with permission.^[^ ^88^ ^]^ Copyright 2022, Wily‐VCH. E) Schematic representation of the carbon nanotube/high‐entropy alloy nanocomposite fabrication procedure. F,G) Barrier performance testing of separators against LiPS migration. H) EIS Nyquist plots. I) Discharge/charge capacity retention under varying current densities for comparative cell configurations. Reproduced with permission.^[^ ^10a^ ^]^ Copyright 2024, Elsevier B.V.

Rational structural design has proven to be a pivotal strategy in enhancing the catalytic activity of HEA by improving their contact with conductive substrates. One representative example involves the introduction of a Fe_0.24_Co_0.26_Ni_0.10_Cu_0.15_Mn_0.25_ HEA as the core catalytic host to boost the electrochemical performance of sulfur cathodes (**Figure** **9**A). In this system, HEA nanocrystallites are uniformly dispersed onto nitrogen‐doped carbon (N‐C), a conductive substrate that not only enhances electron transport but also contributes to chemical adsorption. The HEA nanoparticlesexhibit remarkable electrocatalytic activity toward the SRR, particularly in promoting the solid–solid conversion from Li_2_S_2_ to Li_2_S (Figure 9B–D). The N‐doped carbon, acting as a Lewis base, further improves the chemical affinity toward LiPS, enhancing their anchoring and conversion. The synergistic effect between the catalytic HEA and the polar N‐C host results in a highly efficient cathode, delivering a reversible specific capacity of 1079.5 mAh g^−1^, corresponding to a sulfur utilization rate of 89.4%. Remarkably, under harsh operating conditions, including lean electrolyte (3 µL mg^−1^) and ultrahigh sulfur loading (27.0 mg cm^−2^), the cathode still achieves an areal capacity of 32.4 mAh cm^−2^ (868.2 mAh g^−1^), demonstrating its practical applicability.^[^ ^88^ ^]^ In a related study, Ma et al. developed a highly efficient separator‐modified electrocatalyst comprising HEA nanoparticles embedded in nitrogen‐doped carbon (NC) supported by a conductive carbon nanotube (CNT) framework, denoted as CNT/HEA‐NC (Figure 9E). The HEA nanoparticles catalyze the bidirectional transformation of LiPS, while the polar NC matrix with its porous architecture and strong sulfiphilic nature effectively inhibits the shuttle effect through strong LiPS adsorption (Figure 9F,G). The CNT network ensures rapid ion/electron transport and minimizes internal resistance, further boosting the overall cell kinetics (Figure 9H). When employed as a catalytic interlayer, the CNT/HEA‐NC material imparts the battery with an ultralow capacity decay rate of just 0.03% per cycle and retains a high specific capacity of 521.1 mAh g^−1^ at 5 C (Figure 9I). Notably, even under a high sulfur loading of 4.23 mg cm^−2^, the battery achieves a stable areal capacity of 3.4 mAh cm^−2^ after 120 cycles.^[^ ^10a^ ^]^

**Figure 10:**
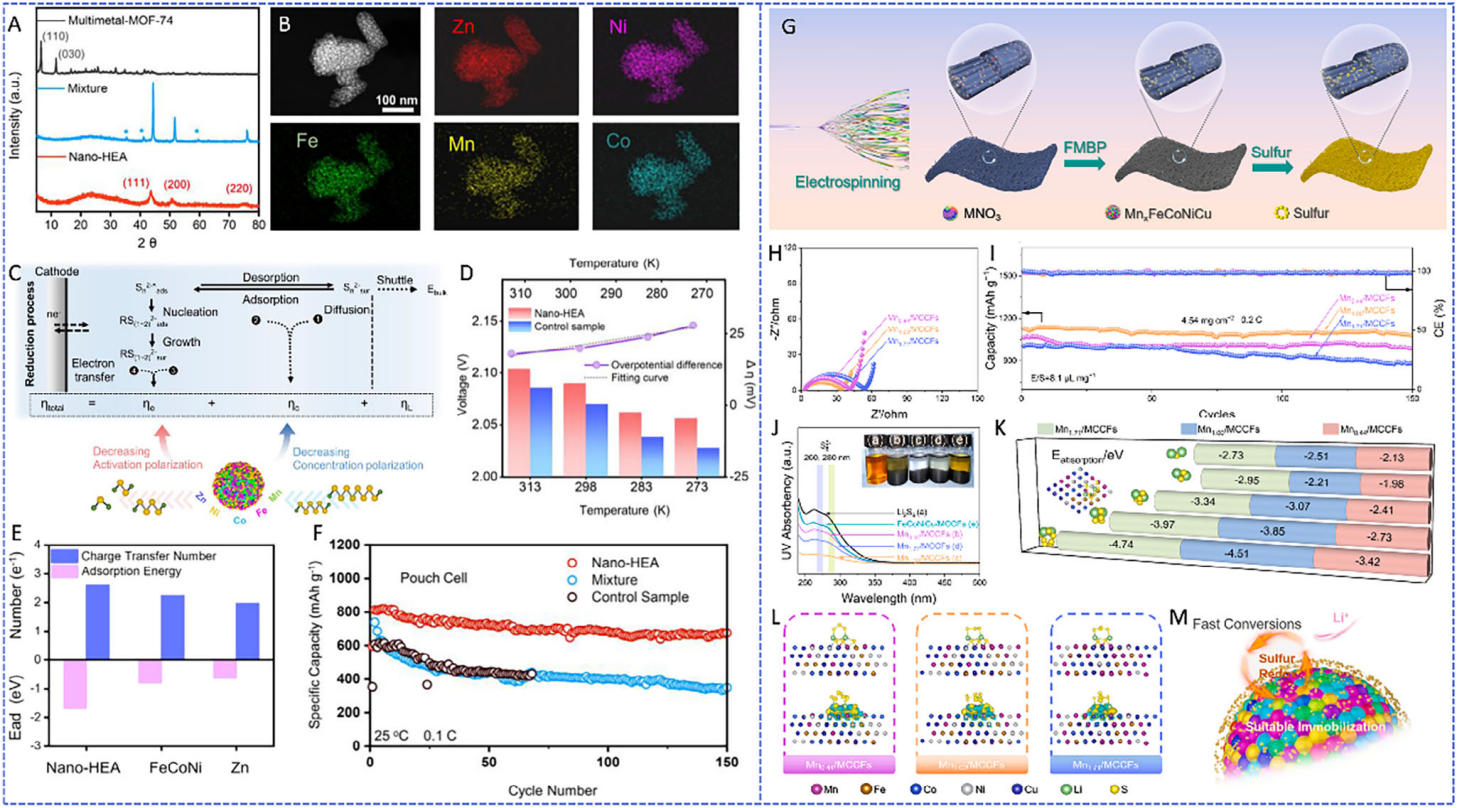
A) XRD pattern of FeCoNiMnZn nano‐HEA catalyst. B) HADDF images of FeCoNiMnZn nano‐HEA catalyst and corresponding elemental mapping. C) Schematic diagram of the catalytic mechanism of FeCoNiMnZn nano‐HEA catalyst in Li–S battery system. D) Comparison of cathode overpotential with and without FeCoNiMnZn nano‐HEA catalyst added. E) Comparison of binding energy and transfer charge number between different catalytic active substrates and Li_2_S_6._ F) Cycling performance of Li–S pouch batteries based on FeCoNiMnZn nano‐HEA catalyst assembly. Reproduced with permission.^[^ ^46^ ^]^ Copyright 2021, Elsevier B.V. G) Schematic diagram of the preparation process of Mn_x_FeCoNiCu/MCCFs (x = 0.44, 1.00, 1.71) samples. H) Nyquist plots of the Li–S cells with Mn_x_FeCoNiCu/MCCFs/S based cathodes. I) Cycling performance of the Mn_x_FeCoNiCu/MCCFs/S based cathodes at a sulfur loading of 4.54 mg cm^−2^. J) UV–vis spectra of Li_2_S_6_ solutions with different active substrates. K) Binding energies of different sulfur species adsorbed on the surface of Mn_x_FeCoNiCu/MCCFs substrates. L) The charge difference density of Li_2_S_6_ on the surface of Mn_x_FeCoNiCu/MCCFs substrates. M) Schematic diagram of the catalytic mechanism of Mn_x_FeCoNiCu/MCCFs catalyst improving sulfur redox reaction. Reproduced with permission.^[^ ^11b^ ^]^ Copyright 2024, Elsevier B.V.

Furthermore, Li et al. synthesized a FeCoNiMnZn high‐entropy alloy (nano‐HEA) with an FCC structure using a carbon thermal shock method based on a MOF‐derived framework, for application as a catalyst to enhance the sulfur redox reaction (**Figure** **10**A,B). The nano‐HEA enhances the redox activity of LiPS, thereby reducing both concentration and activation polarizations during electrochemical cycling and accelerating the conversion kinetics of sulfur species (Figure 10C). Consequently, Li–S batteries incorporating nano‐HEA exhibit a lower Tafel slope and reduced reaction overpotential (Figure 10D). DFT simulations further reveal that nano‐HEA formsstronger chemical interactions with LiPS, which facilitates multi‐electron redox reactions (Figure 10E). Owing to these advantages, Li–S pouch cells incorporating nano‐HEA demonstrate excellent cycling stability (Figure 10F).^[^ ^46^ ^]^ Besides, Lei et al. fabricated Mn_x_FeCoNiCu HEA nanoparticle embedded carbon fiber composites (Mn_x_FeCoNiCu/MCCFs, x = 0.44, 1.00, 1.71) with tunable Mn content via electrospinning (Figure 10G). Owing to the Mn‐induced modulation of the surface electronic structure, the Mn_1.00_FeCoNiCu/MCCFs catalyst facilitates charge mediation and demonstrates enhanced electrochemical performance (Figure 10H,I). All Mn‐containing catalysts demonstrated strong chemical adsorption toward LiPS, which is essential for mitigating the shuttle effect and facilitating polysulfide conversion (Figure 10J,K). DFT calculations further revealed that Mn atoms play a critical role in tuning the electronic environment of the Mn_x_FeCoNiCu/MCCFs. Specifically, Mn sites act as electron‐depleting centers, while the remaining metal atoms serve as electron‐rich regions and primary adsorption sites (Figure 10L,M). This electron redistribution results in an internal electron transfer compensation mechanism that optimizes both adsorption energy and catalytic activity toward LiPS. As a result of these synergistic structural and electronic effects, Li–S pouch cells assembled with Mn_1.00_FeCoNiCu/MCCFs exhibit markedly improved electrochemical performance, including reduced polarization, enhanced capacity retention, and excellent cycling stability.^[^ ^11b^ ^]^

Overall, the integration of high‐entropy alloys into Li–S battery systems has proven to be a compelling strategy for addressing key electrochemical challenges such as LiPS shuttle, sluggish redox kinetics, and poor structural durability. Through their unique compositional complexity and tunable active sites, HEA offer enhanced LiPS immobilization and catalytic conversion capabilities, contributing to improved sulfur utilization and extended cycling life. These findings underscore the effectiveness of entropy‐driven design in advancing sulfur cathode chemistry. Building on the promising results of HEA, attention is increasingly shifting toward other high‐entropy material systems that may further exploit compositional diversity to optimize interfacial behavior and catalytic dynamics in Li–S batteries.

### 5.2 High‐Entropy Metal Oxides for Li–S Batteries

HEO have attracted considerable attention as a new class of electrocatalysts due to their multi‐elemental composition, entropy‐stabilized single‐phase structures, and defect‐rich frameworks. By incorporating five or more metal cations into a homogeneous oxide lattice, HEO exhibit unique physicochemical properties that are inaccessible to conventional binary or ternary oxides. Specifically, the synergistic interaction among diverse metal species in HEO gives rise to a tunable electronic environment, enhanced redox flexibility, and abundant active sites, all of which are highly beneficial for promoting LiPS adsorption and accelerating redox reactions. Moreover, the thermodynamic stability imparted by high configurational entropy facilitates the formation of metastable structures with high surface areas and catalytic activity.

**Figure 11:**
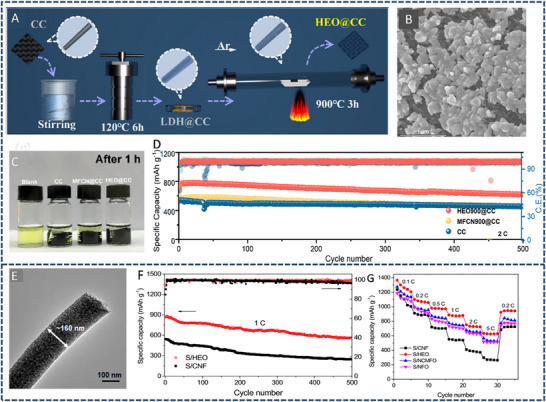
A) Schematic diagram of the preparation principle of HEO@CC. B) The SEM image of HEO@CC. C) Optical photos of LiPS adsorption experiments based on different samples. D) Long cycle performance of Li–S cell based on HEO@CC interlayer. Reproduced with permission.^[^ ^99^ ^]^ Copyright 2025, American Chemical Society. E) The TEM image of (Mg_0.2_Mn_0.2_Co_0.2_Ni_0.2_Zn_0.2_)Fe_2_O_4_ nanofiber. F,G) Long cycle performance and rate performance of Li–S batteries based on HEO nanofibers. Reproduced with permission.^[^ ^93^ ^]^ Copyright 2021, Wiley‐VCH.

For instance, Ji et al. designed a novel HEO heterojunction catalyst by integrating uniformly distributed MnCrO_x_ and FeCoNiO_x_ heterojunction nanoparticles onto a carbon cloth (CC) substrate through a facile hydrothermal synthesis followed by thermal treatment (**Figure** **11**A,B). This multicomponent architecture combines the benefits of heterojunction interfaces and entropy‐stabilized multimetallic oxides, offering abundant chemically active sites for polysulfide immobilization and redox catalysis (Figure 11C). The synergistic interaction at the heterojunction interfaces promotes bidirectional catalytic conversion of LiPS, thereby effectively suppressing the shuttle effect and enhancing redox kinetics. When employed as the cathode host in a Li–S battery, the resulting HEO@CC electrode delivers excellent cycling stability, with an ultralow capacity fading rate of only 0.029% per cycle over 500 cycles at 2 C, alongside commendable rate performance (Figure 11D).^[^ ^99^ ^]^ Tian et al. developed a spinel‐structured ((Mg_0.2_Mn_0.2_Co_0.2_Ni_0.2_Zn_0.2_)Fe_2_O_4_) HEO nanofiber cathode material via an electrospinning strategy, followed by controlled calcination (Figure 11E). The as‐obtained HEO nanofibers, composed of multiple transition metal cations homogenously distributed within a single‐phase spinel matrix, provide a structurally integrated and chemically robust framework for efficient Li–S battery operation. The multi‐site synergistic interactions among the various metal cations significantly enhance the redox kinetics of sulfur species and effectively suppress the LiPS shuttle by promoting strong chemical anchoring of LiPS. Moreover, the polar metal‐oxygen bonds in the HEO lattice facilitate bidirectional catalysis, particularly boosting both the nucleation and decomposition of Li_2_S during cycling. Electrochemical testing revealed that the S/HEO composite cathode delivered a high specific capacity of 632.1 mAh g^−1^even under a stringent current density of 5 C, indicative of excellent rate capability. Additionally, the material exhibited remarkable long‐term cycling stability, maintaining high capacity retention with minimal fading over extended cycles at 1 C (Figure 11F,G).^[^ ^93^ ^]^

**Figure 12:**
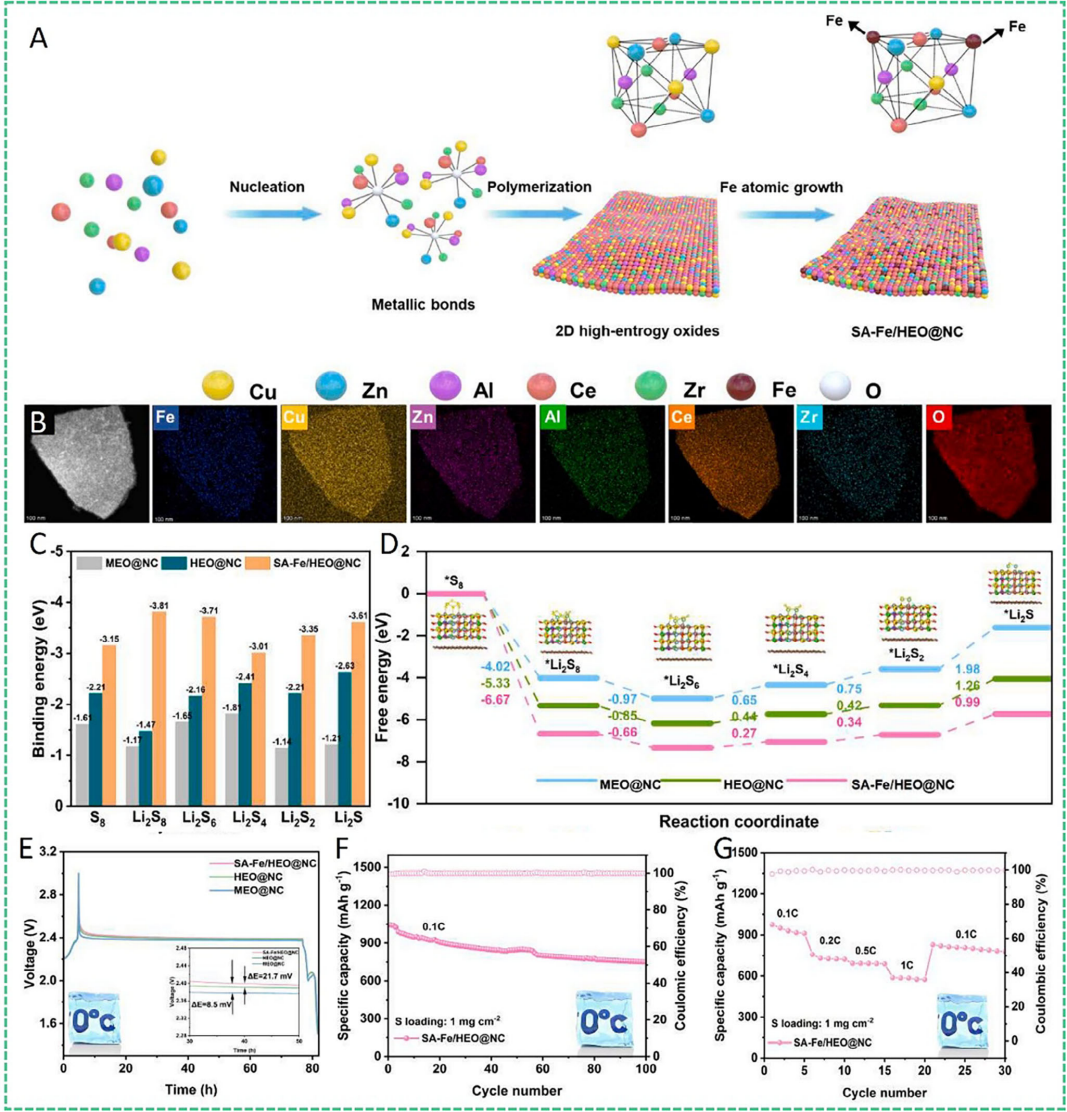
A) Schematic diagram of the synthesis route of SA‐Fe/HEO@NC. B) SEM images of SA‐Fe/HEO@NC and corresponding EDS element mapping. C) Binding energy of LiPS on different active surfaces. D) Gibbs free profiles curves of sulfur species on different active surfaces. E) Voltage‐time curves of various Li–S cells at 0 °C for 72 h. F) Cyclic stability of the SA‐Fe/HEO@NC‐modified Li–S cell evaluated at 0.1 C under low‐temperature conditions (0 °C). G) Rate capability of the SA‐Fe/HEO@NC‐modified Li–S cell tested at various current densities under a subzero environment (0 °C). Reproduced with permission.^[^ ^100^ ^]^ Copyright 2025, Elsevier B.V.

Notably, Na et al. developed a single‐atom iron‐doped HEO catalyst (SA‐Fe/HEO@NC), wherein isolated Fe atoms were embedded into a Cu–Zn–Al–Ce–ZrO‐based HEO matrix and supported on nitrogen‐doped carbon (**Figure** **12**A,B). This unique single‐atom architecture effectively modulated the local electronic structure of the multicomponent active sites, thereby optimizing the adsorption energy landscape for LiPS and enhancing catalytic efficiency (Figure 12C). In particular, the introduction of Fe single atoms was found to facilitate the desolvation of Li ions at the electrolyte‐electrode interface, which is essential for rapid ion transport. Moreover, DFT calculations revealed that SA‐Fe/HEO@NC exhibited the lowest energy barrier for the rate‐determining step of the sulfur redox process among the compared catalysts (Figure 12D), thus enabling efficient acceleration of the liquid‐to‐solid phase conversion kinetics. Electrochemical tests demonstrated that Li–S batteries employing SA‐Fe/HEO@NC as a functional catalyst exhibited both high rate capability and robust cycling performance, even under sub‐ambient conditions (0 °C), highlighting the structural and kinetic resilience of the catalyst in low‐temperature environments (Figure 12E–G).^[^ ^100^ ^]^

**Figure 13:**
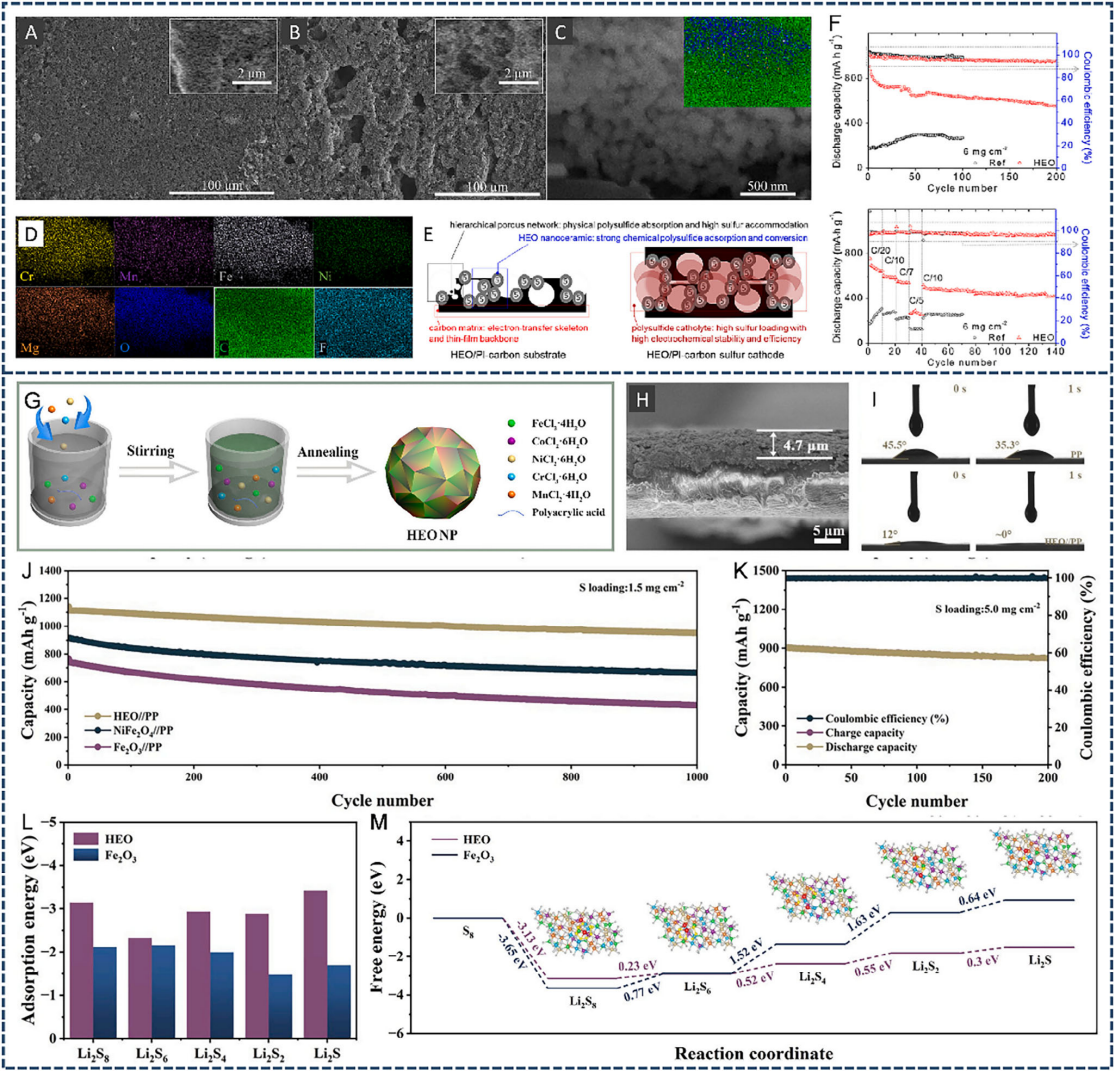
SEM images of the A) compact side and B,C) hierarchical porous side of HEO/PI substrate and D) corresponding SEM element mapping. E) Schematic diagram of HEO/PI substrate. F) Cycling performance and rate performance of HEO/PI‐based sulfur cathode. Reproduced with permission.^[^ ^101^ ^]^ Copyright 2023, Elsevier B.V. G) Schematic diagram of the synthesis route of (FeCoNiCrMn)_3_O_4_ nanoparticles. (H) The cross‐section SEM image of HEO//PP. I) Electrolyte contact angle measurements on different separators. J) Long cycle performance of Li–S batteries assembled with different separators at 1 C current density. K) The cyclic performance of Li–S cell with HEO//PP under the sulfur loading of 5 mg cm^−2^. Reproduced with permission.^[^ ^102^ ^]^ Copyright 2025, Elsevier B.V.

Interestingly, Tseng et al. synthesized a spinel‐structured HEO ((CrMnFeNiMg)_3_O_4_) via a hydrothermal strategy, which was subsequently integrated onto a porous carbon scaffold derived from a phase transition process to form the HEO/PI composite membrane (**Figure** **13**A–D). The incorporation of the HEO endows the system with pronounced chemical polarity, enabling strong chemisorption of LiPS and facilitating their efficient catalytic conversion during electrochemical cycling (Figure 13E). Moreover, the HEO/PI membrane exhibits excellent electrochemical stability and ionic conductivity, which synergistically promote the redox kinetics of sulfur species. Remarkably, even under higher sulfur areal loading conditions (6 mg cm^−2^), the assembled Li–S cell demonstrates robust cycling stability, retaining 62% of its initial capacity after 200 cycles. Additionally, the electrode delivers superior rate performance, underscoring the potential of spinel‐type HEO as multifunctional catalytic hosts for practical high‐loading Li–S battery systems (Figure 13F).^[^ ^101^ ^]^ Similarly, Hao et al. prepared HEO nanoparticles with a spinel structure ((FeCoNiCrMn)_3_O_4_) via a facilethermal evaporation method and applied them as a functional modification layer on the PP separator to enhance the electrochemical performance of Li–S batteries (Figure 13G). The resulting HEO//PP composite separator exhibits excellent electrolyte wettability, which facilitates uniform electrolyte distribution and improves ionic conductivity across the separator interface (Figure 13H,I). When assembled into a full cell, the Li–S battery incorporating the HEO//PP separator retains a high reversible capacity of 951.5 mAh g^−1^ after 1000 charge–discharge cycles, indicating outstanding cycling stability (Figure 13J). Furthermore, the device maintains commendable capacity output even under relatively high sulfur areal loading (5 mg cm^−2^) and moderate current density (0.2 C), demonstrating its suitability for practical applications (Figure 13K). The superior performance can be attributed to the synergistic interactions among the multiple transition metal cations in the HEO matrix, which significantly enhance the chemisorption of LiPS and promote their stepwise conversion (Figure 13L). DFT calculations further support these findings by revealing that the conversion reactions of sulfur species proceed with markedly reduced energy barriers on the HEO surface, thereby accelerating the redox kinetics and mitigating the shuttle effect (Figure 13M).^[^ ^102^ ^]^

**Figure 14:**
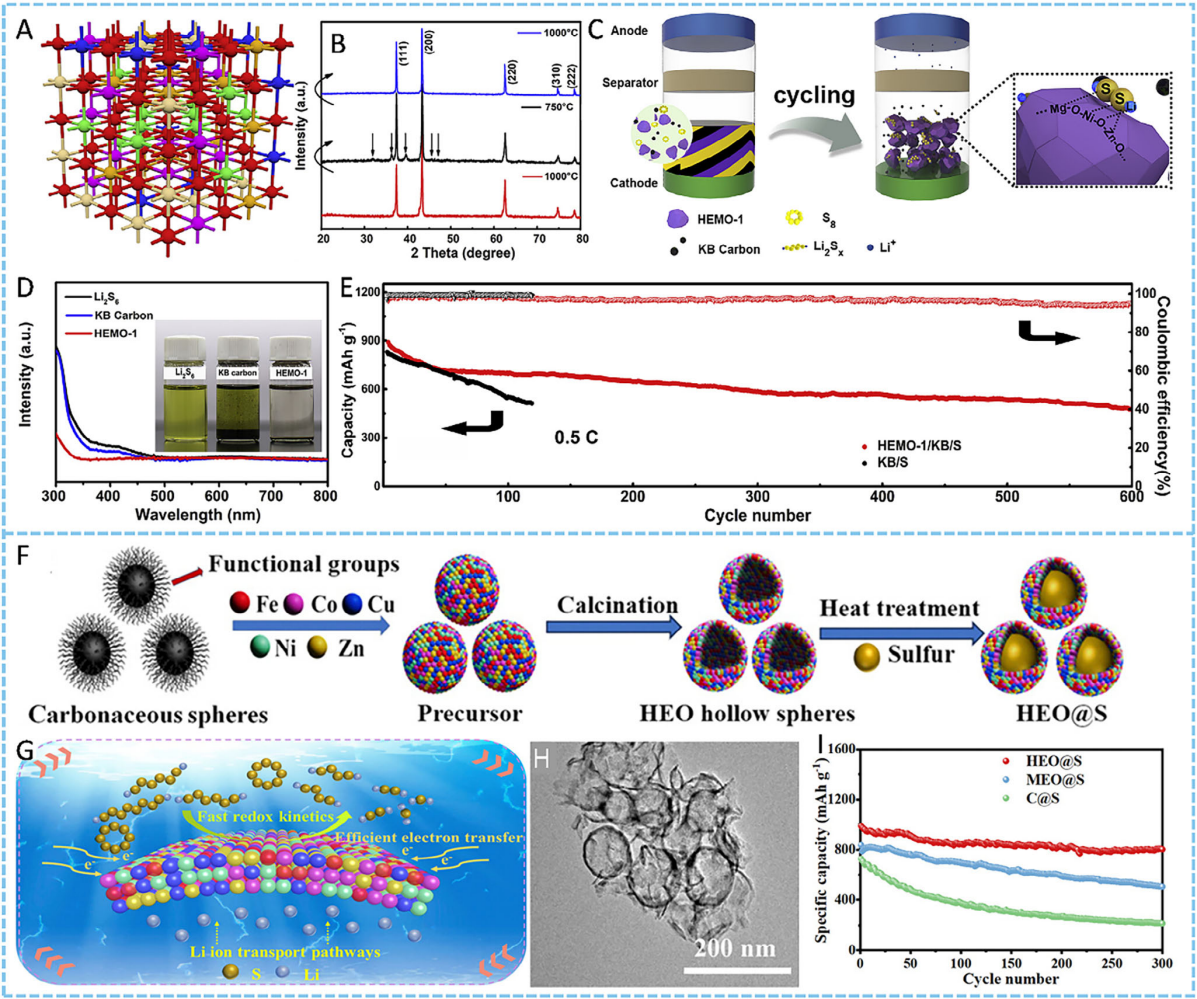
A) Crystal structure and B) XRD pattern of HEMO‐1. C) Schematic diagram of chemical anchoring of LiPS by HEMO‐1 in Li–S battery system. D) UV/visible absorption spectrum of Li_2_S_6_ solution after HEMO‐1 adsorption. E) Cycling performance of HEMO‐1/KB/S cathode at 0.5 C current density. Reproduced with permission.^[^ ^103^ ^]^ Copyright 2019, Elsevier B.V. F) Schematic diagram of the preparation of (Fe_0.2_Co_0.2_Ni_0.2_Cu_0.2_Zn_0.2_)_3_O_4_ hollow spheres. G) Schematic diagram of the advantages of (Fe_0.2_Co_0.2_Ni_0.2_Cu_0.2_Zn_0.2_)_3_O_4_ as a catalyst in Li–S batteries. H) TEM image of (Fe_0.2_Co_0.2_Ni_0.2_Cu_0.2_Zn_0.2_)_3_O_4_. I) Electrochemical cycling performance of the HEO@S cathode at 0.5 C. Reproduced with permission.^[^ ^104^ ^]^ Copyright 2025, Royal Society of Chemistry.

Furthermore, Zheng et al. synthesized a high‐entropy metal oxide (HEMO‐1) with a rock‐salt crystal structure by employing a mechanochemical‐assisted strategy, wherein five metal elements (Ni, Mg, Cu, Zn, and Co) were uniformly incorporated into a single oxide lattice (HEMO‐1, **Figure** **14**A,B). The homogeneous distribution of multiple metal cations within the crystalline matrix endows HEMO‐1 with a diversity of catalytically active sites, each capable of interacting with LiPS through strong chemical bonding with sulfur atoms (Figure 14C). This effective immobilization of LiPS was visually confirmed by adsorption tests, indicating the strong LiPS affinity of the material (Figure 14D). In addition to its anchoring function, the chemically active surface of HEMO‐1 facilitates the reversible redox reactions of sulfur species, contributing to enhanced conversion kinetics throughout the discharge‐charge process. When applied in a Li–S battery system, the HEMO‐1‐based cathode demonstrates excellent cycling stability, underscoring its potential as a robust and multifunctional catalytic host for suppressing shuttle effects and accelerating sulfur electrochemistry (Figure 14E).^[^ ^103^ ^]^ Yu et al. designed and synthesized a HEO catalyst with the nominal composition of ((Fe_0.2_Co_0.2_Ni_0.2_Cu_0.2_Zn_0.2_)_3_O_4_), employing a carbon sphere template method to obtain a well‐defined hollow spherical architecture (Figure 14F). This unique hollow morphology significantly increases the specific surface area and facilitates the exposure of more catalytically active sites, while simultaneously shortening the lithium‐ion diffusion pathways. As a result, the HEO catalyst not only restricts the shuttle of LiPS through a combination of physical confinement and chemical anchoring but also effectively accelerates the redox kinetics of sulfur species during cycling (Figure 14G,H). Electrochemical evaluation reveals that the HEO@S composite cathode exhibits excellent rate performance and prolonged cycling stability, delivering a high specific capacity of 802 mAh g^−1^ after 300 cycles at a current density of 0.5 C (Figure 14I). These results highlight the beneficial interplay between rational structural design and compositional complexity in boosting sulfur utilization and electrochemical durability in Li–S batteries.^[^ ^104^ ^]^

**Figure 15:**
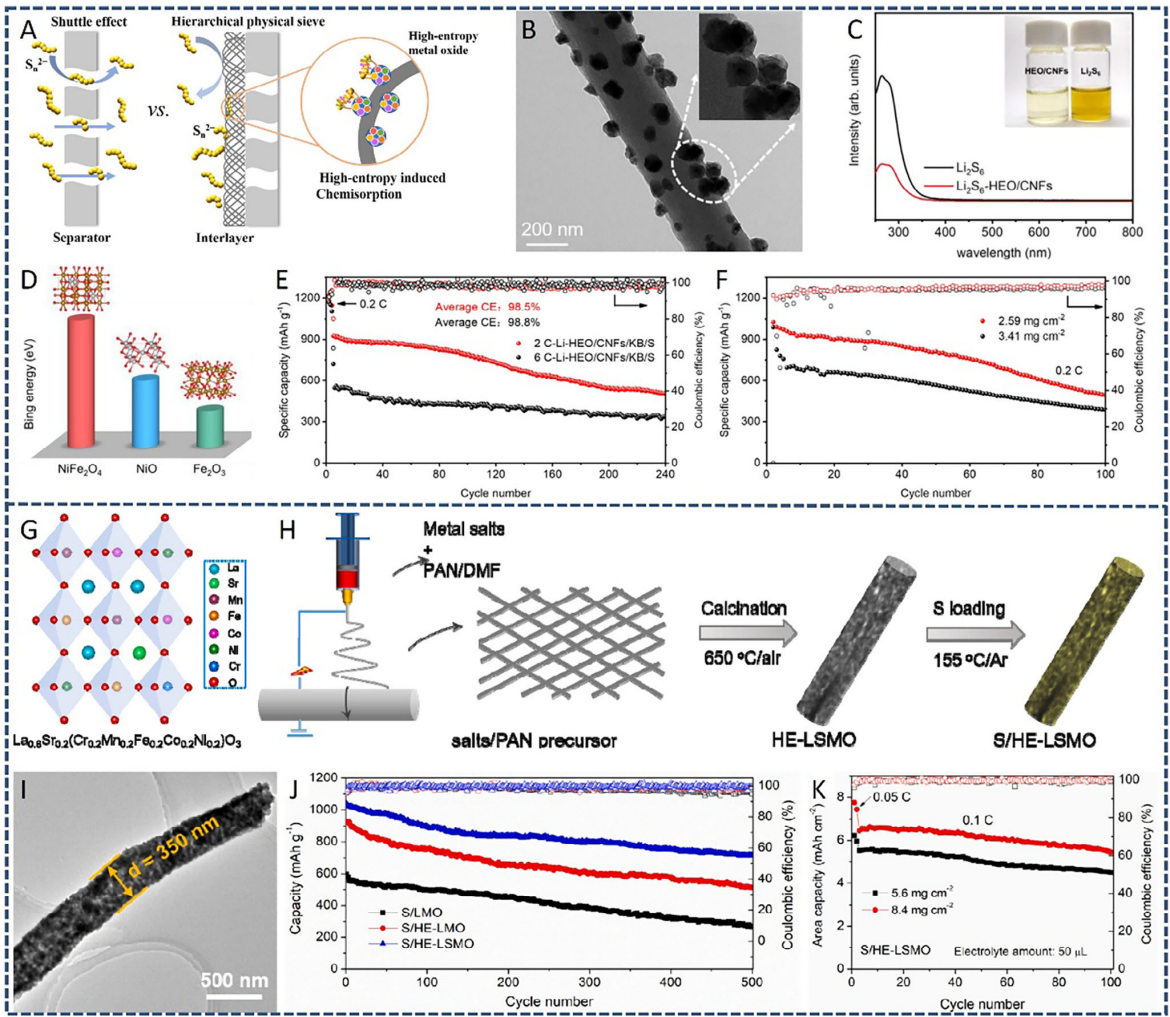
A) Schematic diagram of the action mechanism of HEO/CNFs in Li–S batteries. B) SEM image of HEO/CNFs, C) UV–visible absorption spectrum of Li_2_S_6_ solution after HEO/CNFs adsorption. D) Comparison of binding energy of Li_2_S_6_ on different active surfaces. E) Cycle stability of HEO/CNFs‐based cathode under 2 and 6 C current densities. F) Cycle stability of HEO/CNFs‐based cathode under higher sulfur loading. Reproduced with permission.^[^ ^105^ ^]^ Copyright 2022, Elsevier B.V. G) Crystal structure of the synthesized La_0.8_Sr_0.2_(Cr_0.2_Mn_0.2_Fe_0.2_Co_0.2_Ni_0.2_)O_3_ and H) the corresponding preparation process diagram. I) TEM image of HE‐LSMO. J) Cycling performance of Li–S batteries assembled based on different cathodes at 1 C. K) Electrochemical performance of HE‐LSMO‐based cathode at high sulfur surface loading. Reproduced with permission.^[^ ^106^ ^]^ Copyright 2022, Elsevier B.V.

Moreover, Fan et al. developed a HEO‐based composite interlayer with the composition (Cu_0.7_Fe_0.6_Mn_0.4_Ni_0.6_Sn_0.5_)O_4_ integrated into carbon nanofibers (HEO/CNFs) via an electrospinning strategy, and applied it to the cathode side of Li–S batteries (**Figure** **15**A,B). The resulting composite exhibits a highly conductive and interconnected carbon nanofiber network, which not only facilitates rapid electron transport but also provides a robust structural framework to accommodate sulfur species. More importantly, the synergistic effect between the physical confinement offered by the carbon substrate and the strong chemisorption capability of the HEO component effectively mitigates the LiPS shuttle effect. Both visual LiPS adsorption experiments and DFT calculations confirm that the incorporation of multiple transition‐metal cations significantly enhances the binding affinity toward sulfur species, thereby promoting their immobilization and catalytic conversion (Figure 15C,D). As a result, the Li–S batteries incorporating the HEO/CNFs interlayer demonstrate impressive high‐rate performance, delivering a capacity of 561 mAh g^−1^ even at a high current density of 6 C, along with remarkable cycling stability over prolonged operation (Figure 15E,F).^[^ ^105^ ^]^ Coincidentally, Tian et al. also engineered a HEO nanofiber (La_0.8_Sr_0.2_(Cr_0.2_Mn_0.2_Fe_0.2_Co_0.2_Ni_0.2_)O_3_) with a perovskite structure (HE‐LSMO) via an electrospinning technique, and employed it as a host material for active sulfur species in Li–S batteries (Figure 15G,H). The incorporation of five transition‐metal cations within the perovskite lattice endowed the HEO nanofibers with versatile catalytic functionalities, which enabled not only the guided nucleation and growth of Li_2_S during discharge but also the efficient decomposition of Li_2_S during charging (Figure 15I). Such bidirectional catalytic behavior is critical for alleviating the sluggish kinetics associated with the solid–solid conversion steps. Moreover, the high configurational entropy of the system contributes to the thermodynamic stabilization of the multiphase structure and facilitates the formation of a large number of catalytically active sites, which effectively modulate the adsorption strength toward LiPS, thereby suppressing their diffusion. Electrochemical evaluations revealed that cells assembled with HEO nanofibers as sulfur hosts exhibited excellent cycling stability at a current density of 1 C (Figure 15J). Notably, even under high sulfur areal loadings of 5.6 and 8.4 mg cm^−2^, the batteries delivered high initial specific capacities and maintained low polarization during the charge–discharge process, reflecting improved reaction kinetics and favorable electrode–electrolyte interfacial characteristics (Figure 15K). This study highlights the potential of perovskite‐type HEO nanofibers as multifunctional hosts in the development of high‐performance sulfur cathodes.^[^ ^106^ ^]^

The integration of HEO into Li–S battery systems has revealed unique advantages arising from their structural robustness, compositional tunability, and strong chemical interactions with LiPS. Owing to the synergistic effect among multiple metal cations within the oxide framework, HEOs not only offer abundant active sites for the effective adsorption of LiPS but also catalyze their redox conversion, thereby suppressing the shuttle effect and accelerating reaction kinetics. Additionally, the inherent lattice stability and high configurational entropy of HEO confer exceptional electrochemical durability under prolonged cycling. These combined features underscore the potential of HEO as versatile cathode hosts or interfacial modifiers.

### 5.3 High‐Entropy Metal Nitrides for Li–S Batteries

Unlike traditional binary or ternary nitrides, high‐entropy metal nitrides (HEN) integrate a complex multimetallic framework with nitrogen coordination, offering distinct electronic structures and defect chemistries that are highly relevant to Li–S conversion reactions. The strong metal‐nitrogen bonding not only enhances electronic conductivity but also modulates the d‐band center of the active metals, thereby tailoring the binding energy toward LiPS. Moreover, the configurational entropy contributes to structural robustness during long‐term cycling, while the uniformly distributed lattice distortions promote abundant catalytic interfaces. These characteristics make high‐entropy nitrides promising candidates for addressing the sluggish kinetics and shuttle effect that hinder sulfur cathode performance. The following section delves into recent studies exploring the catalytic roles, and electrochemical benefits of HEN in Li–S battery systems.

**Figure 16:**
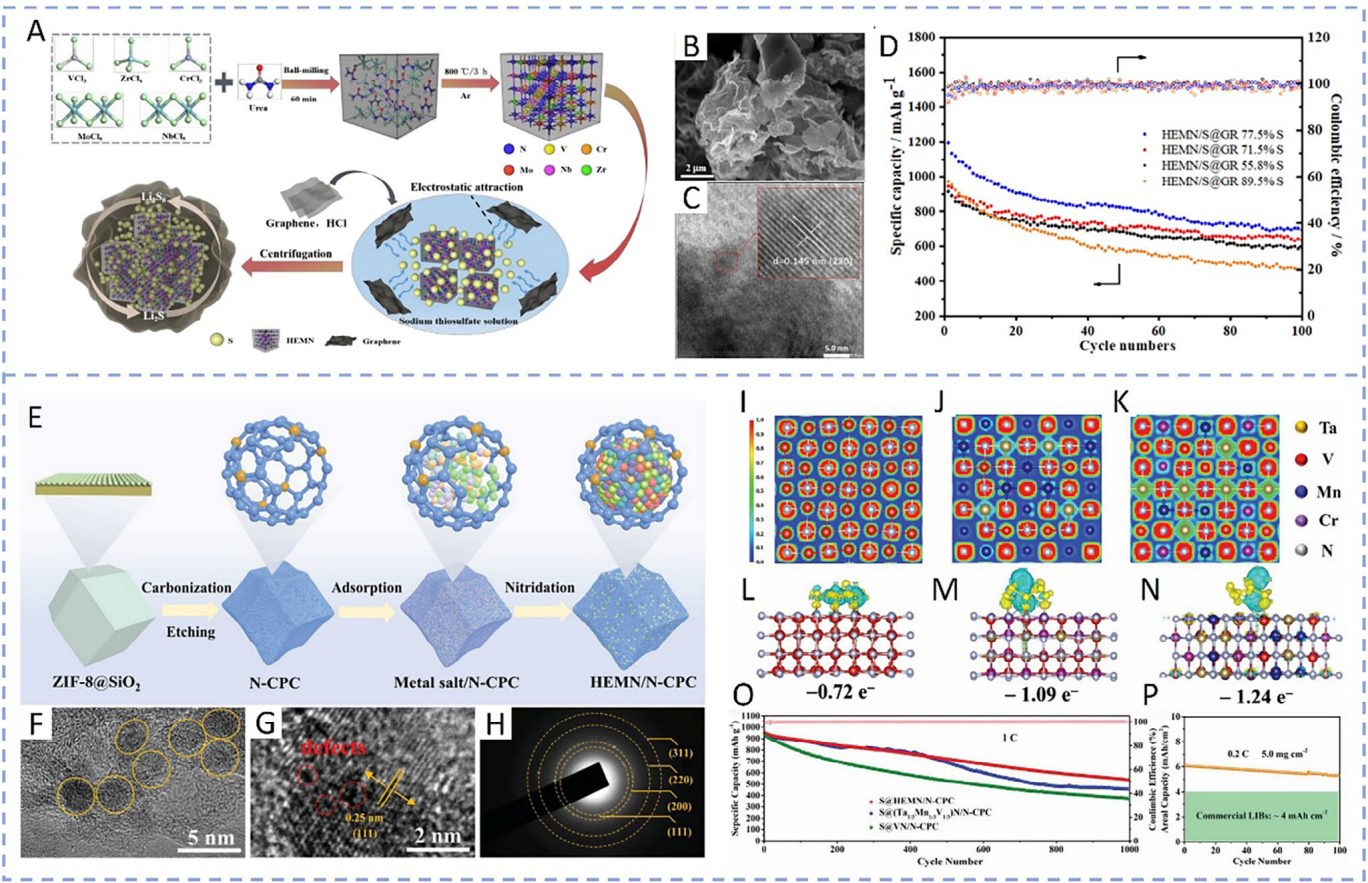
A) Schematic diagram of HEMN/S@GR preparation process. B) SEM and C) HRTEM images of HEMN/S@GR. D) Cycling performance of HEMN/S@GR‐based cathode. Reproduced with permission.^[^ ^84^ ^]^ Copyright 2020, Wiley‐VCH. E) Schematic diagram of the preparation method of HEMN/N‐CPC. F) TEM image of HEMN/N‐CPC. G) HRTEM image of HEMN/N‐CPC H) Selected area electron diffraction of HEMN/N‐CPC. The electronic local function mapping of I) VN, J) (Ta_1/3_Mn_1/3_V_1/3_)N, and K) HEMN substrates. Charge difference density of L) VN‐Li_2_S_6_ and M) (Ta_1/3_Mn_1/3_V_1/3_)N‐Li_2_S_6_ and N) HEMN‐Li_2_S_6_ configurations. O) Cycling stability of S@HEMN/N‐CPC cathode at 1 C. P) Cycle performance of S@HEMN/N‐CPC cathode with the sulfur loading of 5 mg cm^−2^ at 0.2 C. Reproduced with permission.^[^ ^107^ ^]^ Copyright 2024, Wiley‐VCH.

For instance, Li et al. developed a high‐entropy metal nitride (HEMN) composed of Cr, V, Zr, Mo, and Nb via a mechanochemical‐assisted synthesis method (**Figure** **16**A). To enhance electronic conductivity and structural integration, the as‐prepared HEMN particles were subsequently combined with graphene through electrostatic adsorption (HEMN/S@GR), resulting in a uniformly graphene‐wrapped composite structure (Figure 16B,C). This graphene encapsulation effectively suppressed LiPS shuttling by providing both a physical barrier and a conductive matrix, while also significantly reducing the interfacial charge transfer resistance. Moreover, the HEN framework, featuring multiple metal active sites, exhibited strong chemical affinity toward LiPS, thereby promoting their immobilization and accelerating the redox kinetics at the sulfur cathode. As a result, the HEMN/S@GR cathode delivered a high initial specific capacity of 1193 mAh g^−1^ and maintained 695 mAh g^−1^ after 100 cycles at 0.1 C (Figure 16D), demonstrating enhanced cycling performance and interfacial stability.^[^ ^84^ ^]^

Besides, Wang et al. developed a HEMN catalyst embedded in nitrogen‐doped concave porous carbon dodecahedra (N‐CPC) using a zeolitic imidazolate framework (ZIF)‐derived strategy (HEMN/N‐CPC, Figure 16E). This method allowed for the precise incorporation of multiple metal elements (Ta, V, Mn, and Cr) into a single‐phase crystal lattice, ensuring uniform elemental distribution and lattice coherence (Figure 16F–H). The integration of multicomponent metals induced a significant high‐entropy effect, which not only enhanced the thermodynamic stability of the HEMN phase but also had profound effects on its electronic structure. Specifically, DFT calculations revealed that the introduction of diverse transition metal species modulated the d‐band center of the active metal sites and significantly altered the electronic density of states near the Fermi level, thereby optimizing the d–p orbital hybridization between the metal sites and the adsorbed LiPS (Figure 16I–K). This adjustment of electronic structure not only strengthened the chemical anchoring of LiPS through improved orbital overlap but also lowered the energy barriers for the redox reactions of sulfur species (Figure 16L–N). As a result, the HEMN/N‐CPC‐based sulfur cathode exhibited exceptional electrochemical performance, retaining a minimal capacity decay rate of 0.044% per cycle over 1000 cycles at 1 C. Even under a sulfur loading of 5.0 mg cm^−2^, the cell maintained an areal capacity of 5.0 mAh cm^−2^ (Figure 16O,P).^[^ ^107^ ^]^

### 5.4 High‐Entropy Metal Sulfides for Li–S Batteries

Beyond nitrides and oxides, high‐entropy metal sulfides (HES) have recently garnered significant attention in the field of Li–S batteries due to their distinctive chemical properties and structural adaptability. The inherent sulfur‐rich environment in these materials not only ensures good chemical compatibility with sulfur cathodes, but also provides a natural affinity toward LiPS, promoting both their physical confinement and catalytic conversion. Simultaneously, the high configurational entropy promotes the formation of metastable yet robust sulfide phases, while enabling tunable electronic structures that accelerate redox kinetics. These synergistic attributes render HESs a compelling class of multifunctional electrocatalysts for improving the reversibility and stability of Li–S batteries.

**Figure 17:**
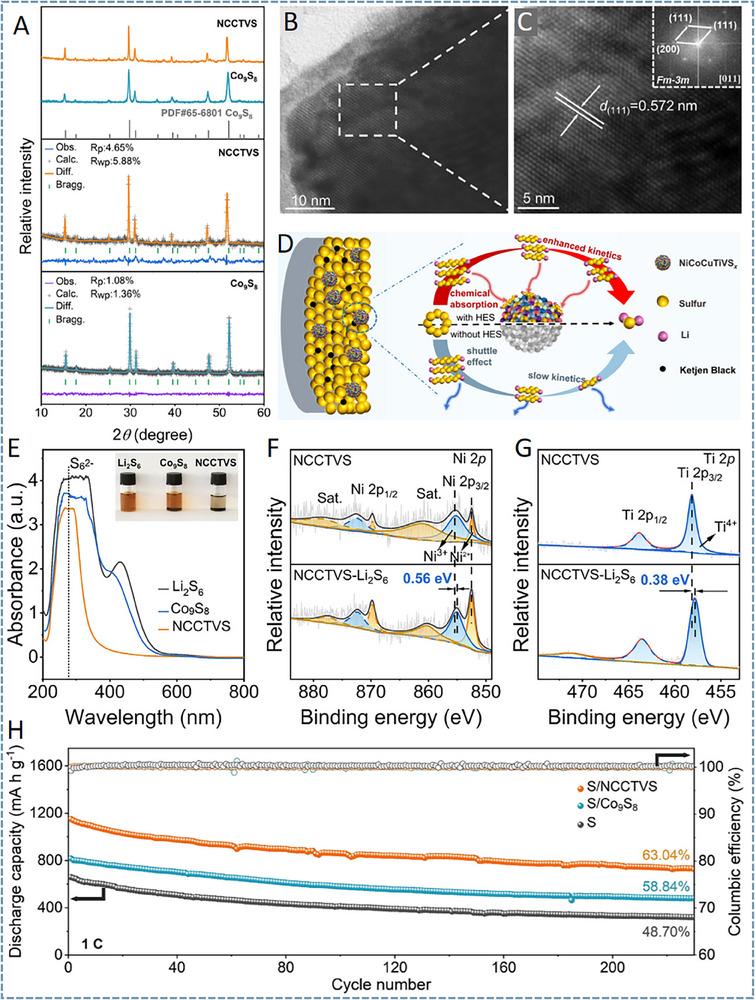
A) XRD patterns of Co_9_S_8_ and NCCTVS and corresponding refinement results. B) HRTEM image of NCCTVS and the C) correspond the local magnified image. D) Schematic diagram of the working mechanism of NCCTVS in Li–S battery system. E) UV–visible absorption spectrum of Li_2_S_6_ solution after adsorption. XPS spectrum of after adsorption F) Ni 2p and G) Ti 2p before and after adsorption for NCCTVS. H) Cycling performance of Li–S batteries based on different cathodes. Reproduced with permission.^[^ ^91^ ^]^ Copyright 2024, Elsevier B.V.

In one representative study, Huang et al. synthesized a HES with the nominal composition NiCoCuTiVS_x_, derived from a Co_9_S_8_ precursor via a solvothermal method (**Figure** **17**A‐C). Owing to the inclusion of multiple transition metals (Ni, Co, Cu, Ti, and V) all of which possess distinct affinities toward LiPS, the resulting HES exhibited synergistic chemical anchoring capabilities (Figure 17D). These metal species provided abundant active sites for strong interactions with long‐chain LiPS, thus effectively suppressing the shuttle effect. The UV–Vis absorption spectra (Figure 17E) of a Li_2_S_6_ solution after contact with the HES material showed complete disappearance of the characteristic S_6_^2‐^ signal, indicating efficient chemical adsorption of LiPS. This was further corroborated by XPS analysis (Figure 17F,G), which revealed noticeable binding energy shifts in the metal active sites (e.g., Ni 2p, Ti 2p), confirming the formation of strong chemical interactions and possible electron transfer between HES and LiPS. Beyond anchoring, the HES also demonstrated a catalytic effect by accelerating the redox kinetics of LiPS. The multicomponent nature of HES likely enabled modulation of the electronic structure, enhancing the electronic conductivity and optimizing the adsorption‐conversion synergy. Consequently, the S/NCCTVS cathode delivered excellent cycling stability, maintaining a reversible capacity of 725.4 mAh g^−1^ over 230 cycles at 1 C (Figure 17H), highlighting the functionality of HES as redox catalysts in Li–S batteries.^[^ ^91^ ^]^

**Figure 18:**
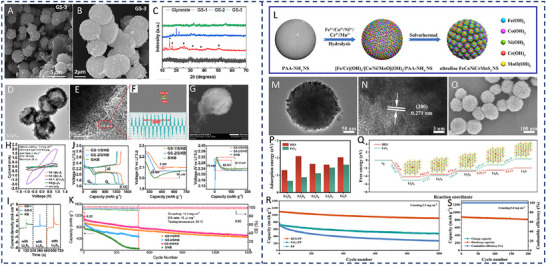
SEM images of A) GS‐3 and B) partial enlarge image. C) XRD patterns of different samples. D) TEM and E) HRTEM images of GS‐3. F) Inverse Fourier transform image of a specific selected area. G) STEM image of GS‐3. H) CV curves of symmetric cells of different working electrodes with Li_2_S_6_ electrolyte. I) Comparison of chronoamperometry profiles of different working electrodes. J) Galvanostatic charge/discharge curves of various cathodes. K) Long‐term cycling performance of different cathodes for 1500 cycles. Reproduced with permission.^[^ ^108^ ^]^ Copyright 2025, Wiley‐VCH. L) Schematic diagram of the preparation process of FeCoNiCrMnS_2_. M) TEM image of FeCoNiCrMnS_2_. N) HRTEM image of FeCoNiCrMnS_2_. O) SEM image of FeCoNiCrMnS_2_. P) Binding energy and Q) Gibbs free energy curves of different sulfur species on HES and FeS_2_ surfaces. R) Long‐term cycling stability of Li–S cells with different separators. S) Cycling performance of Li–S battery based on HES/PP assembly at 0.5 C with a sulfur loading of 5 mg cm^−2^. Reproduced with permission.^[^ ^92^ ^]^ Copyright 2025, Royal Society of Chemistry.

Furthermore, Cheng and collaborators synthesized three types of HES, denoted as GS‐1 (NiCoFeMgZnS_x_), GS‐2 (NiCoCuMgZnS_x_), and GS‐3(NiCoFeMgTiS_x_), by employing a glycerol‐assisted hydrothermal method using glycerol as a soft template. These materials exhibited microspherical morphologies, with GS‐3 showing the most uniform elemental distribution and the most well‐defined spherical architecture (**Figure** **18**A–C). The enhanced structural integrity and compositional homogeneity of GS‐3 are favorable for exposing more accessible active sites and improving electrochemical performance (Figure 18D–G). In symmetric cell tests using Li_2_S_6_‐containing electrolyte, the GS‐3 electrode delivered a significantly higher redox current response compared to GS‐1 and GS‐2, indicating faster LiPS redox conversion kinetics on the surface of GS‐3 (Figure 18H,I). This suggests that GS‐3 provides more efficient catalytic sites for promoting the reversible transformation between soluble and insoluble sulfur species. Furthermore, the Li–S cell assembled with a GS‐3/S/KB composite cathode exhibited reduced polarization and lower overpotentials during the charge–discharge process, highlighting the high catalytic activity of GS‐3 toward LiPS conversion reactions (Figure 18G). As a result, the device demonstrated enhanced cycling stability, underscoring the synergistic benefits of high‐entropy composition, microspherical morphology, and optimized surface chemistry in improving the electrochemical performance of Li–S batteries (Figure 18K).^[^ ^108^ ^]^

Li et al. synthesized FeCoNiCrMnS_2_ (HES) nanospheres via a facile template‐assisted method and utilized them as a functional modification layer coated on polypropylene separators (HES/PP) to enhance the electrochemical performance of Li–S batteries (Figure 18L). Owing to their ultrafine particle size, the HES nanospheres expose abundant surface‐active sites, enabling efficient adsorption and chemical anchoring of LiPS, thereby suppressing the shuttle effect (Figure 18M–O). Simultaneously, these exposed sites also act as electrocatalytic centers to facilitate the redox conversion of sulfur species. DFT calculations further support these findings, demonstrating that the multi‐metallic configuration of HES provides significantly enhanced binding energies with LiPS species, indicative of strong chemical interactions (Figure 18P). Moreover, the energy barriers for LiPS conversion reactions on the HES surface are substantially reduced, thereby accelerating the overall reaction kinetics (Figure 18Q). As a result of this functionality, Li–S batteries employing the HES/PP composite separator deliver markedly improved rate capabilities and exhibit excellent cycling stability (Figure 18R). Even under higher sulfur areal loading conditions, the cell maintains a high reversible capacity of 814.4 mAh g^−1^ after 200 cycles at 0.2 C, highlighting the significant potential of HES‐based interlayers in advancing the practical application of Li–S battery systems (Figure 18S).^[^ ^92^ ^]^

### 5.5 High‐Entropy Metal Phosphides for Li–S Batteries

Unlike conventional oxides, nitrides, or sulfides, phosphide‐based materials offer a distinct combination of high intrinsic electronic conductivity and strong chemical affinity toward LiPS, making them especially appealing for electrocatalytic applications in Li–S batteries. When incorporated into a high‐entropy configuration, these materials exhibit not only enhanced charge transport properties but also tunable electronic structure. The configurational entropy inherent in high‐entropy metal phosphides (HEP) leads to a disruption of conventional bonding environments, which in turn facilitates the formation of low‐coordination metal sites and modulates local electronic state distribution, both of which are essential for boosting LiPS adsorption and redox kinetics. These features enable HEP to simultaneously suppress the shuttle effect and accelerate sulfur conversion reactions. Thus, HEP emerge as a promising class of multifunctional electrocatalysts, offering structural robustness, high electrical conductivity, and active surface tunability. The incorporation of HEP into sulfur cathode architectures presents an effective and rational strategy to enhance the electrochemical performance of Li–S batteries.

**Figure 19:**
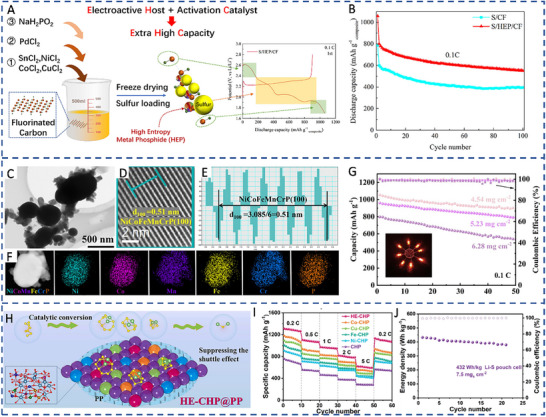
A) Schematic diagram of the preparation process and capacity contribution mechanism of Pd_0.34_Sn_0.15_Ni_0.05_Co_0.09_Cu_0.29_P_0.08_ for Li–S cell. B) Cycling performance of different cathodes. Reproduced with permission.^[^ ^109^ ^]^ Copyright 2025, Wiley‐VCH. C) TEM image, D) HRTEM image, and E) the corresponding line profiles of NiCoMnFeCrP. F) The corresponding elemental mapping images of NiCoMnFeCrP. G) The cycling performance of NiCoMnFeCrP‐based cathode with different sulfur loading. Reproduced with permission.^[^ ^52^ ^]^ Copyright 2024, Elsevier B.V. H) Schematic diagram of the working mechanism of HE‐CHP@PP separator in Li–S batteries. I) Rate performance of the HE‐CHP@PP. (J) Cycling efffciency of the Li–S pouch cell with HE‐CHP@PP at 0.05 C. Reproduced with permission.^[^ ^90^ ^]^ Copyright 2024, Elsevier B.V.

In this sense, Gao et al. developed a novel HEP, Pd_0.34_Sn_0.15_Ni_0.05_Co_0.09_Cu_0.29_P_0.08_, as a catalytic host for the cathode of Li–S batteries. By in situ deposition on a fluorinated carbon (CF) matrix, the electrochemical performance of the battery was markedly improved (**Figure** **19**A). Traditional host materials are often electrochemically inert, limiting the practical energy density of Li–S batteries. This study is the first to employ CF as an electroactive sulfur host, with its high theoretical capacity (approximately 1000 mAh g^−1^) providing additional capacity to the cathode. However, the high polarization and low conductivity of CF constrained its application. By introducing HEP catalysts, the redox activity of CF was optimized, and the kinetics of sulfur conversion reactions were accelerated. The multi‐metal synergistic effects of HEP (e.g., the high catalytic activity of Pd and the high conductivity of Cu) provided abundant active sites and reduced the nucleation energy barrier of Li_2_S. Electrochemical tests revealed that the S/HEP/CF cathode achieved an initial discharge capacity of 1059.2 mAh g^−1^ at 0.1 C, significantly outperforming the S/CF electrode (Figure 19B). Additionally, the decomposition of CF generates LiF and a carbon matrix, further enhancing the stability of the electrode/electrolyte interface.^[^ ^109^ ^]^

Moreover, Wang et al. successfully prepared NiCoMnFeCrP HEP via a sol–gel method and applied them to modify Li–S cell separators. Leveraging the synergistic effects of its multi‐metal components and highly dispersed active sites, this material exhibited excellent LiPS adsorption and catalytic conversion capabilities. Material characterization revealed that the pentanary metal phosphide featured a regular spherical morphology (500–800 nm) and uniform elemental distribution (Figure 19C–F). HRTEM confirmed its typical Ni_2_P crystal structure characteristics, with a lattice spacing of 0.514 nm. This multi‐component design endowed the material with outstanding polysulfide adsorption and catalytic conversion performance. Experiments showed that the capacity decay rates of the NiCoMnFeCrP‐modified separator after 100 cycles at 0 and 60 °C were only 0.04% and 0.23%, respectively, demonstrating excellent temperature adaptability. Furthermore, under higher sulfur loading (4.54 mg cm^−2^), the Li–S battery equipped with this modified separator achieved a high areal capacity of 4.78 mAh cm^−2^ (Figure 19G), significantly surpassing the commercial standard of traditional Li‐ion batteries (4 mAh cm^−2^) and highlighting the practical potential of HEP in Li–S batterie.^[^ ^52^ ^]^

In addition, although phosphides exhibit excellent catalytic activity, their structural stability is limited. Researchers have addressed this by introducing phosphate groups (PO_4_^3‐^) to construct high‐entropy phosphates, which retain the advantages of multi‐metal synergy while leveraging the rigid framework of PO_4_^3‐^ to enhance material stability. Specifically, Zhu et al. prepared a novel high‐entropy hydroxyphosphate, Co_0.29_Ni_0.15_Fe_0.33_Cu_0.16_Ca_3.9_(PO_4_)_3_(OH) (HE‐CHP), via a metal cation exchange method and successfully applied it to modify Li–S battery separators. HE‐CHP enhanced LiPS adsorption through the formation of metal‐S bonds, while its multi‐metal active sites synergistically promoted the catalytic conversion of LiPS (Figure 19H). Rate capability tests demonstrated that the battery equipped with the HE‐CHP@PP separator exhibited excellent capacity retention across various current densities, from 0.2 to 5 C (Figure 19I). The initial discharge capacity reached 1308.5 mAh g^−1^ at 0.2 C, and even at a high current density of 5 C, it maintained a capacity of 621.3 mAh g^−1^. When the current density was restored to 0.2 C, the capacity recovered to 1133.9 mAh g^−1^, indicating good electrochemical reversibility. In a pouch cell demonstration, the CNT@S||Li pouch cell with the HE‐CHP@PP separator achieved a high energy density of 432 Wh kg^−1^ under a high sulfur loading of 7.5 mg cm^−2^ and successfully illuminated an LED (Figure 19J), marking a significant breakthrough in the practical application of HEP materials in energy storage devices.^[^ ^90^ ^]^

### 5.6 High‐Entropy MXene for Li–S Batteries

Recent studies have begun to explore the integration of high‐entropy design principles with two‐dimensional (2D) materials‐most notably, MXene. As a family of transition metal carbides, nitrides, and carbonitrides, MXene have garnered substantial interest in electrochemical energy storage owing to their high electrical conductivity, large specific surface area, tunable surface terminations, and excellent mechanical flexibility. Nevertheless, conventional single‐metal MXene often suffer from limited catalytic activity, poor chemical stability, and insufficient active site diversity when employed in Li–S batteries. To address these challenges, the concept of high‐entropy MXene has emerged, wherein multiple transition metal elements are incorporated into the MXene framework to form entropy‐stabilized, compositionally complex structures. This innovative strategy allows for the synergistic tuning of the electronic structure, optimization of active site distributions, and improvement of redox stability. Consequently, high‐entropy MXene (HE‐MXene) offer enhanced catalytic activity for LiPS conversion and improved electrochemical performance, positioning them as a highly promising class of multifunctional materials for Li–S batteries.

**Figure 20:**
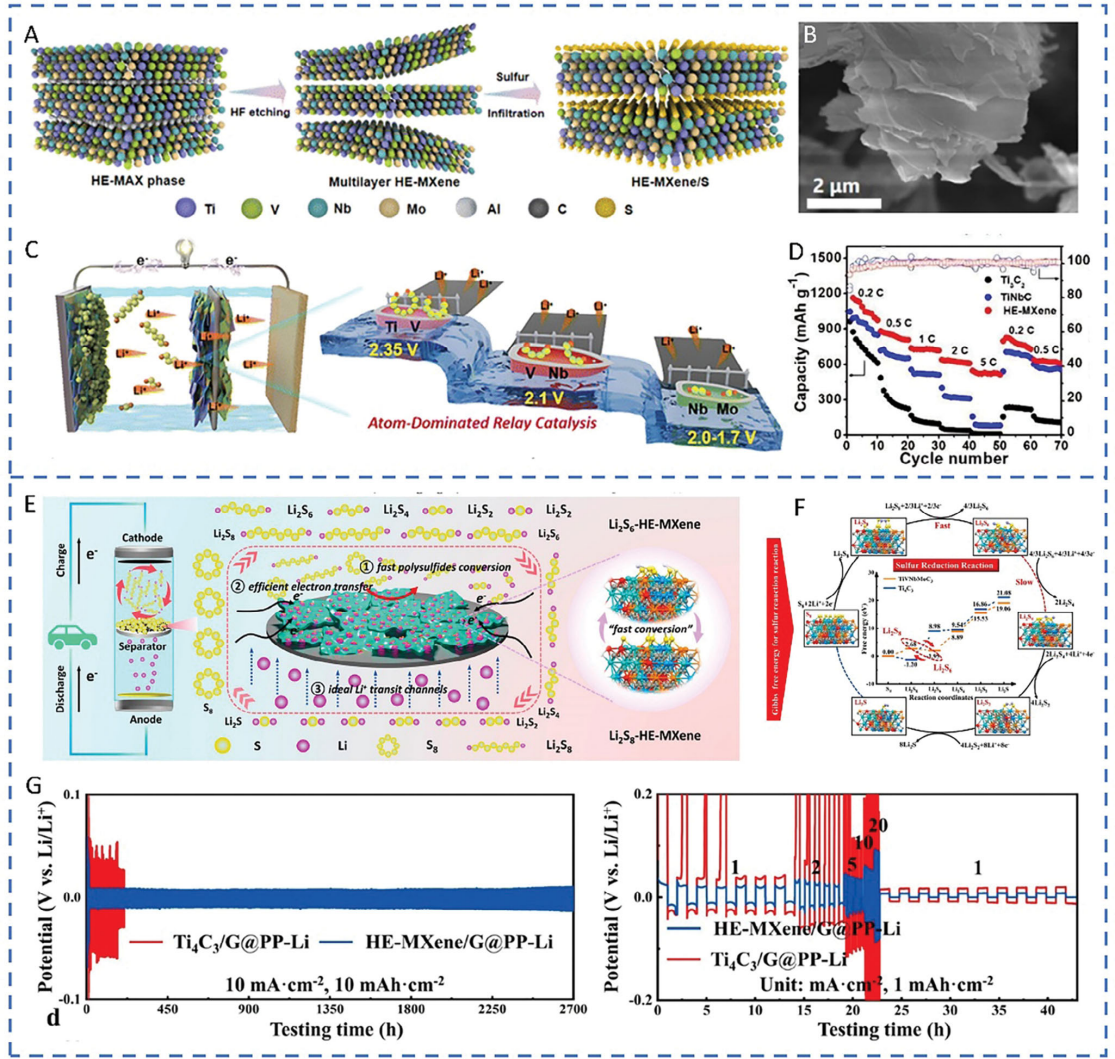
A) Schematic representation of the preparation process for the HE‐MXene/S composite. B) The SEM image of TiVNbMoC_3_ HE‐MXene. C) Diagram illustrating a Li–S battery design utilizing HE‐MXene both as a sulfur host and a separator coating, featuring a relay catalysis mechanism for LiPS and a robust lattice structure that promotes dendrite‐free lithium anode growth. D) Battery rate performance based on different sulfur host and separator modification layers. Reproduced with permission.^[^ ^94^ ^]^ Copyright 2024, Royal Society of Chemistry. E) Schematic depiction of the HE‐MXene/G@PP composite, highlighting its role in suppressing LiPS diffusion and facilitating efficient electron transport in Li–S batteries. F) Comparison of sulfur reduction energetics on Ti_4_C_3_ and HE‐MXene from DFT, with inset illustrations of the optimized configurations. G) Cycling stability of Li||Li symmetric cells using HE‐MXene/G@PP and Ti_4_C_3_/G@PP under a current density of 10 mA cm^−2^ and areal capacity of 10 mAh cm^−2^. H) The rate capacity of Li||Li symmetric cells with HE‐MXene/G@PP and Ti_4_C_3_/G@PP. Reproduced with permission.^[^ ^110^ ^]^ Copyright 2024, American Chemical Society.

For instance, Xu et al. designed and synthesized a HE‐MXene composed of Ti, V, Nb, and Mo (TiVNbMoC_3_), which was employed as both a sulfur cathode host and a functional modification layer for the separator in Li–S batteries (**Figure** **20**A). As shown in Figure 20B, the exfoliated HE‐MXene obtained via HF etching exhibits excellent interlayer separation characteristics, facilitating exposure of more active sites and enhancing electrode/electrolyte interfacial contact area. Combined theoretical calculations and experimental analyses reveal that the material effectively modulates the d‐band centers of the transition metals while maintaining lattice stability, achieving moderate adsorption strength toward LiPS and superior catalytic activity. As illustrated in Figure 20C, Ti, V, Nb, and Mo atomic sites dominate different stages of the sulfur reduction reaction, establishing an atomically driven “relay” catalytic mechanism that promotes continuous capture and conversion of LiPS, effectively suppressing the shuttle effect. Figure 20D shows the discharge performance of Li–S batteries using HE‐MXene as the sulfur host and separator modification layer under various current rates. The batteries maintain a high capacity of 545.2 mAh g^−1^ even as the rate increases from 0.2 C to 5 C, and the capacity recovers to 826.2 mAh g^−1^ when the rate returns to 0.2 C, demonstrating excellent rate capability and electrochemical reversibility. The introduction of multiple transition metal elements to construct HE‐MXene enables synergistic regulation of lattice stability and electronic structure, substantially improving catalytic activity and stability at both sulfur cathode and lithium anode interfaces in Li–S batteries.^[^ ^94^ ^]^

Furthermore, based on the above HE‐MXene material, Xu et al. further constructed a HE‐MXene/graphene composite structure applied as a separator modification layer to fully exploit the chemical anchoring and catalytic advantages of the multi‐element active centers in HE‐MXene, while leveraging high electrical conductivity of graphene to accelerate LiPS conversion. This multi‐element quasi‐atomically doped HE‐MXene/graphene composite material (HE‐MXene/G@PP) was fabricated as a functionalized separator coating for Li–S batteries (Figure 20E). The composite not only significantly enhances electrical conductivity to facilitate rapid electrochemical reactions of LiPS but also achieves efficient chemical adsorption of LiPS through multiple transition metal active sites, improving interfacial stability. Theoretical calculations show that HE‐MXene reduces the Gibbs free energy change (Δ*G*) for LiPS reduction from 21.08 eV of Ti_4_C_3_ to 19.06 eV (Figure 20F), indicating more favorable thermodynamics for the reduction of LiPS intermediates. Electrochemical testing further confirms the excellent cycling stability and interface performance of this composite. As shown in Figure 20G,H, under the condition of 10 mA cm^−2^/10 mAh cm^−2^, the HE‐MXene/G@PP lithium symmetric cell maintains a stable overpotential of approximately 22.5 mV, significantly lower and more stable than the larger and fluctuating overpotential observed for Ti_4_C_3_/G@PP. This outstanding cycling and interface stability are mainly attributed to the high mechanical strain induced by the multiple metal/non‐metal active sites in HE‐MXene, effectively promoting uniform and dendrite‐free Li deposition. The HE‐MXene/graphene composite, as a highly conductive and multi‐active‐center material used for separator modification, enhances LiPS anchoring and catalysis, thereby improving cycling stability and uniform lithium plating.^[^ ^110^ ^]^

**Figure 21:**
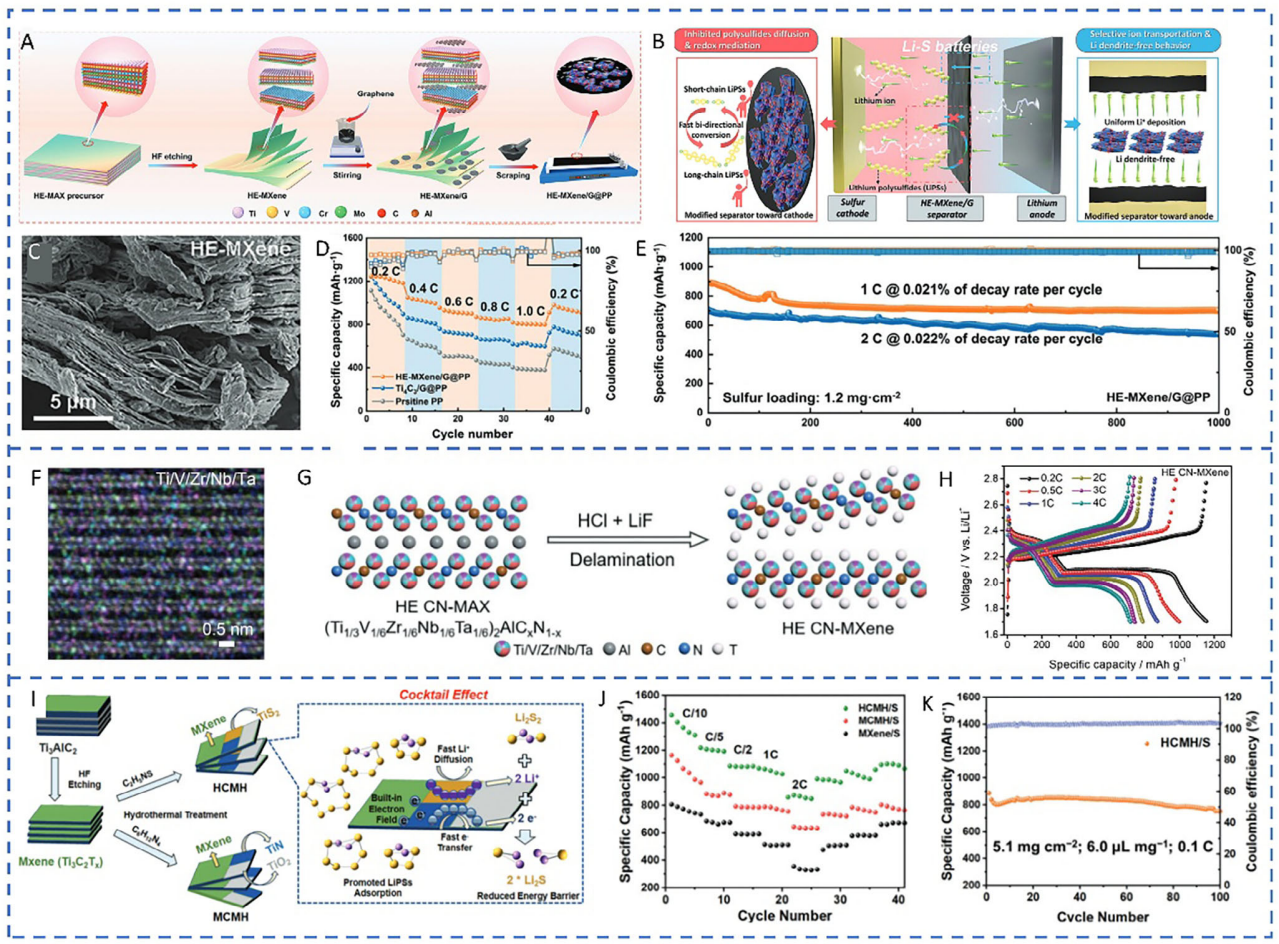
A) Schematic diagram of the preparation process of HE‐MXene/G@PP. B) Schematic diagram of the mechanism by which HE‐MXene/G@PP promotes the conversion of LiPS to Li_2_S_2_/Li_2_S and inhibits the growth of Li dendrites. C) SEM image of HE‐MXene. D) Rate performance the Li–S cell with HE‐MXene/G@PP. E) Cyclic stability of HE‐MXene/G@PP at 1 and 2 C current densities. Reproduced with permission.^[^ ^111^ ^]^ Copyright 2024, Wiley‐VCH. F) SEM element mapping image of Ti_1/3_V_1/6_Zr_1/6_Nb_1/6_Ta_1/6_)_2_AlC_x_N_1‐x_. G) Schematic diagram of the preparation route of HE CN‐MXene. H) Galvanostatic charge and discharge profiles of HE CN‐MXene‐based cathode at different rates. Reproduced with permission.^[^ ^49^ ^]^ Copyright 2021, Wiley‐VCH. I) The preparation process of MCMH and HCMH materials, and the schematic diagram of the mechanism of HCMH promoting the LiPS conversion process. J) Rate performance of Li–S batteries based on different cathodes. K) Cycling performance of the Li–S cell with HCMH/S cathode under a sulfur loading of 5.1 mg cm^−2^. Reproduced with permission.^[^ ^96^ ^]^ Copyright 2024, Wiley‐VCH.

On the other hand, based on the previously demonstrated advantages of high‐entropy MXene/graphene composites in terms of multi‐metallic synergistic catalysis and superior electrical conductivity, Wang et al. developed a quasi‐atomically doped TiVCrMoC_3_T_x_/graphene composite (TiVCrMoC_3_T_x_/G@PP). By selectively etching the high‐entropy MAX precursor to obtain layered HE‐MXene, followed by compositing with graphene and loading onto a PP separator, a functionalized separator structure with both high electrical conductivity and multiple active sites was constructed (**Figure** **21**A). This design aims to simultaneously regulate LiPS species and suppress Li dendrite growth (Figure 21B). Morphological analysis revealed that the HE‐MXene after HF etching exhibited a characteristic accordion‐like layered structure (Figure 21C). Compared with untreated HE‐MAX, the interlayer spacing was significantly expanded, which facilitates Li ions storage and transport, thereby enhancing interfacial reaction kinetics. Benefiting from its unique 2D structure and conductive network, the TiVCrMoC_3_T_x_/G@PP‐modified separator exhibited excellent rate performance under various current densities. As shown in Figure 21D, the discharge capacities at current rates ranging from 0.2 C to 1 C were markedly higher than those using Ti_4_C_3_T_x_/G@PP and the pristine PP separator. Notably, when the rate returned to 0.2 C, the capacity recovered to 928.8 mAh g^−1^, indicating favorable rate‐cycle stability and electrochemical reversibility. Furthermore, the TiVCrMoC_3_T_x_/G@PP‐based cell demonstrated outstanding long‐term cycling stability. As shown in Figure 21E, after 1000 cycles at 1 and 2 C, the capacity retention reached 78.77% and 77.78%, respectively, with per‐cycle capacity decay rates of only 0.021% and 0.022%, highlighting its structural integrity and electrochemical durability. This work synergistically integrates high‐entropy 2D materials with functionalized carbon frameworks, proposing an efficient strategy to regulate LiPS shuttling while inhibiting Li dendrite formation.^[^ ^111^ ^]^

Besides, Yang et al. successfully synthesized a novel high‐entropy carbonitride MAX phase (HE CN‐MAX, (Ti_1/3_V_1/6_Zr_1/6_Nb_1/6_Ta_1/6_)_2_AlC_x_N_1‐x_) using a metallurgical approach by combining a medium‐entropy MAX phase (Zr_1/3_Nb_1/3_Ta_1/3_)_2_AlC with other MAX phases (Ti_4_AlN_3_ and V_2_AlC). As shown in Figure 21F, atomic‐resolution EDS mapping of HE CN‐MAX clearly revealed a homogeneous distribution of Ti, V, Zr, Nb, and Ta elements within the M_n+1_X_n_ layers, providing strong evidence for uniform doping and solid‐solution characteristics. Based on this high‐entropy MAX precursor, the preparation pathway toward HE CN‐MXene was further illustrated (Figure 21G). Selective etching with HCl/LiF solution was employed to remove Al, followed by ultrasonic exfoliation to obtain ultrathin and structurally intact HE CN‐MXene nanosheets. In terms of electrochemical performance, the Li–S batteries based on HE CN‐MXene exhibited two distinct and stable voltage plateaus even under high‐rate conditions (Figure 21H), suggesting excellent reversibility and kinetic stability of sulfur redox reactions during rapid charge/discharge processes, thereby enhancing the battery's rate performance. Collectively, this strategy effectively addressed the phase separation challenges caused by low entropy differences during traditional synthesis, and achieved the successful fabrication of high‐performance HE‐MXene materials, opening up new possibilities for the application of high‐entropy materials in energy storage systems.^[^ ^49^ ^]^

Building on the above advancements, the concept of high‐entropy heterostructures was further proposed. This approach involves the intelligent integration of multiphase catalytic components with MXenes to construct a highly disordered interfacial environment, thereby synergistically optimizing SRR kinetics. Zhang et al. developed a high‐entropy MXene‐based heterostructure composed of TiO_2_, TiN, TiS_2_, and Ti_3_C_2_T_x_ via a one‐step solution treatment process involving simultaneous oxidation, nitridation, and sulfidation of MXene (Figure 21I). This structure retained the excellent conductivity of MXene while significantly enhancing its adsorption capacity toward LiPS. Compared to medium‐complexity heterostructures (MCMH) and pristine MXene, the high‐entropy heterostructure exhibited superior electrocatalytic performance. Electrochemical analysis revealed a lower Tafel slope and higher electron transfer number, indicating stronger catalytic activity in SRR. Further theoretical calculations and experimental verification showed that the high‐entropy composite (HCMH) could significantly reduce the Gibbs free energy barrier for key reaction steps (e.g., Li_2_S deposition and decomposition), thus improving overall reaction kinetics. The HCMH/S electrode also demonstrated excellent rate performance (Figure 21J), delivering a high discharge capacity of 874 mAh g^−1^ even at 2 C, with well‐defined dual voltage plateaus preserved during rapid cycling, indicative of outstanding reversibility and voltage stability. To evaluate the practical applicability of this high‐entropy heterostructure, its performance under high sulfur loading (5.1 mg cm^−2^) and low electrolyte‐to‐sulfur ratio (6 µL mg^−1^) was assessed. Impressively, the HCMH/S cell maintained a reversible areal capacity of over 4.1 mAh cm^−2^ after 100 cycles (Figure 21K).^[^ ^96^ ^]^

### 5.7 High‐Entropy Prussian blue analogous for Li–S batteries

Expanding beyond traditional high‐entropy material systems, high‐entropy Prussian blue analogues (HE‐PBA) have recently emerged as a structurally versatile and chemically tailorable class of frameworks for Li–S battery applications. Featuring open three‐dimensional lattice structures and tunable multimetallic compositions, HE‐PBA combine the entropy‐stabilized configuration with accessible ion diffusion channels, enabling efficient sulfur species regulation. Unlike dense‐phase alloys or ceramic materials, the porous and crystalline nature of PBA allows for both physical confinement of LiPS and active catalytic interfaces, while the incorporation of diverse transition metal centers enhances redox kinetics via synergistic electronic modulation. These distinctive characteristics position HE‐PBA as a compelling candidate for multifunctional sulfur host design in Li–S batteries.

**Figure 22:**
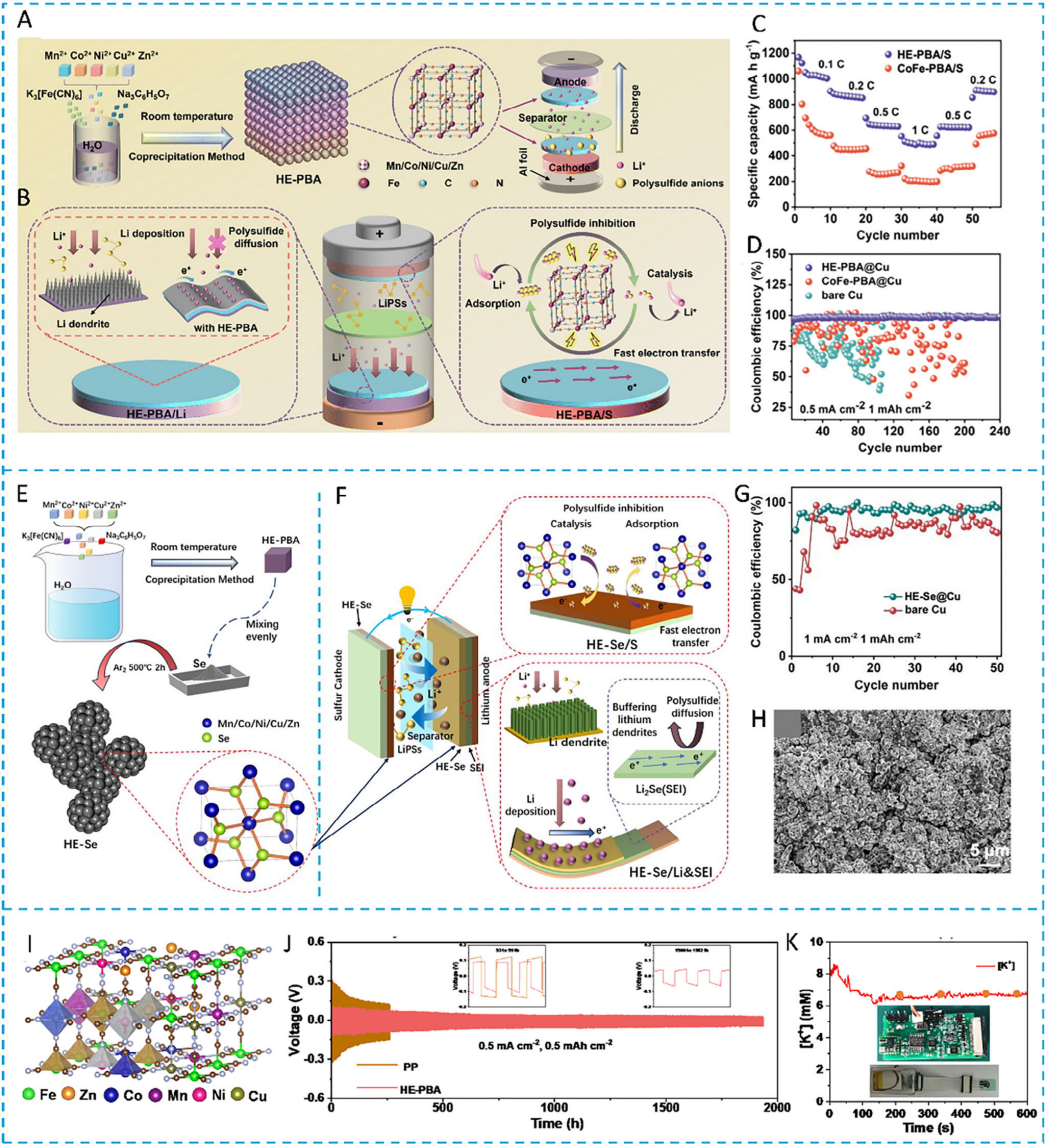
A) Diagram of HE‐PBA synthesis via a room‐temperature coprecipitation approach. B) Conceptual representation of the Li–S battery performance enabled by multifunctional HE‐PBA frameworks. C) Electrochemical rate capability of the HE‐PBA/S cathode. D) Comparative voltage profiles of lithium plating and stripping processes were recorded for HE‐PBA@Cu/Li, CoFe‐PBA@Cu/Li, and bare Cu/Li hosts at 0.5 mA cm^−2^ with a total areal capacity of 1 mAh cm^−2^. Reproduced with permission.^[^ ^51^ ^]^ Copyright 2024, Royal Society of Chemistry. E) Schematic illustration of the straightforward synthesis route for the HE‐Se sample and F) its bifunctional roles within the Li–S battery. G) Coulombic efficiency comparison of HE‐Se@Cu/Li and bare Cu/Li electrodes at a current density of 1.0 mA cm^−2^ with a capacity of 1.0 mAh cm^−2^. H) SEM characterization of HE‐Se@Cu and bare Cu current collectors after 10 cycles at a current density of 1.0 mA cm^−2^ with a Li deposition capacity of 10 mAh cm^−2^. Reproduced with permission.^[^ ^112^ ^]^ Copyright 2024, Wiley‐VCH. I) Illustration of the crystal structure model, where Fe, Co, Zn, Mn, Ni, and Cu atoms are color‐coded as green, dark blue, brown, light purple, pink, and bluish yellow, respectively. J) Cycling stability of Li‐Li symmetric cells tested at 0.5 mA cm^−2^ with a total capacity of 0.5 mAh cm^−2^. K) Wearable electrochemical sensor for real‐time monitoring of K^+^ concentration fluctuations. The fulvous dots represent measurements obtained via ICP‐MS analysis of sweat. Inset: photograph of the wearable electrochemical sensor. Reproduced with permission.^[^ ^113^ ^]^ Copyright 2025, Elsevier B.V.

Interestingly, Li et al. introduced a high‐entropy strategy into the PBA material system and successfully synthesized a compositionally HE‐PBA cubic structure Mn_0.4_Co_0.4_Ni_0.4_Cu_0.4_Zn_0.4_[Fe(CN)_6_]_2_, which served as a dual‐functional host material for both the cathode and anode in Li–S batteries (**Figure** **22**A). On the cathode side, the incorporation of multiple metal ions within HE‐PBA provides abundant anchoring sites for LiPS, while the open three‐dimensional framework promotes rapid electrolyte infiltration and effectively suppresses the shuttle effect. On the anode side, the HE‐PBA demonstrates strong lithiophilicity, which facilitates uniform Li nucleation and deposition, thereby significantly inhibiting the growth of lithium dendrites (Figure 22B). Electrochemical performance tests demonstrate that the Li–S cells based on HE‐PBA exhibit excellent rate capability, delivering specific capacities of 1076.4, 892.6, 664.3, and 512.8 mAh g^−1^ at current densities of 0.1, 0.2, 0.5, and 1 C, respectively. When the current density is reverted from 1 C back to 0.2 C, the capacity remains high at 865.7 mAh g^−1^, indicating good electrochemical reversibility and structural stability (Figure 22C). Compared with the conventional CoFe‐PBA/S cathode, the HE‐PBA/S electrode consistently exhibited higher capacity across all current rates, highlighting its advantages in promoting LiPS conversion kinetics and enhancing charge transport efficiency. Furthermore, long‐term voltage response profiles (Figure 22D) further confirm the exceptional dendrite suppression capability of the HE‐PBA architecture. The HE‐PBA@Cu/Li composite electrode maintained a low voltage hysteresis of approximately 29.4 mV even after extended cycling, reflecting excellent long‐term overpotential stability. In contrast, the CoFe‐PBA@Cu/Li and bare Cu/Li electrodes displayed much higher overpotentials of 53.6 and 57.3 mV, respectively, and showed disrupted voltage pulse periodicity after 20 h of cycling, which was attributed to short circuits caused by uncontrolled dendrite growth.^[^ ^51^ ^]^

Building upon the successful application of HE‐PBA as bifunctional scaffolds for both cathode and anode in Li–S batteries, the high‐entropy design strategy has been further extended to related material systems. In this context, Li et al. synthesized a high‐entropy selenide (HE‐Se) derived from Prussian blue precursors via a coprecipitation method combined with a selenization process, serving as an advanced bifunctional host with excellent catalytic activity and lithophilicity for suppressing Li dendrite growth at the anode and enhancing sulfur redox kinetics at the cathode (Figure 22E). Comprehensive experimental characterizations demonstrate that the HE‐Se material not only effectively catalyzes the conversion of Li and regulates Li ions deposition behavior, but also exhibits superior electrical conductivity due to the synergistic effect of its multimetallic composition (Figure 22F). Electrochemical performance tests reveal that the HE‐Se@Cu current collector exhibits a higher Coulombic efficiency (94.8%) and improved cycling stability under a current density of 1.0 mA cm^−2^ (Figure 22G), indicating a significant advantage in promoting uniform Li plating and stripping. Morphological observations (Figure 22H) further confirm that even after 10 cycles of deposition and stripping, the surface of HE‐Se@Cu maintains a homogeneous Li deposition morphology without apparent dendrite formation. In contrast, the bare Cu current collector, unable to effectively guide Li deposition, suffers from severe dendrite growth, rapid electrolyte consumption, and separator puncture, leading to internal short circuits and inferior cycling performance. Attributed to the high lithophilicity of HE‐Se, this material substantially lowers both equilibrium and nucleation overpotentials during lithium deposition, thereby enabling uniform lithium plating and effectively inhibiting dendrite formation. In summary, this work centers on the HE‐Se to construct a bifunctional electrode scaffold capable of catalyzing LiPS conversion and regulating lithium deposition simultaneously, providing a novel material design strategy for advancing high‐performance Li–S batteries.^[^ ^112^ ^]^

Benefiting from its unique electronic structure and multiple active sites, HE‐PBA exhibits outstanding bidirectional catalytic performance in Li–S systems. Simultaneously, its excellent electronic and ionic conductivity also renders it a promising electrochemical material with dual functions in energy storage and sensing. Notably, Han et al. developed an integrated wearable electronic system, in which an HE‐PBA modified Li–S serves as the power module, integrated with a potentiometric potassium ion sensor to achieve real‐time monitoring of K^+^ concentrations in sweat during physical activity. As illustrated in Figure 22I, the crystal structure of HE‐PBA comprises Ni, Co, Zn, Mn, and Cu elements co‐occupying the M1 sites coordinated with nitrogen within the PBA framework, forming a typical high‐entropy structure. This multi‐metal co‐occupancy not only provides a structural basis for the synergistic catalysis of multiple active centers but also facilitates the regulation of the material's electronic structure and stability. These optimized structural characteristics are clearly reflected in the electrochemical performance. As shown in Figure 22J, under a current density of 0.5 mA cm^−2^ and a plating/stripping capacity of 0.5 mAh cm^−2^, the Li‐Li symmetric cell exhibits an ultralow nucleation overpotential and negligible voltage fluctuation over a prolonged cycling duration of 1937 h. This indicates the excellent Li deposition regulation capability of HE‐PBA, which enables uniform Li deposition and effectively suppresses dendrite formation. On this basis, Figure 22K presents the practical application of the HE‐PBA/K^+^‐ISE‐based wearable potassium ion sensor for real‐time sweat monitoring. By adhering the flexible sensor to the forearm skin of a volunteer, the system enables continuous potentiometric monitoring of K^+^ concentration during a 30‐minute running session. The results show that the average K^+^ concentration detected was approximately 7 mM, which is highly consistent with the results obtained via ICP‐MS analysis, thus validating the accuracy and reliability of the system under dynamic physiological conditions.^[^ ^113^ ^]^

## 6 Summary and Perspective

Over the past decade, Li–S batteries have emerged as one of the most promising next‐generation energy storage systems, owing to their exceptionally high theoretical energy density and low material cost. However, the practical commercialization of Li–S batteries remains impeded by several intrinsic challenges, including sluggish redox kinetics of sulfur species, the shuttle effect of LiPS, and the poor reversibility of Li_2_S conversion. To address these issues, the incorporation of advanced electrocatalysts into the Li–S battery system has proven to be an effective strategy for enhancing electrochemical performance. In this context, high‐entropy catalysts, featuring multi‐component compositions and entropy‐stabilized structures, offer a novel and efficient approach to alleviating the complex bottlenecks in Li–S batteries. Recent studies have demonstrated that the synergistic interaction among multiple metal elements in HEC can modulate the electronic structure, lower reaction barriers, and improve the anchoring of LiPS intermediates, thereby enhancing their catalytic functionality. These advantages make HEC highly promising for accelerating sulfur species redox reactions, suppressing the shuttle effect, and improving long‐term cycling stability.

This review provides a comprehensive summary of the definition, application advantages, and preparation strategies of HEC, with a particular emphasis on their recent advances and evolving roles in high‐performance Li–S batteries. Inspired by the remarkable progress of high‐entropy alloys in the field of energy conversion, HEC have undergone rapid development with increasingly diverse elemental compositions and structural architectures. The high‐entropy concept has been extended to various classes of metal compounds, including high‐entropy oxides, nitrides, sulfides, phosphides, MXenes, and metal‐organic frameworks and their derivatives. Owing to the presence of multi‐metallic active sites and tunable electronic structures, these HEC exhibit outstanding catalytic performance in Li–S batteries by effectively anchoring LiPS and facilitating sulfur redox kinetics. Moreover, the high configurational entropy enables HEC to maintain superior structural integrity under harsh electrochemical conditions, outperforming traditional single‐ or bi‐metallic catalysts. Despite these promising developments, the application of HEC in Li–S batteries is still in its early stages. The introduction of multiple elements, while beneficial for catalytic diversity, also complicates the already ambiguous redox mechanisms of Li–S systems, making mechanistic interpretation more challenging. Furthermore, a lack of systematic theoretical models and in‐depth mechanistic studies has hindered the full understanding and rational design of HEC in this context. Therefore, there remains significant room for improvement and further exploration. Future research directions may include:

1. The high‐entropy concept, while traditionally associated with solid‐solution oxides, alloys, or chalcogenides, holds significant potential for expansion into emerging material platforms such as MOF and single‐atom catalysts (SAC). High‐entropy MOF, by incorporating multiple metal nodes into a crystalline porous framework, can inherit both the entropic stabilization benefits and the tunability of MOF chemistry. These materials offer precise control over metal coordination environments, high surface areas, and well‐defined porosity, all of which are advantageous for LiPS confinement and catalytic conversion in Li–S batteries. Moreover, HE‐MOFs serve as excellent precursors for the derivation of high‐entropy porous carbons, metal sulfides, or oxides with preserved compositional complexity and hierarchical structures. On the other hand, the development of high‐entropy single‐atom catalysts (HE‐SAC) is a promising frontier that combines the atomic dispersion of active sites with the synergistic effects of multiple elements. Such systems may provide ultra‐high atomic utilization and programmable coordination chemistry potentially surpassing conventional SAC in catalytic selectivity and robustness. Nevertheless, the synthesis and stabilization of HE‐SAC remain challenging, particularly in preventing agglomeration and ensuring uniform distribution on support materials. Future efforts should explore MOF‐derived strategies, defect engineering, and entropy‐driven anchoring mechanisms to enable robust high‐entropy SAC frameworks. These emerging high‐entropy material classes offer reference alternative strategies for HEC design.
2. Despite growing interest in HEC, their catalytic behavior in Li–S systems is not yet fully understood. The multicomponent nature of HEC introduces a high degree of complexity, including elemental distribution heterogeneity, local coordination variability, and dynamic surface transformations during cycling. Future work should prioritize the application of advanced in situ and operando techniques, such as XPS, X‐ray absorption spectroscopy (XAFS), and Raman spectrum, to directly monitor the evolution of oxidation states, bonding environments, and structural motifs during sulfur redox processes. These methods will be critical to deciphering how entropy‐derived effects influence catalytic activity and structural stability, and to distinguish between intrinsic catalytic contributions and surface adsorption effects. Moreover, time‐resolved studies under realistic operating conditions can clarify the dynamic interplay between LiPS adsorption, activation energy reduction, and Li_2_S precipitation pathways. A deeper mechanistic understanding informed by these advanced diagnostics will ultimately enable the rational design of next‐generation HEC with tailored catalytic functionalities and enhanced electrochemical durability.
3. Given the virtually infinite compositional space of high‐entropy catalysts (HECs), traditional trial‐and‐error approaches are increasingly inadequate for efficient material discovery. Future research is expected to rely more heavily on artificial intelligence (AI) and machine learning (ML) frameworks to accelerate the design and optimization of HEC tailored for Li–S battery systems. At the chemical composition level, ML models trained on known materials databases and DFT‐derived properties can rapidly predict formation enthalpies, phase stability, and LiPS adsorption energies across thousands of hypothetical multi‐element combinations. Moreover, descriptor‐based models utilizing parameters such as atomic size mismatch, electronegativity difference, and valence electron concentration have already shown good correlation with catalytic performance in various systems. Beyond static property prediction, emerging techniques such as graph neural networks (GNNs) offer improved accuracy in predicting complex processes like electronic structure, charge transfer pathway, or even dynamic phase evolution. Moreover, AI is poised to revolutionize experimental workflows through the integration of automated synthesis platforms, robotic testing systems, and self‐optimizing experimental loops. Within such closed‐loop frameworks, theoretical models iteratively guide synthesis and characterization, while real‐time feedback continuously refines model predictions. This dynamic optimization significantly shortens the development cycle for high‐performance HEC. In the context of Li–S batteries, AI can further contribute by modeling and simulating complex multi‐interface interactions, electrolyte compatibility, and structural evolution during extended cycling. By integrating multi‐scale simulations, from the atomic to the electrode level, with AI‐driven predictive models, researchers can gain a more comprehensive understanding of the catalytic behavior of HEC within full‐cell systems.
4. The catalytic activity of HEC is not solely determined by their compositional diversity, but is also profoundly influenced by their multiscale structural characteristics. At the atomic scale, factors such as local lattice distortions, strain fields, and a high density of intrinsic defects can create unsaturated coordination sites that enhance LiPS adsorption and accelerate redox kinetics. At the nanoscale, deliberate control over morphology, such as designing hollow spheres, nanorods, or two‐dimensional layers, can effectively increase the specific surface area and improve electrolyte contact and active site accessibility. At the mesoscale, constructing hierarchically porous architectures facilitates rapid Li ion diffusion and enhances electrolyte infiltration during cycling, thereby contributing to improved long‐term electrochemical stability. Moving forward, future research should integrate these structural design principles with compositional optimization to achieve synergistic performance gains. Additionally, the rational design of heterostructured or core‐shell‐type HEC may allow multiple functional domains within a single catalytic framework, offering new avenues for multifunctional catalyst development in Li–S batteries.
5. Although HEC are widely recognized for their structural robustness and entropy‐stabilized single‐phase frameworks, their long‐term electrochemical stability under realistic Li–S battery conditions remains insufficiently understood. In practical full‐cell environments, HEC may be subject to complex degradation mechanisms, especially under high sulfur loading, lean electrolyte, high temperature and long‐term cycle conditions. These include surface leaching triggered by reactive LiPS, passivation of catalytic active sites by the accumulation of insulating discharge products such as Li_2_S, and redox‐driven surface reconstruction or elemental redistribution, all of which can compromise catalytic performance over time.

Although the high configurational entropy of HEC promotes the formation of homogeneous solid solutions and enhances thermodynamic phase stability, it cannot by itself suppress kinetically driven degradation processes. This limitation becomes especially pronounced under dynamic electrochemical cycling, where continuous redox activity and repeated phase transformations can destabilize the catalyst surface. In particular, nanoscale HEC, which offer high surface‐to‐volume ratios and abundant active sites, are more prone to structural instability due to elevated surface energy and increased chemical reactivity. To support practical applications, future research should prioritize the development of advanced in situ characterization techniques, such as XAFS, XRD, XPS, and Raman spectroscopy, to verify the structural integrity and compositional uniformity of HECs under real‐time operating conditions. These tools can provide crucial insight into local structural evolution, redox behavior, and degradation pathways. Furthermore, it is essential to explore material design strategies that enhance operational durability without compromising catalytic performance. These may include introducing surface protective layers to mitigate LiPS corrosion, construction of heterostructures to stabilize catalyst interfaces, and incorporation of corrosion‐resistant, redox‐inert elements (e.g., Ti, Mo, or Zr) to prevent surface degradation.

1. While many studies demonstrate promising results in Li–S coin cells or lab‐scale half‐cell configurations, significant challenges remain in translating HEC to real‐world systems. The application of high‐entropy materials should not be confined to enhancing intrinsic material properties, but should extend to solving key bottlenecks under practical operating conditions. To meet industrial standards, lithium–sulfur batteries are expected to achieve sulfur loadings > 5 mg cm^−2^, electrolyte‐to‐sulfur (E/S) ratios < 5 µL mg^−1^, and negative‐to‐positive (N/P) capacity ratios < 2.5, while still maintaining high areal capacities (> 6 mAh cm^−2^) and long cycle life (> 300 cycles). HEC with their robust structural frameworks, tunable surface chemistry, and multi‐element synergy, offer promising pathways to meet these stringent targets. Future efforts should focus on integrating HEC into multifunctional electrode architectures capable of operating under lean electrolyte and high loading conditions, while simultaneously facilitating sulfur species conversion and minimizing electrochemical polarization.

Beyond their use in cathode catalysis, HEC can also be extended to other functional components of Li–S batteries, such as separators and electrode interfacial modification layers. Notably, the intrinsic multi‐metallic composition of HEC offers significant advantages for protecting the metallic Li anode. By incorporating elements with strong chemical polarity and high lithiophilicity as principal components (e.g., Co, Ni, Cu, etc.), a multifunctional HEC design may be achieved to further optimize the electrochemical performance of Li–S full cells. In addition, due to their inherently high configurational entropy, HEC exhibit stable catalytic activity and strong environmental tolerance under extreme operating conditions. Thereby, the development of pouch cells incorporating HEC‐based designs and the evaluation of their electrochemical reliability across a wide temperature range may help expand the practical application scenarios of Li–S batteries.

Moreover, it is important to note that most high‐entropy catalysts reported to date are composed of dense inorganic metal compounds (e.g., oxides, carbides, alloys), which typically exhibit relatively high mass densities. Consequently, their incorporation into the cathode must be carefully optimized to avoid excessive inactive weight, which could diminish the overall gravimetric energy density of the full cell. To address this challenge, future design strategies should aim to minimize the loading of high‐entropy materials while maximizing their intrinsic catalytic efficiency. Approaches such as constructing nanoscale architectures (e.g., hollow spheres, nanosheets, porous frameworks), integrating with lightweight conductive matrices (e.g., graphene, carbon nanotubes), and engineering high surface‐area active sites can significantly enhance mass‐specific catalytic activity. Through such structural optimizations, it is feasible to achieve high catalytic performance with minimal material usage, thereby balancing catalytic functionality with battery energy density.

1. To enable the widespread adoption of HEC in practical Li–S battery applications, synthesis techniques must be scalable, controllable, and environmentally sustainable. Traditional methods such as high‐temperature annealing, arc melting, or mechanical alloying, while effective at the laboratory scale, are often energy‐intensive, equipment‐dependent, and challenging to scale up for industrial production. Therefore, alternative synthetic strategies, including spray pyrolysis, microwave‐assisted synthesis, sol–gel combustion, and molten salt techniques, hold great promise for achieving cost‐effective, uniform, and large‐batch fabrication of high‐entropy materials. These methods can offer improved control over particle morphology, phase composition, and microstructure while reducing overall energy consumption. Additionally, environmental concerns associated with acid leaching, toxic solvents, or high‐temperature emissions call for greener synthesis strategies. Therefore, it is essential to integrate the principles of green chemistry into synthesis design. This includes the use of solvent‐free or low‐toxicity aqueous systems, renewable or low‐impact precursors, and processes with minimal waste generation. However, scalability must not come at the expense of compositional precision; maintaining a homogeneous elemental distribution and consistent physicochemical properties across batches is critical to ensure reproducible catalytic performance.
2. Although this review primarily focuses on the role of HEC in enhancing sulfur redox kinetics and stabilizing the cathode in Li–S batteries, it is worth noting that the high‐entropy concept can be further extended to other key components of the Li–S battery system, particularly the electrolyte. In liquid electrolyte systems, researchers have begun designing high‐entropy electrolytes by incorporating multiple Li salts and solvents to construct multicomponent solvation environments. These systems introduce configurational entropy at the molecular level, enhancing the diversity of Li ions solvation structures and improving compatibility with polysulfide intermediates. Entropic mixing of solvents (e.g., ethers, nitriles, fluorinated compounds) and salts (e.g., LiTFSI, LiFSI, LiNO_3_) can effectively suppress the shuttle effect while promoting the stability of redox reactions. Similarly, in composite gel or polymer electrolytes, the introduction of multi‐polymer matrices or mixed lithium salt systems can induce local entropy‐driven disorder. This enhances ionic conductivity by broadening conduction channels, increasing ion mobility, and improving mechanical integrity. These characteristics are particularly beneficial for suppressing lithium dendrite formation and maintaining interfacial stability in flexible or quasi‐solid‐state structures. High‐entropy engineering also shows promise in the development of solid‐state electrolytes. For instance, doping five or more heterovalent or isovalent cations (e.g., Li, La, Y, Zr, Al) into sulfide or oxide frameworks can enhance ionic mobility and phase stability across a wider temperature range. Moreover, entropy‐stabilized solid electrolytes have demonstrated potential in reducing interfacial resistance with lithium metal and expanding the electrochemical stability window, factors that are critical for the realization of high‐performance Li–S batteries. Overall, these entropy‐controlled electrolyte strategies serve as a valuable complement to the catalytic benefits provided by HEC, offering a more holistic approach to improving the performance and reliability of Li–S battery systems.

HEC represent a powerful new paradigm for addressing the intricate electrochemical limitations of Li–S batteries. With continued interdisciplinary efforts bridging materials design, mechanistic understanding, and device‐level engineering, HEC are poised to become cornerstone components in the development of next‐generation, high‐performance, and durable Li–S energy storage systems. This review has highlighted the fundamental principles, synthesis strategies, and emerging applications of HEC in Li–S battery systems, with the goal of stimulating further research interest and accelerating the transition of Li–S batteries from laboratory studies to practical deployment.

## Conflict of Interest

The authors declare no conflict of interest.
